# Modified FOLFOXIRI plus cetuximab versus bevacizumab in *RAS* wild-type metastatic colorectal cancer: a randomized phase II DEEPER trial

**DOI:** 10.1038/s41467-024-54460-2

**Published:** 2024-11-25

**Authors:** Manabu Shiozawa, Yu Sunakawa, Takanori Watanabe, Hirofumi Ota, Hisateru Yasui, Taichi Yabuno, Mitsuyoshi Tei, Mitsugu Kochi, Dai Manaka, Hisatsugu Ohori, Tatsuro Yamaguchi, Tamotsu Sagawa, Masahito Kotaka, Yutaro Kubota, Takashi Sekikawa, Masato Nakamura, Masahiro Takeuchi, Wataru Ichikawa, Masashi Fujii, Akihito Tsuji

**Affiliations:** 1 Department of Gastrointestinal Surgery, Kanagawa Cancer Center, Yokohama, Japan; 2 Department of Clinical Oncology, St. Marianna University School of Medicine, Kawasaki, Japan; 3 Department of Surgery, Tokushima Municipal Hospital, Tokushima, Japan; 4 Department of Gastroenterological Surgery, Ikeda City Hospital, Ikeda, Japan; 5 Department of Medical Oncology, Kobe City Medical Center General Hospital, Kobe, Japan; 6 Department of Gastroenterological Surgery, Yokohama Municipal Citizen’s Hospital, Yokohama, Japan; 7 Department of Surgery, Osaka Rosai Hospital, Osaka, Japan; 8 Department of Hepato-Biliary-Pancreatic and Gastrointestinal Surgery, International University of Health and Welfare, School of Medicine, Otawara, Japan; 9 Department of Surgery, Gastro-Intestinal Center, Kyoto Katsura Hospital, Kyoto, Japan; 10 Department of Medical Oncology, Ishinomaki Red Cross Hospital, Ishinomaki, Japan; 11 Department of Clinical Genetics, Tokyo Metropolitan Cancer and Infectious Diseases Center, Komagome Hospital, Tokyo, Japan; 12 Division of Gastroenterology, National Hospital Organization Hokkaido Cancer Center, Sapporo, Japan; 13 Gastrointestinal Cancer Center, Sano Hospital, Kobe, Japan; 14 Department of Clinical Oncology, Showa University Hospital, Tokyo, Japan; 15 Division of Medical Oncology, Showa University Fujigaoka Hospital, Yokohama, Japan; 16 Aizawa Comprehensive Cancer Center, Aizawa Hospital, Matsumoto, Japan; 17 Graduate School of Mathematical Sciences, The University of Tokyo, Tokyo, Japan; 18 Japan Clinical Cancer Research Organization, Tokyo, Japan; 19 Department of Clinical Oncology, Faculty of Medicine, Kagawa University, Kagawa, Japan

**Keywords:** Colorectal cancer, Chemotherapy, Randomized controlled trials

## Abstract

The clinical significance of FOLFOXIRI (5-FU, leucovorin, oxaliplatin, and irinotecan) plus anti-EGFR monoclonal antibody using cetuximab for metastatic colorectal cancer (mCRC) remains controversial. We report results from a randomized phase 2 DEEPER trial (UMIN000018217, jRCTs061180022) to test the superiority of modified (m)-FOLFOXIRI plus weekly cetuximab over bevacizumab in patients with *RAS* wild-type (wt) mCRC. Primary endpoint was depth of response (DpR). Secondary endpoints included objective response rate (ORR), early tumor shrinkage (ETS) at week 8, progression-free survival (PFS), overall survival (OS), time to tumor growth (TTG), time to treatment failure (TTF), association between tumor shrinkage and prognosis, association between TTG and prognosis, R0 resection rate, and safety. In 359 enrolled patients with *RAS* wt mCRC, median DpR was significantly better in cetuximab (57.3% vs 46.0%, *p* = 0.0029); however, ORR, ETS, R0 resection rate, TTG, TTF, PFS and OS were similar between 2 treatments. There was a weak association between DpR and survival time in both treatments. The correlation between TTG and OS was slightly stronger in cetuximab. The post-hoc exploratory analysis showed that cetuximab produced greater PFS (15.3 vs 11.7 months; HR 0.68) and OS (53.6 vs 40.2 months; HR 0.54) in patients with left-sided and *RAS*/*BRAF* wt tumors. m-FOLFOXIRI plus cetuximab has clinical benefit for tumor shrinkage in *RAS* wt mCRC. The survival benefit for *RAS*/*BRAF* wt and left-sided mCRC needs further investigation.

## Abstract (web summary)

Anti-EGFR therapy plus doublet chemotherapy is standard of care for patients with *RAS* wild-type metastatic colorectal cancer (mCRC) but the role of triplet chemotherapy is unclear. Here, the authors report a randomised phase 2 trial testing the superiority of adding cetuximab (anti-EGFR) over bevacizumab (anti-VEGF) to modified FOLFOXIRI (5-FU, leucovorin, oxaliplatin and irinotecan) in patients with *RAS* wild-type mCRC.

## Introduction

A pooled retrospective analysis of data from 6 clinical trials showed a significant benefit with chemotherapy plus EGFR therapy compared to bevacizumab on overall survival (OS) in metastatic colorectal cancer (mCRC) patients with *RAS* wild-type and left-sided tumors^1^. Recently, a prospective phase 3 trial demonstrated a statistically significant survival benefit with the anti-EGFR monoclonal antibody (mAb), panitumumab, compared with bevacizumab added to standard first-line chemotherapy (5-fluorouracil [5-FU], leucovorin and oxaliplatin [FOLFOX]) in *RAS* wild-type and left-sided mCRC^2^. Based on these findings, the current standard of care for patients with untreated, left-sided, *RAS*/*BRAF* wild-type mCRC is doublet chemotherapy with an anti-EGFR mAb (either cetuximab or panitumumab)^3,4^. However, the use of triplet chemotherapy with anti-EGFR mAbs remains controversial.

The TRIPLETE study investigated the combination of modified (m)-FOLFOX6 + panitumumab versus m-FOLFOXIRI (5-FU, leucovorin, oxaliplatin, and irinotecan) + panitumumab in patients with untreated *RAS*/*BRAF* wild-type mCRC^5^. The study did not find significant improvement in outcomes when panitumumab was combined with triplet compared with doublet chemotherapy, with objective response rate (ORR) of 73% versus 76%^5^. However, the frequency of grade 3 or more diarrhea in the triplet group was 23%, and the frequency of grade 3 or more rash acneiform in the doublet group was 29% versus 19% in the triplet group. These findings suggest that the dose intensity of panitumumab may be lower due to toxicity in the triplet group. The VOLFI trial demonstrated strong tumor shrinkage with triplet plus panitumumab for *RAS* wild-type mCRC^6^, so it would be worthwhile to further evaluate triplet plus cetuximab, which is a distinct anti-EGFR mAb from panitumumab in the aspect of administration method and clinical pharmacology^7^. Also, the FIRE-4.5 trial was the first prospective and randomized study to investigate m-FOLFOXIRI + cetuximab versus m-FOLFOXIRI + bevacizumab as initial treatment for *BRAF* V600E mutant mCRC, with no improvement of ORR in the cetuximab group^4^. Therefore, the efficacy of m-FOLFOXIRI + cetuximab therapy for *RAS*/*BRAF* wild-type mCRC is promising.

The DEEPER trial (JACCRO CC-13) was designed to test the superiority of m-FOLFOXIRI + cetuximab over m-FOLFOXIRI + bevacizumab, which is one of standard chemotherapy regimens, in patients with unresectable *RAS* wild-type mCRC. Here, we present the primary analysis of the DEEPER study.

## Results

**Fig. 1:**
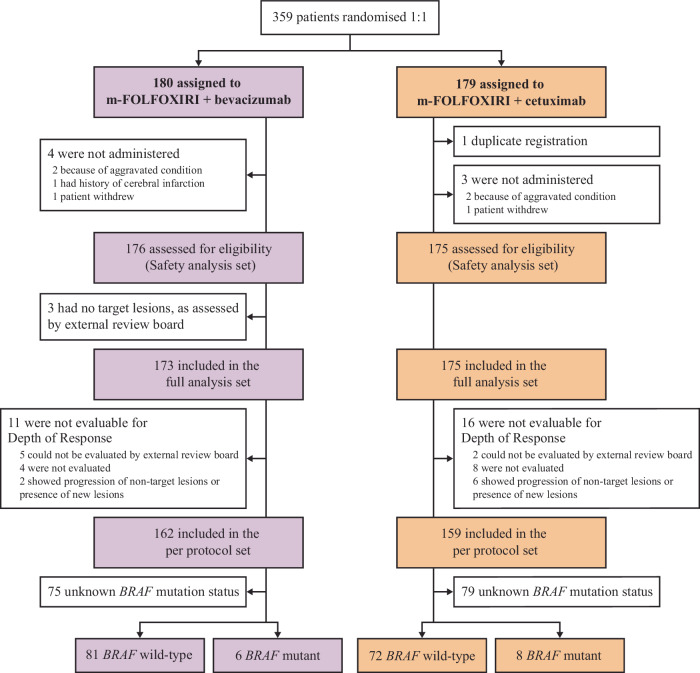
Patient flow chart in the DEEPER trial. CONSORT diagram illustrating patient inclusion.

Between 1 July 2015 and 30 June 2019, 359 patients with *RAS* wild-type, unresectable mCRC were randomly assigned to receive m-FOLFOXIRI + cetuximab (*n* = 179) or m-FOLFOXIRI + bevacizumab (*n* = 180) (Fig. 1). Overall, 351 patients received at least one dose of study treatment and were included in the safety population (cetuximab: *n* = 175; bevacizumab: *n* = 176). There were 16 patients for the cetuximab arm and 14 patients for the bevacizumab arm, who were not evaluable for depth of response (DpR). In the per protocol set (PPS) cohort, 159 and 162 patients were included in the cetuximab and bevacizumab arm, respectively. The cut-off date for the current analysis was 2 August 2022.

Baseline characteristics of the PPS were well balanced (Table 1). The number of metastases, metastatic sites, and the number of cases with liver-limited disease were comparable between the two groups. Based on *BRAF* testing performed in routine clinical practice, the *BRAF* V600E mutation status of 183 patients (51% of patients enrolled overall) was determined.

**Table 1:** Baseline characteristics

	m-FOLFOXIRI + cetuximab (*n* = 159)	m-FOLFOXIRI + bevacizumab (*n* = 162)
Sex, *n* (%)		
Male/Female	101 (63.5)/58 (36.5)	106 (65.4)/56 (34.6)
Age (years), median (range)	65 (26–83)	65 (29–85)
ECOG performance status, *n* (%)		
0/1	146 (91.8)/13 (8.2)	146 (90.1)/16 (9.9)
Adjuvant chemotherapy, *n* (%)		
None/With oxaliplatin/Without oxaliplatin	148 (93.1)/7 (4.4)/4 (2.5)	152 (93.8)/6 (3.7)/4 (2.5)
Primary tumor site, *n* (%)		
Colon/Rectum	91 (57.2)/68 (42.8)	93 (57.4)/69 (42.6)
Site of primary tumor, *n* (%)		
Right/Left	27 (17.0)/132 (83.0)	25 (15.4)/137 (84.6)
Resected primary tumor, *n* (%)		
Yes/No	83 (52.2)/76 (47.8)	86 (53.1)/76 (46.9)
Time to metastases, *n* (%)		
Synchronous/Metachronous	136 (85.5)/23 (14.5)	136 (84.0)/26 (16.0)
Number of metastatic sites, *n* (%)		
0-1/ > 1	55 (34.6)/104 (65.4)	80 (49.4)/82 (50.6)
Liver-only disease, *n* (%)		
Yes/No	39 (24.5)/120 (75.5)	50 (30.9)/112 (69.1)
Metastatic sites, *n* (%)		
Liver	119 (74.8)	116 (71.6)
Lung	44 (27.7)	37 (22.8)
Lymph node	98 (61.6)	81 (50.0)
Peritoneum	31 (19.5)	25 (15.4)
Bone	3 (1.9)	4 (2.5)
*BRAF* V600E status		
wild / mutant/unknown	72/8/79	81/6/75
*UGT1A1* (*6, *28), *n* (%)		
Wild/hetero/homo & double hetero/unknown	65 (40.9)/59 (37.1)/9 (5.7)/26 (16.4)	69 (42.6)/46 (28.4)/22 (13.6)/25 (15.4)

*ECOG* Eastern Cooperative Oncology Group, *m-FOLFOXIRI* modified 5-FU, leucovorin, oxaliplatin and irinotecan.

After 8 cycles of treatment, relative dose intensity was 77.1% in the cetuximab arm versus 71.7% in the bevacizumab arm. Eighty-three percent of patients received one dose of cetuximab weekly for up to 8 cycles. Most patients received treatment post-trial, during the follow-up period. In the cetuximab arm, 39% of patients underwent secondary resection of metastases and 54% of received second-line chemotherapy with bevacizumab. In the bevacizumab arm, 35% of patients underwent secondary resection of metastases and 59% of received second-line chemotherapy with anti-EGFR mAb. In both arms, subsequent chemotherapy regimens were irinotecan-based (54–65%), oxaliplatin-based (7–10%), FOLFOXIRI-based (8–15%), or 5-FU-based (8–10%).

### Tumor response

**Fig. 2:**
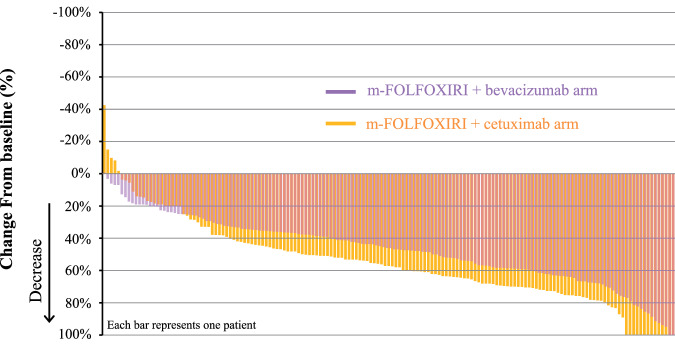
Depth of response in the per protocol set. A t-test with Welch’s adjusted degree of freedom was used.

With a median follow-up of 51.8 months, the median DpR in the PPS as a primary endpoint was 57.3% for m-FOLFOXIRI + cetuximab and 46.0% for m-FOLFOXIRI + bevacizumab arms (*p* = 0.0029) (Table 2; Fig. 2). The median DpR at 4 months was also better in the cetuximab arm (53.3% vs. 41.2%, *p* = 0.0083). The ORR was 71.1% (95% CI 64.0–78.1) and 69.1% (95% CI 62.0–76.2) for cetuximab and bevacizumab, respectively (chi-squared test, *p* = 0.71); disease control rate was 98.7% (95% CI 97.0–100) and 99.4% (95% CI 98.2–100), respectively (chi-squared test, *p* = 0.55). Early tumor shrinkage (ETS) rate was 79.9% for cetuximab and 74.7% for bevacizumab (chi-squared test, *p* = 0.27). R0 resection rate was 26.4% (95% CI 19.6–33.3) and 21.0% (95% CI 14.7–27.3) for cetuximab and bevacizumab, respectively. Median time to tumor growth (TTG), as time to tumor size nadir, was 7.5 months for cetuximab and 7.4 months for bevacizumab (*p* = 0.19). Median time to treatment failure was 11.0 months for cetuximab and 10.8 months for bevacizumab (*p* = 0.22).

**Table 2:** Tumor response

		m-FOLFOXIRI + cetuximab (*n* = 159)	m-FOLFOXIRI + bevacizumab (*n* = 162)
Median DpR (range)		57.3% (−42.6–100)	46.0% (−0.6–100)
Mean (95% CI)		55.2% (51.1–59.3)	47.3% (44.1–50.5)
Standard deviation (95% CI)		26.17 (23.6–29.4)	20.53 (18.5–23.0)
Welch’s t-test		*p* = 0.0029	
Best response	Complete response	8 (5.0%)	3 (1.9%)
	Partial response	105 (66.0%)	109 (67.3%)
	Stable disease	44 (27.7%)	49 (30.2%)
	Progressive disease	2 (1.3%)	0 (0%)
	Not evaluated	0 (0%)	1 (0.6%)
Objective response rate (95% CI)		71.1% (64.0–78.1)	69.1% (62.0–76.2)
Chi-squared test		*p* = 0.71	
Disease control rate (95% CI)		98.7% (97.0–100)	99.4% (98.2–100)
Chi-squared test		*p* = 0.55	
R0 resection rate (95% CI)		26.4% (19.6–33.3)	21.0% (14.7–27.3)
Chi-squared test		*p* = 0.25	

Chi-squared test was used for the statistical analysis.

*CI* confidence interval, *DpR* depth of response, *m-FOLFOXIRI* modified 5-FU, leucovorin, oxaliplatin and irinotecan.

An exploratory subgroup analysis of the DpR by clinical factors indicated that the cetuximab arm had significant benefits (*p* < 0.01) for patients who were male, had Eastern Cooperative Oncology Group (ECOG) performance status (PS) of 0, synchronous metastases, and a left-sided primary tumor (Supplementary Table 1). Among patients with left-sided tumors in the PPS, median DpR was 59.2% in the cetuximab arm (*n* = 132) and 46.1% in the bevacizumab arm (*n* = 137) (*p* = 0.0026) (Supplementary Table 2). Among patients with right-sided tumors, median DpR was 50.0% and 41.2% in the cetuximab arm (*n* = 27) and the bevacizumab arm (*n* = 25), respectively (*p* = 0.47) (Supplementary Table 3).

### Survival

**Fig. 3:**
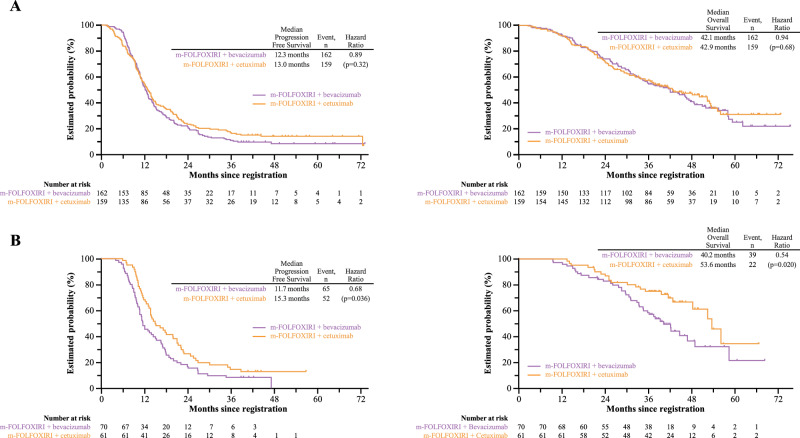
Progression-free survival and overall survival in each patient cohort. **A** Patients with *RAS* wild-type metastatic colorectal cancer of the per protocol set, (**B**) Patients with *RAS*/*BRAF* wild-type, left-sided tumors. A log-rank test was used with a two-sided significance.

In the PPS, median progression-free survival (PFS) was 13.0 months in the cetuximab arm and 12.3 months in the bevacizumab arm (HR 0.89; *p* = 0.32); median overall survival (OS) was 42.9 months and 42.1 months, respectively (HR 0.94; *p* = 0.68) (Fig. 3A). Of the patients with left-sided tumors, median PFS was 13.8 months and 12.1 months with cetuximab and bevacizumab (HR 0.80; *p* = 0.090) and median OS was 48.2 months and 42.1 months (HR 0.89; *p* = 0.48), respectively (Supplementary Table 2). Of the patients with right-sided tumors, median PFS was 9.0 months and 12.8 months (HR 1.54; *p* = 0.14), and median OS was 28.4 months and 33.9 months, respectively (HR 1.15; *p* = 0.68) (Supplementary Table 3; Supplementary Fig. 1).

There was a weak association between DpR and survival time in the PPS population. The spearman’s rank correlation showed *r* = 0.35 for cetuximab and *r* = 0.29 for bevacizumab in term of PFS; *r* = 0.33 for cetuximab and *r* = 0.31 for bevacizumab in term of OS. The correlation between TTG and OS was slightly stronger in the cetuximab arm than in the bevacizumab arm (*r* = 0.42 versus *r* = 0.20).

### Outcomes in *RAS*/*BRAF* wild-type tumors

Of the 359 enrolled patients, *BRAF* status was evaluable in 183 patients. *BRAF* V600E mutation was identified in 14 (4.4%) of 167 patients of the PPS. We performed an exploratory analysis of clinical outcomes according to *BRAF* status, which was not predefined in the study protocol. Among 153 patients with *RAS*/*BRAF* wild-type tumors in the PPS, median DpR was 60.4% with m-FOLFOXIRI + cetuximab and 47.9% with m-FOLFOXIRI + bevacizumab (*p* = 0.007). Median PFS was 13.8 months versus 12.3 months (HR 0.80, 95% CI 0.57–1.12) and median OS was 53.4 months versus 42.1 months (HR 0.67, 95% CI 0.42–1.07), respectively. Of 131 patients with *RAS*/*BRAF* wild-type, left-sided disease, median DpR was 63.6% with cetuximab and 47.8% with bevacizumab (*p* = 0.0003). ORR was 83.6% with cetuximab and 72.9% with bevacizumab (*p* = 0.14). Median PFS was 15.3 months versus 11.7 months (HR 0.68, 95% CI 0.47–0.98) and median OS was 53.6 months versus 40.2 months (HR 0.54, 95% CI 0.32–0.91), respectively (Supplementary Table 2; Fig. 3B). Of patients with *RAS*/*BRAF* wild-type, right-sided disease, median DpR was 44.7% with cetuximab and 50.0% with bevacizumab (*p* = 0.45). Median PFS was 7.4 months versus 13.2 months (HR 2.05, 95% CI 0.84–5.01) and median OS was 27.3 months versus 47.7 months (HR 1.78, 95% CI 0.58–5.46), respectively (Supplementary Table 3; Supplementary Fig. 2).

### Tumor resection after chemotherapy

R0 resection was performed in 76 of 321 patients in the PPS. Of the patients in each arm, median OS also was longer in patients with R0 resection compared to those with non-R0 resection (Supplementary Fig. 3). The post-hoc analysis for patients with *RAS*/*BRAF* wild-type and left-sided tumors indicated that median OS was longer in the cetuximab arm compared to the bevacizumab arm (53.6 months versus 35.7 months, HR 0.55) in patients other than R0 resection but not patients with R0 resection (Supplementary Fig. 4).

### Safety

Any-grade adverse events (AEs) occurred in 99.4% of patients treated with m-FOLFOXIRI + cetuximab and 100% of patients treated with m-FOLFOXIRI + bevacizumab (Table 3). The most common AE was neutropenia in both arms, which was Grade 3–4 in 56.0% and 54.5% of the cetuximab and bevacizumab arms, respectively. Other common AEs were rash acneiform (Grade 1–2: 63.4%; Grade 3–4: 13.1%) and neurotoxicity (Grade 1–2: 68.0%; Grade 3–4: 6.9%) with cetuximab, and neurotoxicity (Grade 1–2: 67.6%; Grade 3–4: 4.5%) and hypertension (Grade 1–2: 36.9%; Grade 3–4: 33.5%) with bevacizumab.

**Table 3:** Adverse events of interest by grade

	m-FOLFOXIRI + cetuximab (*n* = 175)		m-FOLFOXIRI + bevacizumab (*n* = 176)	
	Grade 1–2 *n* (%)	Grade 3–4 *n* (%)	Grade 1–2 *n* (%)	Grade 3–4 *n* (%)
Any event	24 (13.7)	150 (85.7)	22 (12.5)	154 (87.5)
Anaemia	79 (45.1)	11 (6.3)	94 (53.4)	8 (4.5)
Thrombocytopenia	78 (44.6)	4 (2.3)	69 (39.2)	2 (1.1)
Nausea	72 (41.1)	11 (6.3)	78 (44.3)	11 (6.3)
Vomiting	17 (9.7)	1 (0.6)	28 (15.9)	3 (1.7)
Diarrhea	85 (48.6)	21 (12.0)	87 (49.4)	14 (8.0)
Stomatitis	85 (48.6)	17 (9.7)	71 (40.3)	4 (2.3)
Neutropenia	56 (32.0)	98 (56.0)	58 (33.0)	96 (54.5)
Febrile neutropenia	–	15 (8.6)		19 (10.8)
Neurotoxicity	119 (68.0)	12 (6.9)	119 (67.6)	8 (4.5)
Fatigue	46 (26.3)	12 (6.9)	53 (30.1)	11 (6.3)
Anorexia	87 (49.7)	21 (12.0)	80 (45.5)	19 (10.8)
Fever	23 (13.1)	0	40 (22.7)	0
Alopecia	64 (36.6)	–	70 (39.8)	–
Hand-foot syndrome	15 (8.6)	5 (2.9)	8 (4.5)	1 (0.6)
Rash acneiform	111 (63.4)	23 (13.1)	5 (2.8)	0
Hypomagnesaemia	92 (52.6)	7 (4.0)	31 (17.6)	0
Hypokalaemia	51 (29.1)	23 (13.1)	35 (19.9)	11 (6.3)
Hypertension	54 (30.9)	31 (17.7)	65 (36.9)	59 (33.5)

*m-FOLFOXIRI* modified 5-FU, leucovorin, oxaliplatin and irinotecan.

## Discussion

DEEPER study demonstrated significant improvements in DpR with manageable safety in mCRC patients with *RAS* wild-type tumors treated with m-FOLFOXIRI + cetuximab as initial therapy compared with m-FOLFOXIRI + bevacizumab; however, survival time was comparable between the 2 arms. Our study met the primary endpoint but failed to demonstrate a prolonged prognosis of m-FOLFOXRI + cetuximab. The targeting population, *RAS* wild-type tumors, includes *BRAF* V600E-mutated or right-sided primary cases who are unlikely to benefit from anti-EGFR mAb drugs; therefore, survival may not be properly assessed. Post-hoc subgroup analysis for patients with *RAS*/*BRAF* wild-type and left-sided tumors showed a trend toward longer OS in the cetuximab group (53.6 months versus 40.2 months, HR 0.54, 95% CI 0.32–0.91), although this was an exploratory subgroup analysis without multiplicity adjustment. The triplet plus cetuximab regimen has a strong tumor shrinkage effect, but its use and targeting need further investigation.

In our study, the DpR was met as primary endpoint, which is an indicator of the rate of chemotherapy-induced tumor shrinkage. While DpR has been shown to correlate with post-progression survival and OS in previous retrospective analyses of clinical trials^8^, this correlation has also been demonstrated in subgroup analyses of prospective studies including the CRYSTAL and OPUS trials^9–11^. Based on these findings, we set DpR as the primary endpoint in the DEEPER trial because the DpR may be a promising surrogate for OS. A previous pooled analysis of data from 20 randomized clinical trials found a weak association between DpR and OS in mCRC. However, there were notable differences in tumor-size kinetics between antiangiogenic agents and anti-EGFR mAbs; the addition of anti-EGFR mAbs produced a larger treatment effect on the DpR^12^. Recently reported ARCAD studies have shown a strong correlation between tumor shrinkage and prognosis in combination chemotherapy with anti-EGFR mAb^13^. In our study, we were unable to show a correlation with OS in the *RAS* wild-type population, but we believe that it would have been difficult to show such a correlation in a patient group that included *BRAF* V600E mutation cases. The FIRE-4.5 study has demonstrated that survival time was shorter in m-FOLFOXIRI + cetuximab compared to m-FOLFOXIRI + bevacizumab as an initial treatment in *BRAF* V600E mutant mCRC^14^. In our study, the DpR correlated with PFS and OS in the *RAS*/*BRAF* wild-type and left-sided population. The DpR may be an important endpoint, as it may have potential as a surrogate for evaluating PFS and/or OS in clinical trials using regimens with anti-EGFR mAbs; however, the clinical use of DpR needs further validation.

Clinical trials comparing and validating bevacizumab with anti-EGFR mAbs have so far failed to consistently find a difference in PFS; in the PARADIGM trial, median PFS in patients with left-sided tumors treated with panitumumab plus m-FOLFOX6 was longer than patients treated with bevacizumab plus m-FOLFOX6 (13.1 months versus 11.9 months), but not statistically significant^2^. In this study, we demonstrated a statistically significant and clinically meaningful difference in PFS in patients with *RAS*/*BRAF* wild-type, left-sided disease, with a median PFS of 15.3 months versus 11.7 months (HR 0.68). Furthermore, to our knowledge, this is the first prospective study in mCRC to demonstrate a median OS exceeding 50 months. Such a favorable OS results can be attributed to patient selection for access to intensive treatment. However, the OS in the bevacizumab arm was comparable to the results of a sub-analysis of the TRIBE trial^15^, so it is not likely that the patients enrolled in this trial were in particularly good condition.

The clinical benefit of the triplet + anti-EGFR mAb regimen was not demonstrated in the TRIPLETE trial. Both ORR and DpR were not significantly different between m-FOLFOXIRI + panitumumab and m-FOLFOX6 + panitumumab. However, the DEEPER trial showed that the triplet + cetuximab was significantly more effective in reducing tumor size than the triplet + bevacizumab, although the control group was different. The relative dose intensities for anti-EGFR therapies were similar between TRIPLETE (81% and 75% for m-FOLFOX6 + panitumumab and m-FOLFOXIRI + panitumumab, respectively)^5^ and our study (77% for m-FOLFOXIRI + cetuximab). However, dosing frequency differed between the two studies, with cetuximab administered once weekly compared to biweekly with panitumumab. In this study, 83% of patients received a dose of cetuximab weekly for up to 8 cycles, which may have also led to favorable outcomes. The difference in dosing frequency may have resulted in skin toxicities being observed more frequently in this trial than in the TRIPLETE trial^5^.

This study had a number of limitations. Basically, the intent-to-treat (ITT) analysis should be performed on all randomized patients. However, we did not preplan the ITT analysis because the ITT cohort included patients for whom the DpR as primary endpoint could not be assessed. We defined the method of evaluation and analysis for the DpR according to the previous studies^8,16^. Therefore, the PPS for this study analysis excluded patients with no target lesions and patients in which new lesions appeared at the initial evaluation and patients in which only non-target lesions were progressive. As the level of missingness was low (4%), we conducted a complete case analysis (Supplementary Table 4). We believe that the ITT analysis cannot properly evaluate the DpR of the primary endpoint, but in the exploratory ITT analysis, the significant difference in the cetuximab arm remained. We should be cautious about using DpR as the primary endpoint in future clinical trials for mCRC. Second, the subgroup analysis according to *BRAF* V600E status was not pre-planned in this study. The DEEPER trial started in 2015, when *BRAF* testing was not available in clinical practice and clinical significance of *BRAF* V600E mutations was not clear. The primary tumor site information was proving to be clinically significant, so sidedness was considered as a stratification factor in the primary analysis. *BRAF* V600E data were available from electronic health records only for about 50% of patients. In addition, PFS and OS were analyzed without adjusting for multiplicity in *RAS*/*BRAF* wild-type patients. Therefore, the results of the analysis obtained in *RAS*/*BRAF* wild-type and left-sided patients may be hypothesis-generating. A biomarker study is on-going to perform genomic analysis using pre-treatment tissue samples and will confirm these findings in a cohort of more patients with known *BRAF* V600E status. Finally, when the DEEPER trial was initiated, FOLFOXIRI + bevacizumab was one of the standard first-line treatments. However, after the TRIPLETE and PARADIGM trials^2,5^, the standard first-line treatment for *RAS*/*BRAF* wild-type and left-sided mCRC is doublet + anti-EGFR mAb. There is some difficulty in evaluating this trial in the absence of a prospective trial examining doublet + anti-EGFR mAb versus FOLFOXIRI + bevacizumab. Our results may provide an opportunity to reconsider the significance of comparing triplet regimens with doublet + anti-EGFR mAb regimens.

In conclusion, our study demonstrated that m-FOLFOXIRI + cetuximab had clinical benefit for tumor shrinkage in mCRC patients with *RAS* wild-type tumors compared to m-FOLFOXIRI + bevacizumab, with manageable safety; however, survival time was comparable. In left-sided and *RAS*/*BRAF* wild-type tumors, triplet plus cetuximab may produce greater survival time, with longest median OS reported to date, of over 50 months.

## Methods

### Study design and participants

DEEPER (UMIN000018217, jRCTs061180022) was a multicenter, randomized, comparative, open-label, phase 2 trial comparing the efficacy and safety of m-FOLFOXIRI + cetuximab and m-FOLFOXIRI + bevacizumab as initial therapy in patients with unresectable *RAS* wild-type mCRC. Eligible participants were adults aged ≥20 years at the time of enrollment with a histologically confirmed diagnosis of *RAS* wild-type (*KRAS* exons 2, 3, and 4, and *NRAS* exons 2, 3, and 4), unresectable mCRC, and with a measurable lesion according to the Response Evaluation Criteria in Solid Tumors (RECIST) 1.1 and ECOG PS of 0 or 1 (patients aged ≥71 years were required to have a PS of 0). Full inclusion and exclusion criteria are available in the study protocol (Supplementary Note).

Written and informed consent was obtained from all participants before initiating trial screening procedures and enrollment. The trial was approved by a certified review board and conducted in compliance with the protocol and the Japanese Clinical Trials Act (Act No. 16 of 14 April 2017).

### Randomization and masking

Patients were randomly assigned (1:1) to receive m-FOLFOXIRI + cetuximab or m-FOLFOXIRI + bevacizumab by the minimization method using a centralized patient registration system. Randomization was performed centrally and stratified by primary tumor site (right or left), history of adjuvant chemotherapy (none, adjuvant chemotherapy including oxaliplatin, or not including oxaliplatin), and ECOG PS (0 or 1). The first patient was enrolled at 8^th^ September 2015 and the last patient was at 30^th^ June 2019.

### Procedures

For patients in the cetuximab arm, a 2-week course of the following treatments was administered, up to 12 cycles: cetuximab once weekly, with an initial dose of 400 mg/m^2^ as an intravenous (IV) infusion and subsequent doses (standard dose) of 250 mg/m^2^; irinotecan hydrochloride hydrate 150 mg/m^2^ as an IV infusion on Day 1; oxaliplatin 85 mg/m^2^ and levofolinate 200 mg/m^2^ as IV infusions at the same time on Day 1; and 5-FU 2400 mg/m^2^ as an IV infusion after the administration of oxaliplatin and levofolinate.

For patients in the bevacizumab arm, a 2-week course of the following treatments was administered, up to 12 cycles bevacizumab 5 mg/kg as an IV infusion on Day 1; irinotecan hydrochloride hydrate 150 mg/m^2^ as an IV infusion on Day 1; oxaliplatin 85 mg/m^2^ and levofolinate 200 mg/m^2^ as IV infusions at the same time on Day 1; and 5-FU 2400 mg/m^2^ as an IV infusion after the administration of oxaliplatin and levofolinate.

In either treatment arm, irinotecan dose was allowed to be reduced to 125 mg/m^2^ or 100 mg/m^2^ at the discretion of the treating physician for patients homozygous for UGT1A1*28 or UGT1A1*6, or heterozygous for both UGT1A1*28 and UGT1A1*6. When up to 12 cycles of m-FOLFOXIRI + bevacizumab or cetuximab were completed, m-FOLFOXIRI was switched to 5-FU + levofolinate. The maintenance therapy with 5-FU + levofolinate plus cetuximab or bevacizumab continued until disease progression, unacceptable AEs, or study withdrawal.

Granulocyte Colony Stimulating Factor (G-CSF) support was allowed only when grade 4 neutropenia or febrile neutropenia, but prophylactic use of G-CSF was not permitted.

### Outcomes

The primary endpoint of the study was depth of response (DpR), defined as the sum of the longest diameters of RECIST target lesions at the nadir in the absence of progression, subtracted from the sum of the longest diameters of target lesions at baseline and then divided by the sum of the longest diameters of target lesions at baseline in the per protocol set (PPS) consisting of patients evaluable for DpR, which was evaluated every 8 weeks until disease progression. Responses were evaluated according to the RECIST version 1.1 by the investigators and validated by an external review board. Secondary endpoints included ORR, early tumor shrinkage (ETS) at week 8, progression-free survival (PFS), overall survival (OS), time to tumor growth (TTG), time to treatment failure, R0 resection rate, and safety (incidence and severity of AEs). The association between DpR/TTG and PFS or OS was also evaluated as a secondary endpoint. Patients were followed up for 3 years after enrollment of the last patient registered.

### Statistical analysis

We choosed the DpR as the primary endpoint of this study for the following reasons: historical control data for PFS and OS of triplet plus cetuximab were not available at the time of planning this study; the OS of triplet plus bevacizumab was approximately 36 months long and the sample size was too large to test the OS superiority of triplet plus cetuximab; it has been known that in *RAS* wild-type mCRC, there was no difference in PFS between bevacizumab and anti-EGFR antibody combination regimens.

A sample size of 180 patients per group (360 patients in total) was planned, taking into account a 5% dropout rate, including withdrawals prior to administration. This was calculated to ensure 85% power to detect DpR superiority for m-FOLFOXIRI + cetuximab versus m-FOLFOXIRI + bevacizumab with a two-sided significance level of 0.05, assuming a difference in median DpR of 12.5% (standard deviation 42% and 34% for cetuximab and bevacizumab, respectively), based on findings from previous studies of anti-EGFR mAbs^5,8,9,17^.

The efficacy analysis was assessed in the PPS, comprising all patients who completed the prespecified minimum protocol treatment, had evaluable efficacy endpoint data for DpR, and had no major protocol violations. Safety was assessed in the safety population, comprising all patients who received at least one dose of the protocol treatment.

The primary endpoint was assessed using a t-test with Welch’s adjusted degree of freedom because of the expected differences in distribution between the two treatment groups. Secondary endpoints were compared with the chi-squared test for differences in ORR, ETS and resection rate, and log-rank test for PFS and OS. An exploratory subgroup analysis of the DpR by clinical factors was performed.

Data on *BRAF* V600E mutation status were retrospectively collected from the electronic health records of patients who underwent *BRAF* testing during routine clinical testing. A post-hoc exploratory analysis was performed for each endpoint according to *BRAF* status since treatment regimens are considered according to *RAS*/*BRAF* status in the standard of care for mCRC. Among patients with *BRAF* wild-type tumors, the cetuximab and bevacizumab groups were compared for patients with left-sided primary site. The sidedness was considered as a stratification factor in the primary analysis, however, *BRAF* status was not included. This subgroup analysis for *RAS*/*BRAF* wild-type and left-sided patients was a post-hoc analysis and repeated the same analysis performed on the PPS population for the DpR, PFS, and OS, so it did not take into account multiplicity, which should be done.

### Reporting summary

Further information on research design is available in the Nature Portfolio Reporting Summary linked to this article.

## Supplementary information

Supplementary Information

Reporting Summary

Peer Review File

## Source data

Source Data

## Data Availability

The study protocol is available as Supplementary Note in the Supplementary Information. The authors declare that all relevant data used to conduct the analyses are available within the article. To protect the privacy and confidentiality of patients in this study, clinical data are not made publicly available in a repository or the supplementary material of the article but can be requested at any time from the corresponding author Yu Sunakawa (y.sunakawa@marianna-u.ac.jp). Any requests will be reviewed within a time frame of 3 to 4 weeks by the JACCRO to verify whether the request is subject to any intellectual property or confidentiality obligations. The data will be made available for one month. All data shared will be de-identified. Source data are provided with this paper.
