# Supplementary material for: Modified FOLFOXIRI plus cetuximab versus bevacizumab in RAS wild-type metastatic colorectal cancer: a randomized phase II DEEPER trial
Source: Nat Commun. 2024 Nov 25;15:10217. doi: 10.1038/s41467-024-54460-2 (PMC11589592; doi:10.1038/s41467-024-54460-2)
Supplement: Supplementary file 1 — Supplementary Information [file 41467_2024_54460_MOESM1_ESM.pdf]

Supplementary information

Table of contents

Supplementary Figure 1. OS according to primary tumor in *RAS* wild-type patients of PPS; left-sided (A) and right-sided (B)……………………………………………..2

Supplementary Figure 2. PFS (A) and OS (B) in *RAS*/*BRAF* wild-type and right-sided patients of PPS……………………………………………………………………….4

Supplementary Figure 3. OS stratified by R0 resection in all patients (A), the m-FOLFOXIRI + cetuximab arm (B), and the m-FOLFOXIRI + bevacizumab arm (C)………..........6

Supplementary Figure 4. OS of bevacizumab arm vs. cetuximab arm according to R0 resection in *RAS*/*BRAF* wild-type and left-sided patients of PPS; patients with R0 resection and patients other than R0 resection……………………………………………………………………………… ……………………………………………….9

Supplementary Table 1. Median depth of response according to clinical factors in *RAS* wild-type patients…………………………………………………...………….11

Supplementary Table 2. Treatment response by *RAS*/*BRAF* status and tumor sidedness……………………………………………………...…………………………….12

Supplementary Table 3. Clinical outcomes in patients with right-sided tumors ……………………………………………………………………………...……….13

Supplementary Table 4. Exploratory analysis of depth of response (DpR) for *RAS* wild-type patients in ITT population and PPS population…………………...……….14

Supplementary Note: Protocol

Supplementary Figure 1: OS according to primary tumor in *RAS* wild-type patients of PPS; left-sided (A) and right-sided (B). A log-rank test was used with a two-sided significance.

A

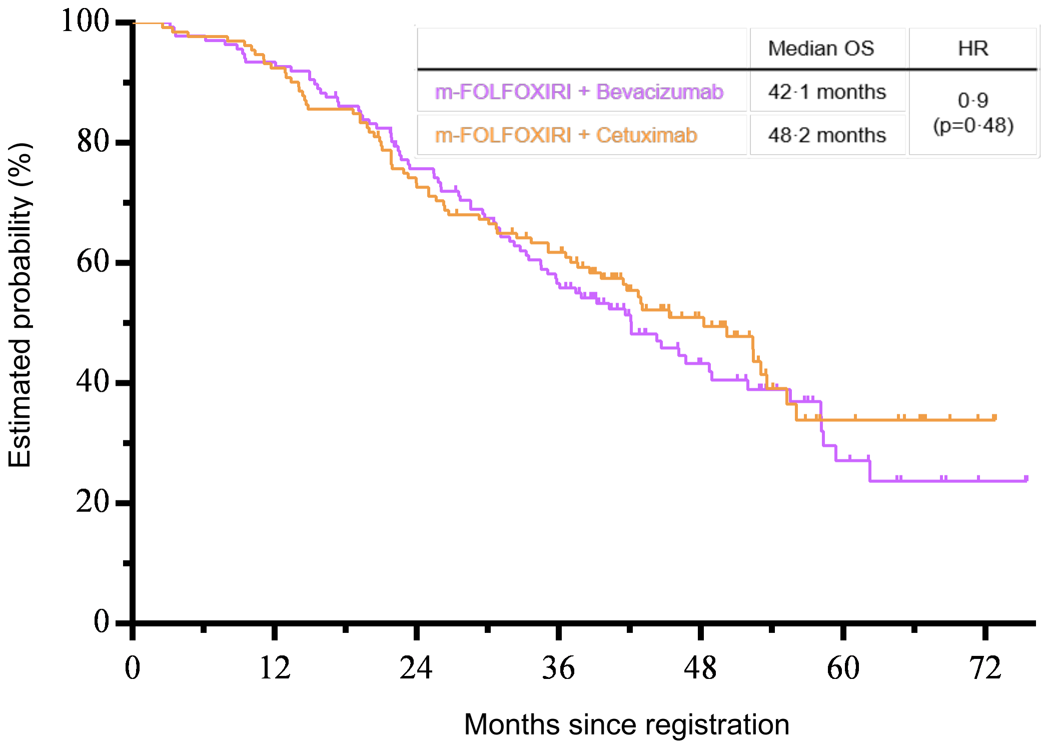

Number at risk

m-FOLFOXIRI + bevacizumab	137	134	128	117	101	89	73	49	32	21	10	5	2
m-FOLFOXIRI + cetuximab	132	129	122	113	95	87	77	53	34	17	10	7	2

HR=hazard ratio. OS=overall survival.

B

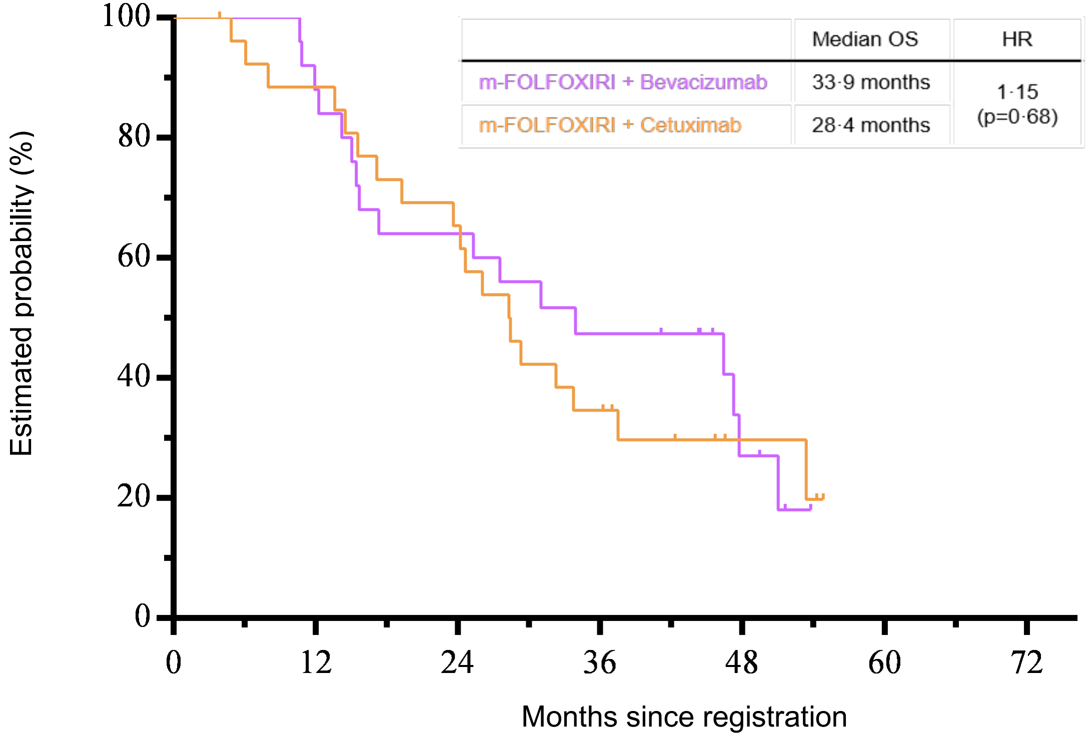

Number at risk

m-FOLFOXIRI + bevacizumab	25	25	22	16	16	13	11	10	4	
m-FOLFOXIRI + cetuximab	27	25	23	19	17	11	9	6	3	2

HR=hazard ratio. OS=overall survival.

Supplementary Figure 2: PFS (A) and OS (B) in *RAS*/*BRAF* wild-type and right-sided patients of PPS. A log-rank test was used with a two-sided significance.

A

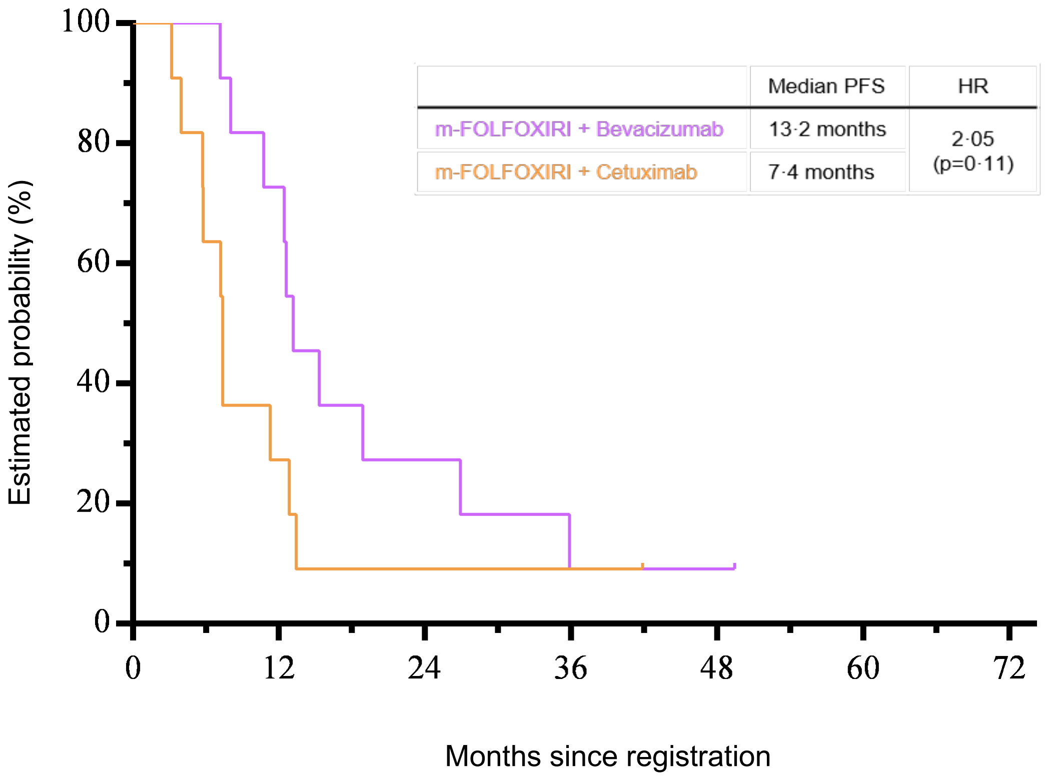

Number at risk

m-FOLFOXIRI + bevacizumab	11	11	8	4	3	2	1	1	1
m-FOLFOXIRI + cetuximab	11	7	3	1	1	1	1		

HR=hazard ratio. PFS=progression free survival.

B

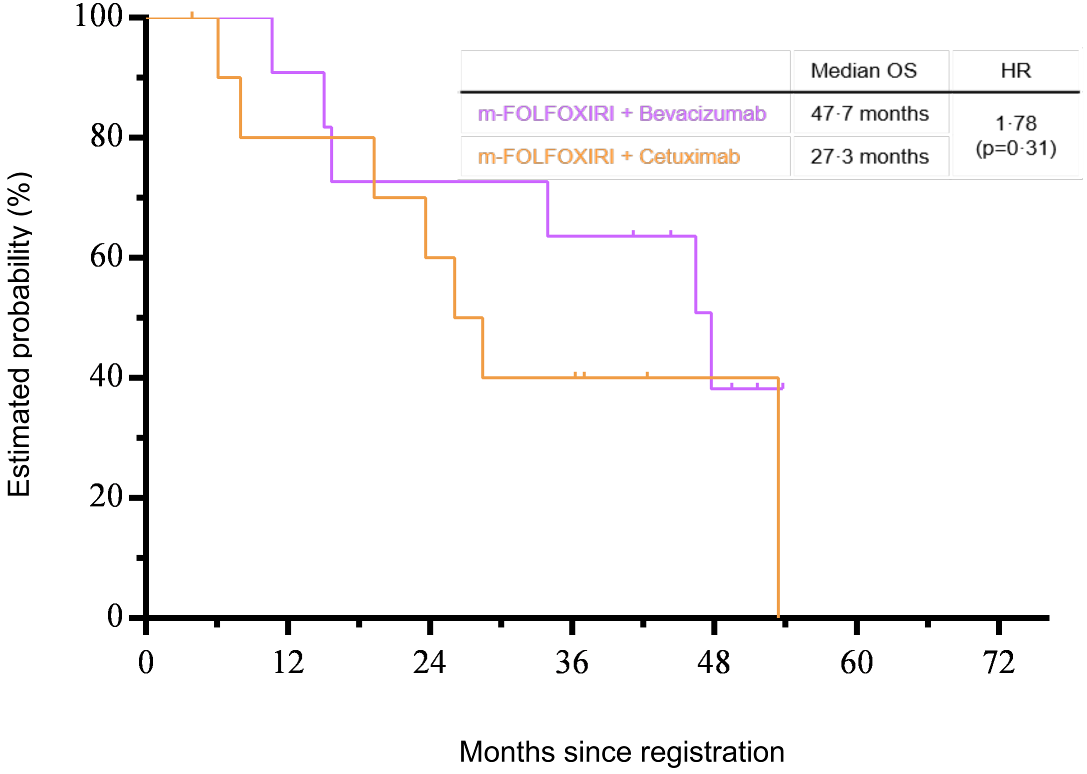

Number at risk

m-FOLFOXIRI + bevacizumab	11	11	10	8	8	8	7	6	3
m-FOLFOXIRI + cetuximab	11	10	8	8	6	4	4	2	1

HR=hazard ratio. OS=overall survival.

Supplementary Figure 3. OS stratified by R0 resection in all patients (A), the m-FOLFOXIRI + cetuximab arm (B), and the m-FOLFOXIRI + bevacizumab arm (C). A log-rank test was used with a two-sided significance.

A

	Median OS	HR (95%CI)
R0 resection	Not Reached	0·14 (0.079-0.24) (p<0·0001)
Non-R0 resection	32·3 months	

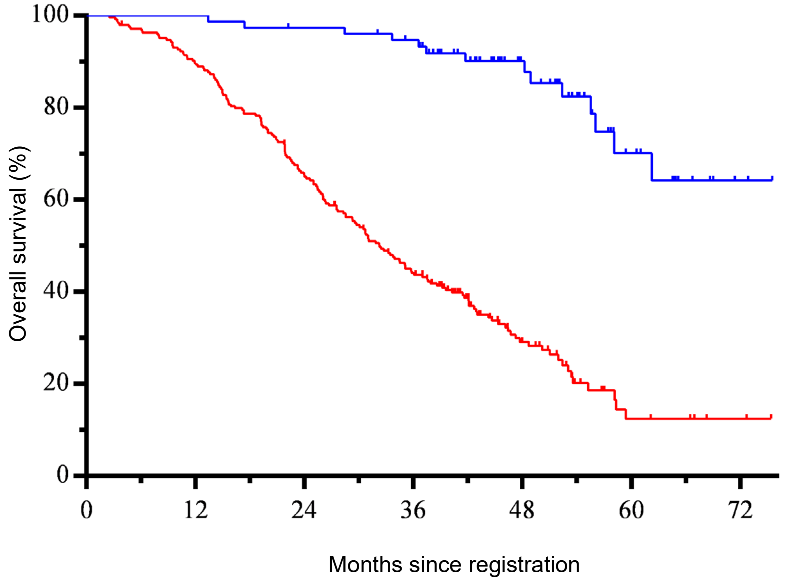

Number at risk													
R0 resection	76	76	76	74	73	72	69	53	38	26	14	7	2
Non-R0 resection	245	237	219	191	156	128	101	65	35	14	6	5	2

CI=confidence interval. HR=hazard ratio. OS=overall survival.

B

	Median OS	HR (95% CI)
R0 resection	Not Reached	0·15 (0.07-0.30) (p<0·0001)
Non-R0 resection	30·7 months	

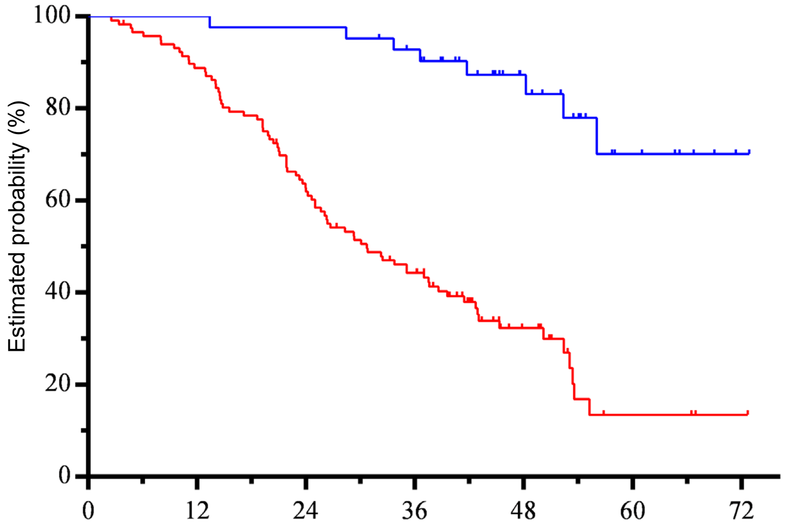

Number at risk

R0 resection	42	42	42	41	41	40	37	29	21	14	7	4	1
Non-R0 resection	117	112	103	91	71	58	49	30	16	5	3	3	1

CI=confidence interval. HR=hazard ratio. OS=overall survival.

C

	Median OS	HR (95% CI)
R0 resection	Not Reached	0·13 (0.054-0.29) (p<0·0001)
Non-R0 resection	33·2 months	

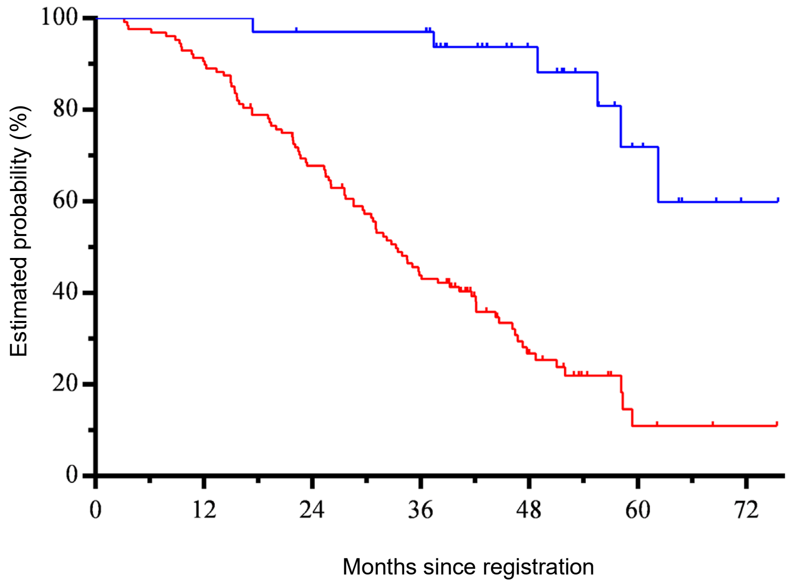

Number at risk												
R0 resection	34	34	34	33	32	32	32	24	17	12	7	3	1
Non-R0 resection	128	125	116	100	85	70	52	35	19	9	3	2	1

CI=confidence interval. HR=hazard ratio. OS=overall survival.

Supplementary Figure 4: OS of bevacizumab arm vs. cetuximab arm according to R0 resection in *RAS/BRAF* wild-type and left-sided patients of PPS; patients with R0 resection and patients other than R0 resection. A log-rank test was used with a two-sided significance.

A

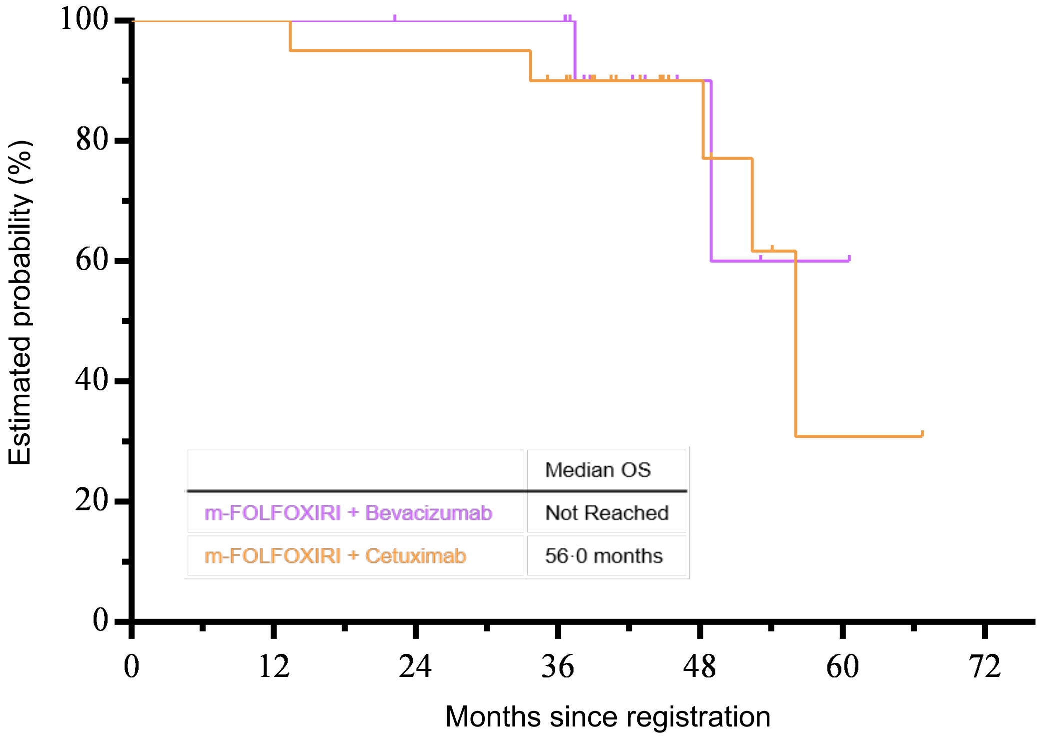

Number at risk											
m-FOLFOXIRI + bevacizumab	14	14	14	14	13	13	13	6	3	1	1	
m-FOLFOXIRI + cetuximab	20	20	20	19	19	19	17	11	7	4	1	1

HR=hazard ratio. OS=overall survival.

B

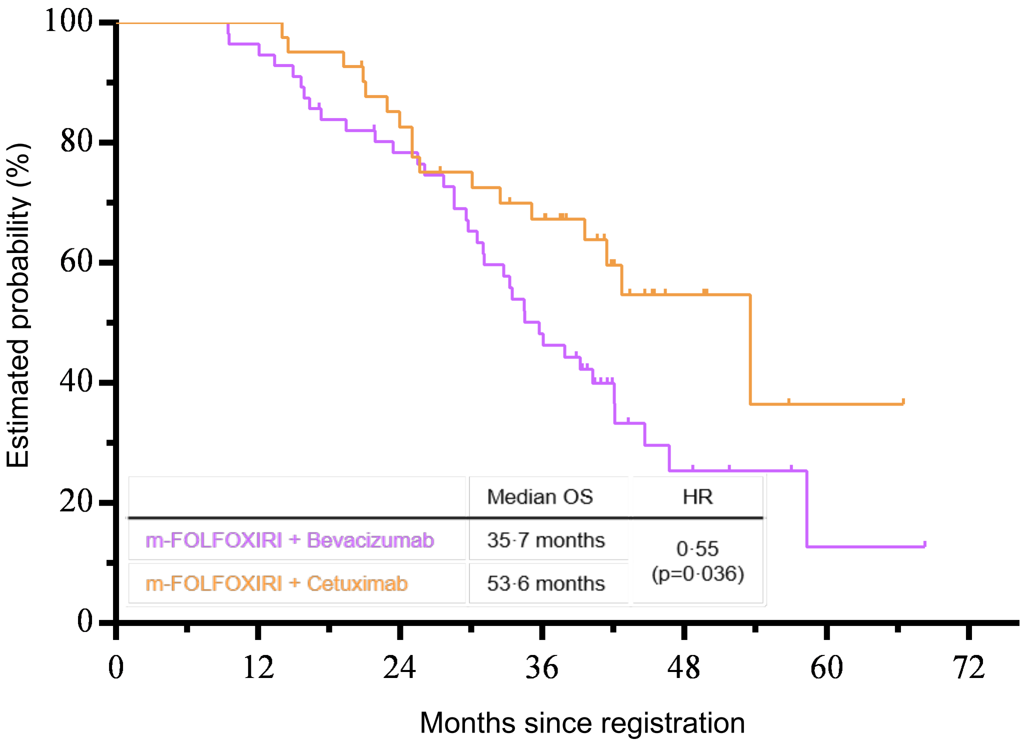

Number at risk												
m-FOLFOXIRI + bevacizumab	56	56	54	46	42	35	25	12	6	3	1	1
m-FOLFOXIRI + cetuximab	41	41	41	39	33	29	25	13	5	2	1	1

HR=hazard ratio. OS=overall survival.

Supplementary Table 1: Median depth of response according to clinical factors in *RAS* wild-type patients

	m-FOLFOXIRI + bevacizumab	m-FOLFOXIRI + cetuximab	p-value*
**Sex**			
Male	47·0%	60·5%	p<·0001
Female	43·6%	49·1%	p=0·43
**ECOG PS**			
0	46·9%	55·7%	p=0·0051
1	40·6%	68·2%	p=0·30
**Site of primary tumor**			
Left	46·1%	59·2%	p=0·0026
Right	41·2%	50·0%	p=0·47
**Resected primary tumor**			
Yes	48·0%	60·4%	p=0·014
No	44·0%	53·3%	p=0·083
**Time to metastases**			
Synchronous	44·7%	59·2%	p=0·0004
Metachronous	52·4%	50·0%	p=0·62
**Number of metastatic sites**			
0-1	49.3%	60.3%	p=0.046
>1	42.9%	56.4%	p=0.022
**Liver-only disease**			
Yes	51.9%	64.2%	p=0.0055
No	43.5%	53.5%	p=0.043
***BRAF* V600E status**			
Wild-type	47·9%	60·4%	p=0·0070
Mutant	38·8%	50·2%	p=0·99

ECOG PS=Eastern Cooperative Oncology Group Performance Status.

* chi-squared test

Supplementary Table 2: Treatment response and survival time by *RAS*/*BRAF* status and tumor sidedness

	**m-FOLFOXIRI + cetuximab**	**m-FOLFOXIRI + bevacizumab**	HR (95% CI), p-value
RAS wild-type (n=321)	n=159	n=162	
Median DpR	57·3%	46·0%	p=0·0029*
ORR	71·1%	69·1%	p=0·71**
Median PFS	13·0 months	12·3 months	HR 0·89 (0·70–1·12), p=0·32***
Median OS	42·9 months	42·1 months	HR 0·94 (0·71–1·26), p=0·68***
RAS wild-type, left-sided (n=269)	n=132	n=137	
Median DpR	59·2%	46·1%	p=0·0026*
ORR	74·2%	70·8%	p=0·53**
Median PFS	13·8 months	12·1 months	HR 0·80 (0·62–1·04), p=0·09***
Median OS	48·2 months	42·1 months	HR 0·89 (0·65–1·23), p=0·48***
RAS/*BRAF* wild-type (n=153)	n=72	n=81	
Median DpR	60·4%	47·9%	p=0·0070*
ORR	79·2%	72·8%	p=0·36**
Median PFS	13·8 months	12·3 months	HR 0·80 (0·57–1·12), p=0·19****
Median OS	53·4 months	42·1 months	HR 0·67 (0·42–1·07), p=0·091***
RAS/*BRAF* wild-type, left-sided (n=131)	n=61	n=70	
Median DpR	63·6%	47·8%	p=0·0003*
ORR	83·6%	72·9%	p=0·14**
Median PFS	15·3 months	11·7 months	HR 0·68 (0·47–0·98), p=0·036***
Median OS	53·6 months	40·2 months	HR 0·54 (0·32–0·91), p=0·020***

CI=confidence interval. DpR=depth of response. m-FOLFOXIRI= modified 5-FU, leucovorin, oxaliplatin and irinotecan. ORR=objective response rate. PFS=progression-free survival. OS=overall survival

* chi-squared test, ** t-test with Welch's adjusted degree of freedom, ***log-rank test with a two-sided significance

Supplementary Table 3. Clinical outcomes in patients with right-sided tumors

	**m-FOLFOXIRI + bevacizumab**	**m-FOLFOXIRI + cetuximab**	HR (95%CI), p-value
RAS wild-type, right-sided (n=52)	n=25	n=27	
Median DpR	41·2 %	50·0 %	p=0·47*
ORR	60·0%	55·6%	p=0·75**
Median PFS	12·8 months	9·0 months	HR 1·54 (0·87-2·72), p=0·14***
Median OS	33·9 months	28·4 months	HR 1·15 (0·59-2·22), p=0·68***
RAS/*BRAF* wild-type, right-sided (n=22)	n=11	n=11	
Median DpR	50·0 %	44.7 %	p=0·45*
ORR	72·7%	54·5%	p=0·38**
Median PFS	13·2 months	7·4 months	HR 2·05 (0·84-5·01), p=0·11***
Median OS	47·7 months	27·3 months	HR 1·78 (0·58-5·46), p=0·31***

DpR=depth of response. ORR=objective response rate. PFS=progression free survival. OS=overall survival

* chi-squared test, ** t-test with Welch's adjusted degree of freedom, ***log-rank test with a two-sided significance

Supplementary Table 4: Exploratory analysis of depth of response (DpR) for *RAS* wild-type patients in ITT population and PPS population

	**m-FOLFOXIRI + cetuximab**	**m-FOLFOXIRI + bevacizumab**
ITT population		
Total number of patients (n)	179	180
Patients excluded from ITT analysis (n)	12	11
Reasons for the exclusion (patients with no data of DpR)		
No administration (no protocol treatment)	4*	4
No target lesions	0	3
Not evaluated during protocol treatment	7	4
Not evaluated in a prescribed manner	1	0
**Patients evaluable for ITT analysis**	**167**	**169**
Median DpR (range)	55·9% (-120.2–100)	46·0% (-0.50.1–100)
Mean (95% CI)	52·3% (47·6-57·1)	46·3% (43·0–49·7)
Standard deviation (95% CI)	31·10 (28·1-34·9)	22·29 (20·1–25·0)
Welch's t-test	p=0·044	
⇩		
Patients who do not meet the definition of DpR analysis	8	7
Reasons for not evaluable for DpR		
Could not be evaluated by external review board due to lack of submission of image data	2	5
Progression of non-target lesions detection of new lesions	6	2
⇩		
PPS population		
**Patients evaluable for PPS analysis**	**159**	**162**
Median DpR (range)	57·3% (-42·6–100)	46·0% (-0.6–100)
Mean (95% CI)	55·2% (51·1–59·3)	47·3% (44·1–50·5)
Standard deviation (95% CI)	26·17 (23·6–29·4)	20·53 (18·5–23·0)
Welch's t-test	p=0·0029	

*1 duplicate registration

Supplementary Note

JACCRO

A randomized phase II study to investigate the deepness of response of FOLFOXIRI plus cetuximab (Erbitux) versus FOLFOXIRI plus bevacizumab as the first-line therapy in metastatic colorectal cancer patients with *RAS* wild-type tumors: DEEPER

JACCRO CC-13
PROTOCOL

[redacted]

Ver. 1.6: July 30, 2021

Japan Clinical Cancer Research Organization

0 Synopsis

0.1 Research Title

A randomized phase II study to investigate the deepness of response of FOLFOXIRI plus cetuximab (Erbitux) versus FOLFOXIRI plus bevacizumab as the first-line therapy in metastatic colorectal cancer patients with *RAS* wild-type tumors

0.2 Type of Study

A multicenter, randomized, comparative phase II study (open-label) using the central registration method

0.3 Study Objective

To demonstrate the superiority of FOLFOXIRI plus cetuximab over FOLFOXIRI plus bevacizumab in patients with *RAS* wild-type, unresectable metastatic/recurrent colorectal cancer

0.4 Subjects

Patients with *RAS* wild-type (*KRAS* exon 2, 3, 4, *NRAS* exon 2, 3, 4), unresectable metastatic/recurrent colorectal cancer with a measurable lesion according to RECIST criteria (Ver.1.1)

0.5 Subject Selection

0.5.1 Eligibility Criteria

Patients meeting all of the following criteria are eligible for this study. Gender is not a criterion.

(1) Patients with histologically confirmed colorectal cancer

(2) Patients with *RAS* wild-type, unresectable metastatic/recurrent colorectal cancer

(3) Patients with a measurable lesion according to RECIST criteria (Ver.1.1)

(4) Patients with unresectable primary tumors or unresectable distant or lymph node metastases who have not received any prior chemotherapy (patients undergoing surgery must have not received any treatment other than surgery)

Patients with first recurrence after surgery for primary tumors or metastases who have not received any treatment for the recurrence including surgery (patients undergoing postoperative adjuvant therapy must have experienced relapse more than 12 months after completion of the therapy)

*See Figure 0.5.1.1 “Eligible Patients with Unresectable Primary Tumors or Unresectable Distant or Lymph Node Metastases” and Figure 0.5.1.2 “Eligible Patients with First Recurrence”

(5) Patients aged 20 years or older at the time of informed consent

(6) Patients with ECOG performance status (PS) of 0 to 1; patients aged 71 years or older must have a PS of 0

(7) Patients with an estimated life expectancy of 6 months or more

(8) Patients who meet all of the following laboratory criteria for major organ functions within 14 days of enrollment

If there are multiple laboratory data during the period, the most recent data before enrollment

should be used. If laboratory data are measured newly, no blood transfusion or administration of hematopoietic factor preparations is permitted within 14 days of the measurement.

1) White blood cell count ≥ 3,000/mm^3 and < 12,000/mm^3
2) Neutrophil count ≥ 1,500/mm^3
3) Platelet count ≥ 10.0×10^4/mm^3
4) Hemoglobin ≥ 9.0 g/dL
5) Blood bilirubin ≤ 1.5 times the upper limit of the institutional normal range
6) AST ≤ 2.5 times the upper limit of the institutional normal range (≤ 5 times the upper limit of the institutional normal range if liver metastasis is present)
7) ALT ≤ 2.5 times the upper limit of the institutional normal range (≤ 5 times the upper limit of the institutional normal range if liver metastasis is present)
8) Serum creatinine ≤ 1.5 times the upper limit of the institutional normal range
9) Urine protein ≤ 1+
10) PT-INR ≤ 1.5

(9) Patients who have provided written consent after being fully informed of the contents of the present study

<Eligible patients with unresectable primary tumors or unresectable distant or lymph node metastases>

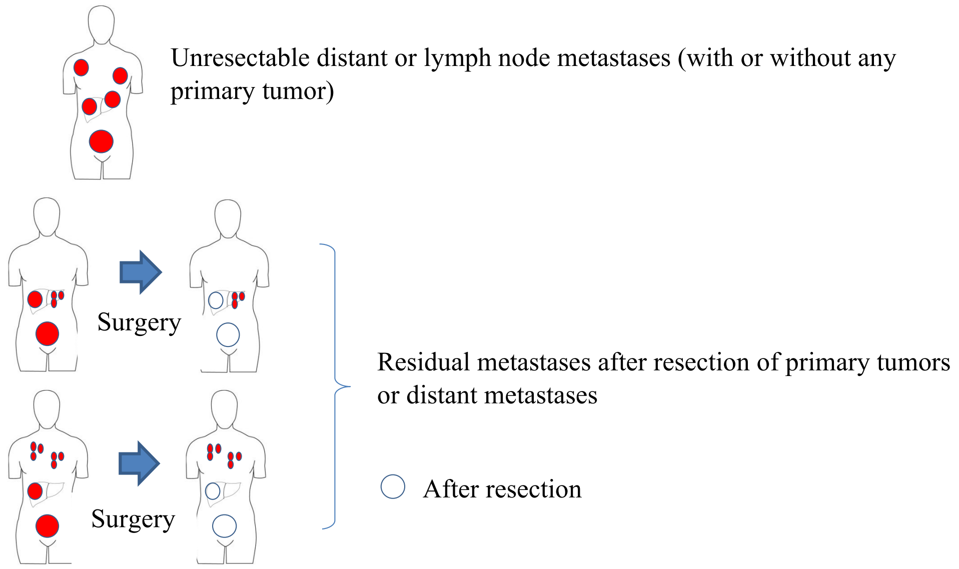

Figure 0.5.1.1 Eligible Patients with Unresectable Primary Tumors or Unresectable Distant or Lymph Node Metastases

<Patients with recurrence>

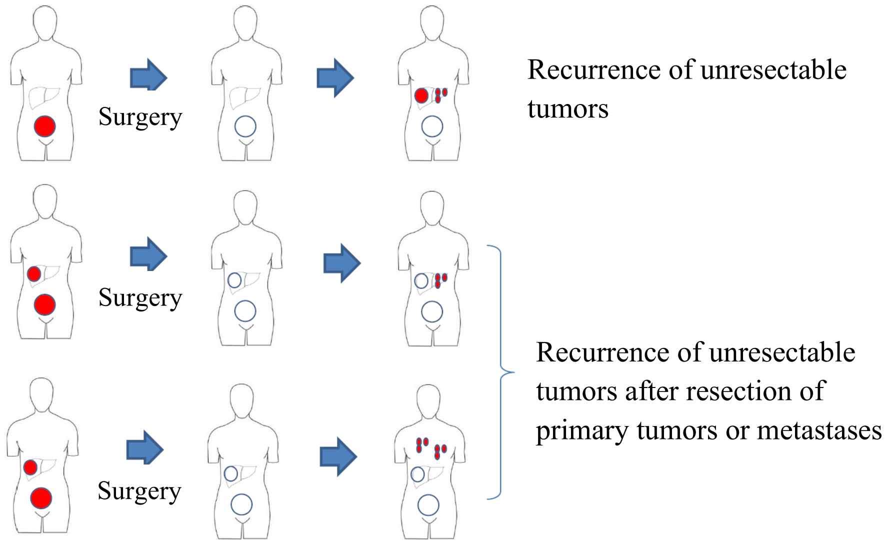

Figure 0.5.1.2 Eligible Patients with First Recurrence

0.5.2 Exclusion Criteria

Patients meeting any of the following criteria are excluded from this study.

(1) Patients with synchronous double cancers or metachronous double cancers with a disease-free interval less than 5 years (not excluding any early-stage cancer expected to be cured by topical therapy)

(2) Patients with suspected hereditary colorectal cancer for their family history or other reasons

(3) Patients with brain metastasis

(4) Patients with infectious disease

(5) Patients with interstitial pneumonia or pulmonary fibrosis

(6) Patients with concurrent or previous serious heart disease

(7) Patients with a history of thromboembolism

(8) Patients with cerebrovascular disease

(9) Patients with a history of hematemesis or hemoptysis

(10) Patients with poorly controlled hypertension

(11) Patients with severe paresthesia or dysesthesia accompanied by functional impairment

(12) Patients with a large amount of malignant fluid (pleural, abdominal, or pericardial effusion)

(13) Patients with serious comorbidities (e.g., renal failure, hepatic failure, hypertension)

(14) Patients who have undergone radiation therapy for primary tumors or metastases

(15) Men who are not willing to practice contraception, or pregnant women, lactating women, women with a positive pregnancy test, or women who are not willing to practice contraception

(16) Patients with a history of serious hypersensitivity

(17) Patients with a positive test for HBsAg or with active viral hepatitis

(18) Patients who have received blood transfusion, blood products, or hematopoietic factor preparations

such as G-CSF within 14 days of enrollment

(19) Patients who have undergone surgical therapy, biopsy with skin incision, or surgical closure of trauma within 28 days of enrollment, or patients who have undergone fine-needle aspiration biopsy (excluding central venous port implantation) within 7 days of enrollment

(20) Patients who will require or currently receive any drug that suppresses platelet function (aspirin preparations at a daily dose of 325 mg or more or nonsteroidal anti-inflammatory drugs) for chronic inflammatory diseases such as rheumatoid arthritis

(21) Patients with a bleeding diathesis (including hemoptysis, or cavitation and/or necrosis of pulmonary metastases found by imaging studies), coagulopathy, or abnormalities in coagulation factors

(22) Patients with gastrointestinal tract perforation or a history of gastrointestinal tract perforation within 1 year of enrollment

(23) Patients with unhealed traumatic fracture

(24) Patients with watery stool or uncontrollable diarrhea

(25) Patients who have undergone organ transplantation requiring immunosuppressive drugs

(26) Patients who have been treated with cetuximab, bevacizumab, oxaliplatin, or irinotecan (excluding oxaliplatin used as postoperative adjuvant chemotherapy)

(27) Patients currently receiving atazanavir sulfate (REYATAZ)

(28) Patients with jaundice

(29) Patients with intestinal paresis or obstruction

(30) Any others considered to be ineligible for study participation by the investigator or subinvestigator

0.6 Design

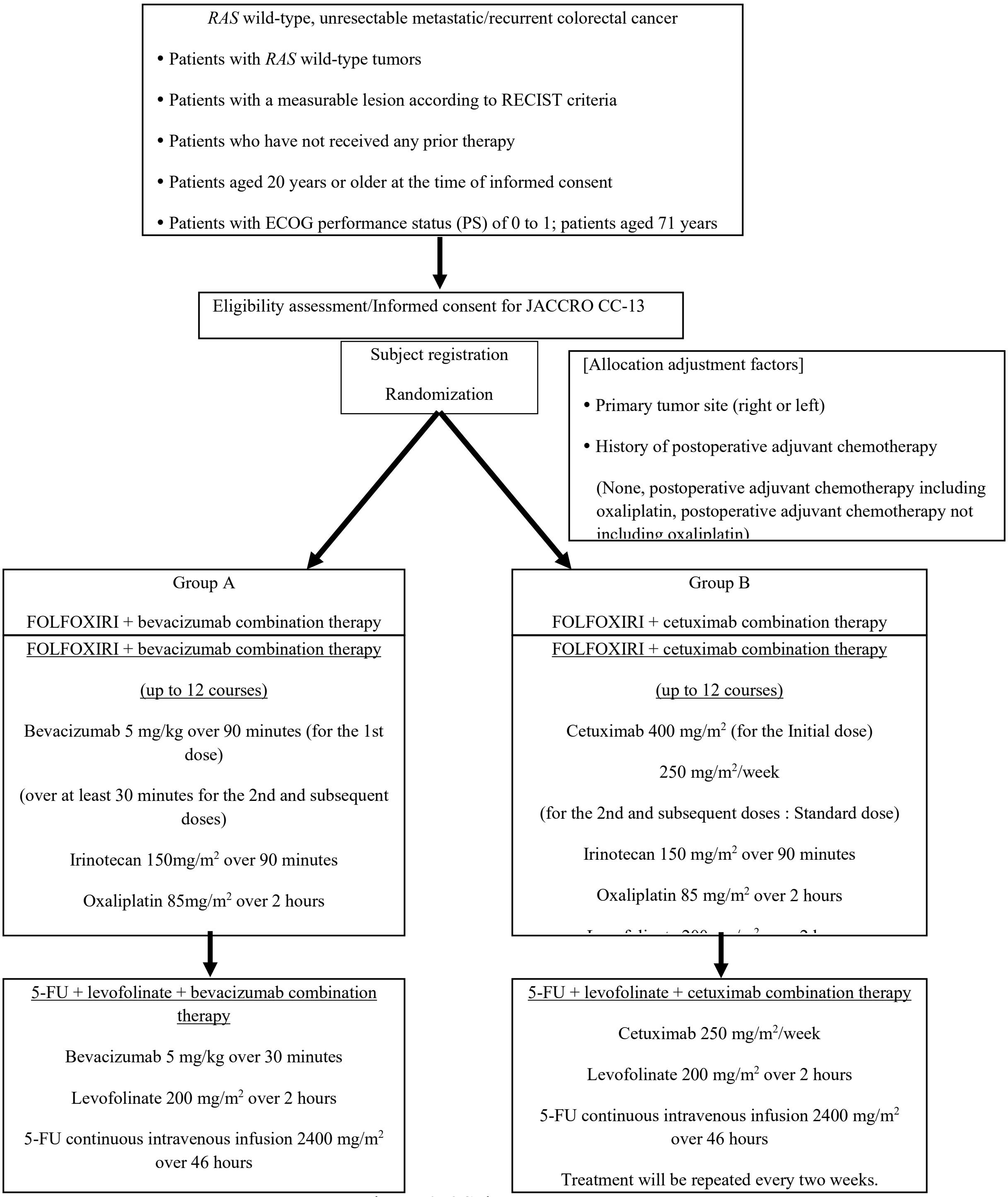

Figure 0.6 Scheme

0.7 Protocol Treatment

0.7.1 Treatment Drugs, Dose, and Administration Method

0.7.1.1 Group A: FOLFOXIRI + Bevacizumab Combination Therapy (up to 12 courses)

A 2-week course consists of the following treatments and each subject will receive up to 12 courses of therapy:

(1) Bevacizumab: Bevacizumab 5 mg/kg will be administered as an intravenous infusion over 90 minutes on Day 1 for the 1st dose. If the 1st dose is well tolerated, the 2nd dose may be administered over 60 minutes. If the 2nd dose is also well tolerated, subsequent doses may be administered over 30 minutes.

(2) Irinotecan: Irinotecan hydrochloride hydrate 150 mg/m^2 will be administered as an intravenous infusion over 90 minutes on Day 1.

(3) Oxaliplatin and levofolinate: Oxaliplatin 85 mg/m^2 and levofolinate 200 mg/m^2 will be administered as intravenous infusions at the same time over 2 hours on Day 1.

(4) 5-FU: After completion of the administration of oxaliplatin and levofolinate, 5-FU 2400 mg/m^2 will be administered as a continuous intravenous infusion over 46 hours.

►For patients homozygous for *UGT1A1*28* or *UGT1A1*6* or patients heterozygous for both *UGT1A1*28* and *UGT1A1*6*, irinotecan dose may be reduced to 125 mg/m^2 or 100 mg/m^2 at the discretion of the primary physician.

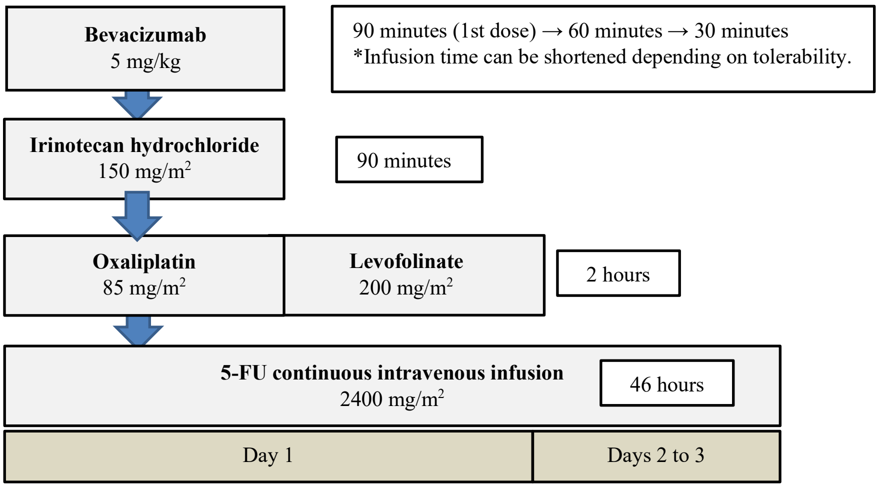

Figure 0.7.1.1 FOLFOXIRI + Bevacizumab Combination Therapy

FOLFOXIRI + bevacizumab combination therapy will be switched to 5-FU + levofolinate + bevacizumab combination therapy if either of the following is met:

- Up to 12 courses of FOLFOXIRI + bevacizumab combination therapy have been completed.
- A further dose reduction is needed after 2 dose-level reductions of irinotecan and oxaliplatin.

5-FU + Levofolinate + Bevacizumab Combination Therapy

(1) Bevacizumab: Bevacizumab 5 mg/kg will be administered as an intravenous infusion over 30 minutes.

(2) Levofolinate: Levofolinate 200 mg/m^2 will be administered as an intravenous infusion over 2 hours.

(3) 5-FU: After completion of the administration of levofolinate, 5-FU 2400 mg/m^2 will be administered as a continuous intravenous infusion over 46 hours. If any dose reduction of 5-FU has been made during FOLFOXIRI + bevacizumab combination therapy, the same dose will be continued.

0.7.1.2 Group B: FOLFOXIRI + Cetuximab Combination Therapy (up to 12 courses)

A 2-week course consists of the following treatments and each subject will receive up to 12 courses of therapy.

(1) Cetuximab:

Before administration of cetuximab, premedication specified at each center will be given for the prevention of infusion reactions to cetuximab and nausea/vomiting induced by irinotecan.

(e.g.)

- H1 antagonists (e.g., oral administration of diphenhydramine hydrochloride preparation 50 mg)
- 5-HT3 receptor antagonists (e.g., intravenous infusion of granisetron hydrochloride 3 mg)
- Corticosteroids (e.g., intravenous infusion of dexamethasone sodium phosphate 8 mg)

Cetuximab will be administered once weekly. For the Initial dose on Day 1, 400 mg/m^2 of cetuximab dissolved in 500 mL of isotonic sodium chloride solution will be administered as an intravenous infusion at an infusion rate of ≤5 mg/min over at least 120 minutes. For the 2nd and subsequent doses (Standard dose) , 250 mg/m^2 of cetuximab dissolved in 250 mL of isotonic sodium chloride solution will be administered as an intravenous infusion at an infusion rate of ≤10 mg/min over at least 60 minutes. Doses should be separated by at least 1 week.

If any grade 1 infusion reaction occurs during the infusion, the infusion rate should be further reduced (e.g., the infusion rate should be reduced by 50%: from ≤10 mg/min to ≤5 mg/min).

If any grade 2 infusion reaction occurs during the infusion, the subject should be observed while infusion is being interrupted. Then, infusion should be continued with care at a further reduced infusion rate (e.g., the infusion rate should be reduced by 50%: from ≤10 mg/min to ≤5 mg/min). If any infusion reaction occurs again after infusion is resumed at a further reduced infusion rate, administration of cetuximab should be discontinued.

If any grade 3 infusion reaction occurs, administration should be discontinued immediately and should not be repeated.

(2) Irinotecan: Irinotecan hydrochloride hydrate 150 mg/m^2 will be administered as an intravenous infusion over 90 minutes on Day 1.

(3) Oxaliplatin 85 mg/m^2 and levofolinate 200 mg/m^2 will be administered as intravenous infusions at

the same time over 2 hours on Day 1.

(4) 5-FU: After completion of the administration of oxaliplatin and levofolinate, 5-FU 2400 mg/m^2 will be administered as a continuous intravenous infusion over 46 hours.

►For patients homozygous for *UGT1A1*28* or *UGT1A1*6* or patients heterozygous for both *UGT1A1*28* and *UGT1A1*6*, irinotecan dose may be reduced to 125 mg/m^2 or 100 mg/m^2 at the discretion of the primary physician.

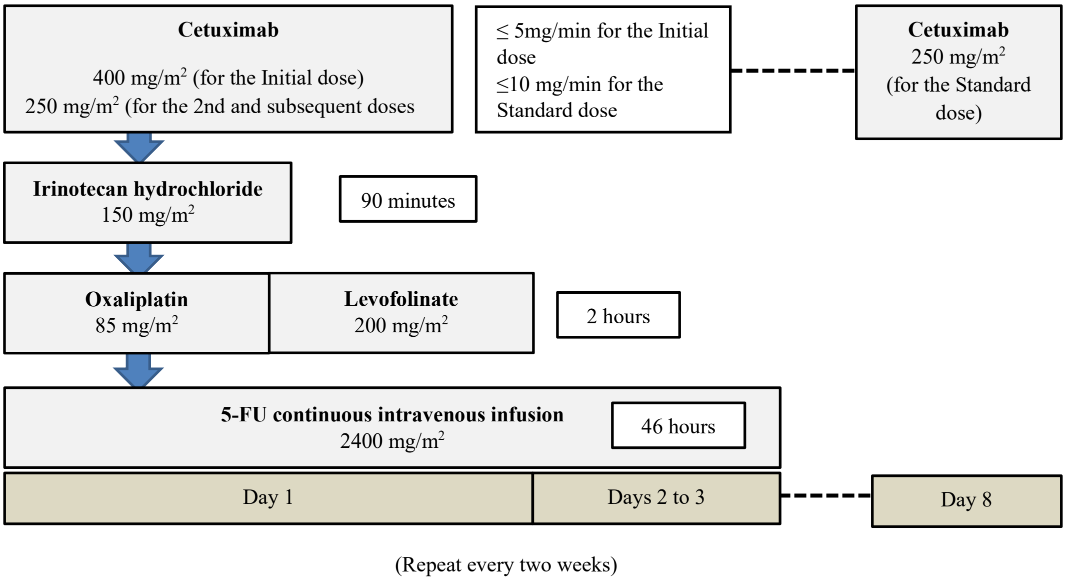

Figure 0.7.1.2 FOLFOXIRI + Cetuximab Combination Therapy

FOLFOXIRI + cetuximab combination therapy will be switched to 5-FU + levofolinate + cetuximab combination therapy if either of the following is met:

- Up to 12 courses of FOLFOXIRI + cetuximab combination therapy have been completed.
- A further dose reduction is needed after 2 dose-level reductions of irinotecan and oxaliplatin.

5-FU + Levofolinate + Cetuximab Combination Therapy

(1) Cetuximab: Cetuximab will be administered once weekly, and 250 mg/m^2 of cetuximab dissolved in 250 mL of isotonic sodium chloride solution will be administered as an intravenous infusion at an infusion rate of ≤10 mg/min over at least 60 minutes. Doses should be separated by at least 1 week.

If any grade 1 infusion reaction occurs during the infusion, the infusion rate should be further reduced (e.g., the infusion rate should be reduced by 50%: from ≤10 mg/min to ≤5 mg/min).

If any grade 2 infusion reaction occurs during the infusion, the subject should be observed while infusion is being interrupted. Then, infusion should be continued with care at a further reduced infusion rate (e.g., the infusion rate should be reduced by 50%: from ≤10 mg/min to ≤5 mg/min).

If any infusion reaction occurs again after infusion is resumed at a further reduced infusion rate, administration of cetuximab should be discontinued.

If any grade 3 infusion reaction occurs, administration should be discontinued immediately and should not be repeated.

(2) Levofolinate: Levofolinate 200 mg/m^2 will be administered as an intravenous infusion over 2 hours.

(3) 5-FU: After completion of the administration of levofolinate, 5-FU 2400 mg/m^2 will be administered as a continuous intravenous infusion over 46 hours. If any dose reduction of 5-FU has been made during FOLFOXIRI + cetuximab combination therapy, the same dose will be continued.

0.8 Endpoints

0.8.1 Primary Endpoint

Deepness of response (DpR)*

*Deepness of response (DpR) is defined as the sum of the longest diameters of RECIST target lesions at the nadir in the absence of progression subtracted from the sum of the longest diameters of RECIST target lesions at baseline divided by the sum of the longest diameters of RECIST target lesions at baseline.

0.8.2 Secondary Endpoints

Early tumor shrinkage (ETS) at Week 8

Response rate (RR)

Deepness of response (DpR) by Month 4

Time to treatment failure (TTF)

Time to tumor growth (TTG)

Progression-free survival (PFS)

Overall survival (OS)

Association between tumor shrinkage (ETS, RR, DpR) and prognosis (PFS, OS)

Association between TTG and prognosis (OS)

Resection rate

R0 resection rate

Safety (incidence and severity of adverse events)

0.9 Target Sample Size

180 subjects per group, 360 subjects in total

0.10 Study Period

Enrollment period: July 2015 to June 2019 (for 4 years)

Follow-up period: For 3 years after registration of the last subject

Study period: July 2015 to June 2022 (for 7 years)

0.11 Study Implementation System

(1) Principal Investigator

[redacted]

[redacted]

Kouhei Akazawa Department of Medical Informatics, Niigata University Medical and Dental Hospital

(6) Participating Institutions and Investigators

See Attachment.

(7) JACCRO Study Secretariat

Nonprofit Organization Japan Clinical Cancer Research Organization (JACCRO)

6F Jinbocho Kyowa Building, 1-64-3 Kandajinbocho, Chiyoda-ku, Tokyo 101-0051, Japan

Phone: +81-3-6811-0433 Fax: +81-3-6811-0434

E-mail: cc13.dc@jaccro.or.jp

(8) JACCRO CC-13 Data Center

EPS Corporation

2F Acropolis Tokyo, 6-29 Shinogawamachi, Shinjuku-ku, Tokyo 162-0814, Japan

Phone: +81-3-6304-5497 Fax: +81-3-6304-5496

E-mail: prj-jaccrocc13-dc@eps.co.jp

(9) Contact Information

1) For inquiries regarding chemotherapy

[redacted]

2) For inquiries regarding the study in general

JACCRO Study Secretariat

Phone: +81-3-6811-0433 E-mail: cc13.dc@jaccro.or.jp

List of Abbreviations and Definition of Terms

Abbreviation	Full name (English)
ALP	Alkaline Phosphatase
ALT	Alanine Aminotransferase
ANOVA	Analysis of Variance
AST	Aspartate Aminotransferase
BRAF	v–raf murine sarcoma viral oncogene homolog B1
CEA	Carcinoembryonic antigen
CT	Computed Tomography
CTCAE	Common Terminology Criteria for Adverse Events
CR	Complete Response
CRC	Clinical Research Coordinator
CT	Computed Tomography
DpR	Deepness of Response
ECOG	Eastern Cooperative Oncology Group
EGFR	Epidermal Growth Factor Receptor
ETS	Early Toumor shrinkage
FAS	Full Analysis Set
FLADS	Flexible License Assisted Data Server
FOLFIRI	FOLinic acid, 5-Fluorouracil, Irinotean
FOLFOX	FOLinic acid, 5-Fluorouracil, Oxaliplatin
FOLFOXIRI	FOLinic acid, 5-Fluorouracil, Oxaliplatin, Irinotecan
G-CSF	Granulocyte-colony Stimulating Factor
JACCRO	Japan Clinical Cancer Research Organization
KRAS	v-Ki-ras2 Kirsten Rat Sarcoma viral oncogene homolog
MRI	Magnetic Resonance Imaging
MST	Median Survival Time
NCI	National Cancer Institute
NE	Not Evaluable
NRAS	Neuroblastoma RAS viral oncogenehomolog
OS	Overall Survival
PD	Progressive Disease
PFS	Progression-Free Survival
PPS	Per Protocol Set
PPS	Post-Progression Survival

PR	Partial Response
PS	Performance Status
RAS	Rat Sarcoma
RECIST	Response Evaluation Criteria In Solid Tumors
RR	Response Proportion (Response Rate)
SD	Stable Disease
SDV	Source Data Verification
SP	Safety Population
TTF	Time to Treatment Failure
TTG	Time to Tumor Growth
US	Ultra Sonic

Table of Contents

0 Synopsis i

1 Background 1

1.1 Background and Rationale for the Present Study 1
1.2 Benefits and Disadvantages Expected from Participation in the Study 4

2 Study Objective 4

3 Study Design 4

3.1 Type of Study 4
3.2 Subjects 4
3.3 Study Endpoints 5
3.4 Allocation Adjustment Factors 5
3.5 Study Scheme 6
3.6 Target Sample Size 7
3.7 Rationale for the Target Sample Size 7
3.8 Interim Analysis 7

4 Study Drugs 8

4.1 Study Drug Name And Information 8
4.2 Handling of Generic Drugs 9

5 Subject Selection 9

5.1 Eligibility Criteria 9
5.2 Exclusion Criteria 11

6 Criteria Used in the Present Study 12

6.1 Description of Colorectal Cancer 12
6.2 Criteria for Determining the Tumor Reduction Effect 12
6.3 Criteria for Adverse Events 12
6.4 General Condition 13

7 Registration 13

7.1 Registration of Institutions and Investigators and Subinvestigators 13
7.2 Flads® System 13
7.3 Registration Office 13
7.4 Subject Registration Procedure 14
7.5 Important Notes Regarding Registration 14
7.6 Handling of Subjects Not Eligible for Registration 14

8 Protocol Treatment 14

8.1 Calculation of Body Surface Area 14
8.2 Group A: Folfoxiri + Bevacizumab Combination Therapy (Up To 12 Courses) 15
8.3 Group B: Folfoxiri + Cetuximab Combination Therapy (Up To 12 Courses) 16
8.4 Duration of Treatment 18
8.5 Criteria for Starting Protocol Treatment 18
8.6 Criteria for Continuing Treatment (In the 2nd and Subsequent Courses) 19
8.7 Criteria for Dose Reduction (In the 2nd and Subsequent Courses) 26
8.8 Criteria for Discontinuation of 5-Fu, Irinotecan, Oxaliplatin, Bevacizumab, and Cetuximab 2
8.9 Dose Increase 3

9 Concomitant Therapies 3

9.1 Prohibited Concomitant Therapies 3
9.2 Permitted Concomitant Drugs 4

9.3 Contraindications for Coadministration and Precautions for Coadministration 4
9.4 Recommended Supportive Therapy 4

10 Criteria for Discontinuation of the Protocol Treatment for Individual Subjects 5

11 Follow-Up of Discontinued Subjects 5

12 Subsequent Treatment (Secondary Treatment) 6

13 Anticipated Adverse Drug Reactions 6

13.1 Adverse Drug Reactions Expected With Individual Drugs 6
13.2 Adverse Reactions Expected with Combination Chemotherapy, Chemoradiotherapy, and Combined Modality Therapy 7

14 Expected Duration of Subject's Participation in the Study 7

15 Tests, Observations, and Examinations and Timing 7

15.1 Subject Characteristics 7
15.2 Tests, Observations, and Examinations 8
15.3 Follow-Up 8
15.4 Observations and Examinations and Schedule 9

16 Endpoints And Evaluation Criteria 9

16.1 Endpoints 9
16.2 Definitions/Criteria of The Endpoints 10

17 Submission of Reports 11

18 Reporting of Adverse Events 12

18.1 Definitions of Terms 12
18.2 Types of Adverse Events Requiring Reporting 13
18.3 Time Frames for Reporting by the Investigator or Subinvestigator 14
18.4 Procedures for Reporting And Responding to Serious Adverse Events 14

19 Data Accumulation and Statistical Analysis 17

19.1 Accumulation and Handling of Data 17
19.2 Statistical Analysis of Data 17
19.3 Analysis Sets 17
19.4 Analysis of The Primary Endpoint 17
19.5 Secondary Endpoints and The Analysis 17
19.6 Significance Level And Confidence Coefficient 18
19.7 Final Analysis 19

20 Discontinuation or Suspension of the Entire Study 19

21 Study Periods 19

22 Ethical Considerations 19

22.1 Regulations to Be Complied With 19
22.2 Information Provided for Subjects And Informed Consent 19
22.3 Items to Be Explained 20
22.4 Ethics Review Committee Approval 20
22.5 Protection of Privacy 21
22.6 Protocol Compliance 21
22.7 Compensation for Health Damage 21

23 Deviations From or Modifications of The Protocol 21

23.1 Modification of the Protocol 21
23.2 Modification of the Written Information for Subjects 22
23.3 Deviations from the Protocol 22

24 Quality Control and Quality Assurance **22**

24.1 Central Monitoring 22
24.2 Monitoring at Individual Medical Institutions 22
24.3 Auditing 23

25 Retention of Specimens and Records **23**

25.1 Retention of Specimens 23
25.2 Retention of Records 23

26 Contents and Methods of Reporting to the Head of Each Medical Institution **23**

27 Conflict of Interest and Cost Burden of The Study **24**

27.1 Source of Funds and Financial Relationship 24
27.2 Conflict of Interest 24
27.3 Costs Related to Study Treatment 24

28 Publication and Attribution of Study Results **24**

29 Pre-Registration of Study Protocol **25**

30 Study Implementation System **25**

30.1 Principal Investigator 25
30.2 Planning and Promotion Committee Members 25
30.3 External Response Review Committee Members 26
30.4 Statistical Analysis Manager 26
30.5 Data and Safety Monitoring Committee Members 26
30.6 Participating Institutions and Investigators 26
30.7 Jaccro Study Secretariat 26
30.8 Contact Information 27

31 References **28**

1 Background

1.1 Background and Rationale for the Present Study

Cetuximab, which is a human-mouse chimeric monoclonal antibody of the IgG1 subclass directed against the epidermal growth factor receptor (EGFR), binds to the human EGFR with high affinity and inhibits the growth of various EGFR-positive cancer cell lines in vitro in a concentration-dependent manner. Cetuximab has been shown to be useful in the treatment of curatively unresectable metastatic/recurrent colorectal cancer when administered with chemotherapy and administered alone.

The CRYSTAL study in patients with untreated metastatic/recurrent colorectal cancer[1] demonstrated the superiority of FOLFIRI + cetuximab as first-line therapy over FOLFIRI, with a significantly improved primary endpoint of progression-free survival, irrespective of their *KRAS* mutation status. Moreover, the hazard ratio for progression-free survival among patients with *KRAS* exon 2 wild type tumors was 0.696 (p=0.0012) and the hazard ratio for overall survival was 0.796 (p=0.0093), both showing significant differences. The OPUS study comparing FOLFOX4 with FOLFOX4 + cetuximab[2] showed significant additional benefits of the combination therapy in response rate and progression-free survival to patients with *KRAS* exon 2 wild type tumors: the response rate was 34.0% for FOLFOX4 and 57.3% for FOLFOX4 + cetuximab (p=0.0027) and the median progression-free survival was 7.2 months for FOLFOX4 and 8.3 months for FOLFOX4 + cetuximab (p=0.0064).

Clinical studies in patients with unresectable metastatic/recurrent colorectal cancer have been also conducted consecutively by the JACCRO. In a phase II study of cetuximab and mFOLFOX6 in metastatic colorectal cancer (JACCRO CC-05),[3] the response rate was 66.7%, the median progression-free survival was 11.05 months, and the median survival time was 29.66 months. In addition, 80% of patients achieved early tumor shrinkage at Week 8 (>20%), and had significantly improved median progression-free survival (11.3 months vs. 3.6 months; HR, 0.26; 95%CI, 0.13 to 0.61; p=0.0003) and median survival time (not reached vs. 9.0 months; HR, 0.34; 95%CI, 0.15 to 0.90; p=0.0143), compared with patients not achieving early tumor shrinkage. In a phase II study of cetuximab in combination with S-1 and oxaliplatin in first-line treatment for metastatic colorectal cancer (JACCRO CC-06),[4] the response rate was 66.7%, the median progression-free survival was 9.07 months, and the median survival time was not reached, with a 1-year survival of 83.5%. In addition, 72.4% of patients achieved early tumor shrinkage at Week 8 (>20%). Moreover, the results of a phase II study of cetuximab rechallenge in the third-line therapy in patients with *KRAS* wild-type metastatic colorectal cancer (JACCRO CC-08) conducted by the JACCRO are awaited.

With regard to chemotherapy combined with a molecularly targeted drug for the treatment of metastatic/recurrent colorectal cancer, the National Comprehensive Cancer Network (NCCN) Clinical Practice Guidelines version 1.2015[5] recommend FOLFOX4/modified FOLFOX6 or FOLFIRI as the platform regimen, combined with a molecularly targeted drug, bevacizumab, cetuximab, or panitumumab, as the current standard first-line therapy for unresectable metastatic/recurrent colorectal cancer. Although the guidelines recommend that anti-EGFR antibody drugs should be selected according to *RAS* mutation status reflecting tumor somatic mutations, all recommended regimens are category 2A. In addition, TRIBE study comparing FOLFOXIRI + bevacizumab combination therapy with FOLFIRI + bevacizumab

combination therapy in patients with unresectable metastatic colorectal cancer[6] demonstrated the superiority of the FOLFOXIRI + bevacizumab group. Since a subanalysis of progression-free survival also showed the superiority of the FOLFOXIRI + bevacizumab group, irrespective of *KRAS* exon 2 mutation status, FOLFOXIRI + bevacizumab combination therapy is regarded as a promising option for first-line regimens. The JSCCR Guidelines for the Treatment of Colorectal Cancer in Japan (2014 edition for physicians)[7] have added the FOLFOXIRI regimen as an option for first-line therapy for unresectable metastatic/recurrent colorectal cancer.

For the FOLFOXIRI regimen, recommended doses were determined in a European phase I study conducted by Falcone et al.[8] as followes: 175 mg/m^2 for irinotecan, 100 mg/m^2 for oxaliplatin, 3800 mg/m^2 for 5-FU, and 200 mg/m^2 for l-LV. However, in a phase II study using these recommended doses,[9] grade 3 or higher neutropenia occurred in 86% (grade 4 in 55%) and febrile neutropenia occurred in 14% of patients, and granulocyte colony-stimulating factor was required by many patients. Based on the results of this phase II study, Falcone et al., members of the Gruppo Oncologico Nord Ovest (GONO), conducted a phase III study.[10] In this study, the previous doses were changed to as follows: 165 mg/m^2 for irinotecan, 85 mg/m^2 for oxaliplatin, 3200 mg/m^2 for 5-FU, and 200 mg/m^2 for l-LV. Furthermore, TRIBE study using these new doses that compared FOLFOXIRI + bevacizumab combination therapy with FOLFIRI + bevacizumab combination therapy[6] reported that the actually administered doses of 5-FU, irinotecan, and oxaliplatin corresponded to 75% of the respective predefined standard doses. This results suggest that further dose adustments of individual drugs included in the FOLFOXIRI regimen may be needed to ensure the continuity of treatment.

A phase I study of the FOLFOXIRI regimen conducted in Japan[11] reported that the recommended doses were 150 mg/m^2 for irinotecan, 85 mg/m^2 for oxaliplatin, and 2400 mg/m^2 for 5-FU, and the use of these doses resulted in a response rate of 89%. The doses of the individual drugs are the same as those of FOLFOX and FOLFIRI used in Japan, and this dosage regimen may be appropriate to ensure the continuity of treatment with FOLFOXIRI. In future prospective studies of treatment with FOLFOXIRI conducted in Japan, the use of the doses in this phase I study is appropriate, and it is considered significant to evaluate the continuity and efficacy of the treatment.

In CALGB/SWOG 80405 study that directly compared cetuximab with bevacizumab,[12] chemotherapy combined with cetuximab did not prolong survival time significantly compared with chemotherapy combined with bevacizumab in patients with *RAS* wild-type tumors. In FIRE-3 study,[13] *RAS* mutational analyses showed that FOLFIRI combined with cetuximab significantly prolonged survival time by 8.1 months compared with FOLFIRI combined with bevacizumab in patients with *RAS* wild-type tumors. Because of different platform regimens for combination used in these two studies, it is not feasible to discuss the superiority of cetuximab based on combined results of the studies, and currently, there is no choice but to conclude that cetuximabis is as effective as bevacizumab for first-line treatments. However, there is an intersting report on tumor shrinkage as a secondary endpoint of FIRE-3 study. Early tumor shrinkage (ETS) and deepness of response (DpR) were superior with FOLFIRI combined with cetuximab. In a retrospective analysis study conducted by the GONO,[14] a comparison of results between FOLFOXIRI

combined with anti-EGFR antibody therapy (cetuximab or panitumumab) and FOLFOXIRI combined with bevacizumab showed that the DpR at Month 4 was 48.6% and 37.8%, respectivley, and the median ETS at Week 8 was 40.8% and 26.4%, respectively, indicating significantly better results with FOLFOXIRI combined with anti-EGFR antibody therapy in patients with *RAS*/*BRAF* wild type tumors. The possibility of ETS and DpR being appropriate as new surrogate endpoints for survival time has been reported in FIRE-3 study[13] as well as in a subgroup analysis in TRIBE study.[15] In addition, a study[16] that analyzed the relationship between post-progression survival (PPS) after first-line treatment and DpR observed in CRYSTAL study[1] and OPUS study[2] has shown that DpR correlates with PPS and directly relates to prolongation of OS. It is therefore scientifically interesting to further investigate these new endpoints in future prospective clinical studies.[17]

Table 1.1 Median DpR Observed in Previous Clinical Studies

Study name	Definition of DpR/ Patient population	Median DpR % (S.D.) Anti-EGFR antibody group	Median DpR % (S.D.) Control group	Difference in median DpR
1.CRYSTAL[16]	DpR until PD *KRAS* wt	50.9 (44.6) * FOLFIRI + cetuximab	33.3 (37.1)* FOLFIRI	17.6%
2. OPUS[16]	DpR until PD *KRAS* wt	57.9 (51.1)* FOLFOX4 + cetuximab	30.7 (38.5)* FOLFOX4	27.2%
3. GONO [14]	DpR by month 4 *RAS/BRAF* wt	48.6 (28.7) FOLFOXIRI + cetuximab or panitumumab	37.8 (19.8) FOLFOXIRI + bevacizumab	10.8%
4. FIRE-3[13]	DpR until PD *RAS* wt	48.9 (54.8) FOLFIRI + cetuximab	32.3 (42.3) FOLFIRI + bevacizumab	16.6%
5. PEAK[18]	DpR until PD *RAS* wt	65 (29)* mFOLFOX6 + panitumumab	46 (25)* mFOLFOX6 + bevacizumab	19%
Average between 4 and 5		57 (42)	39.2 (33.7)	18.5%
	*Calculated from the interquartile range			

As described earlier, the phase I study of the FOLFOXIRI regimen conducted in Japan[11] reported that the recommended doses were 150 mg/m^2 for irinotecan, 85 mg/m^2 for oxaliplatin, and 2400 mg/m^2 for 5-FU, and the use of these doses resulted in a response rate of 89%. The doses of the individual drugs are the same as those of FOLFOX and FOLFIRI used in Japan, and this dosage regimen may be appropriate to ensure the continuity of treatment with FOLFOXIRI. Based on these results, it is considered significant to evaluate the efficacy of FOLFOXIRI + cetuximab combination therapy as first-line treatment for patients with unresectable metastatic/recurrent colorectal cancer in Japan.

Since there have been few studies that compare cetuximab with bevacizumab in combination with the FOLFOXIRI regimen used as a platform, it is significant to investigate whether cetuximab that produces significant tumor shrinkage will be more effective than bevacizumab when combined with the FOLFOXIRI regimen as a promising treatment option. Moreover, it would be scientifically interesting to prospectively compare these two antibody drugs using a primary endpoint of DpR, which may reflect survival time. For these reasons, we have planned to use the FOLFOXIRI regimen with the recommended doses in Japan as a platform for chemotherapy to conduct a phase II randomized study to compare FOLFOXIRI + cetuximab

combination therapy with FOLFOXIRI + bevacizumab combination therapy in terms of usefulness as first-line therapy for patients with *RAS* wild type, unresectable metastatic/recurrent colorectal cancer.

Furthermore, in recent years, the possibility that *BRAF* status other than *RAS* mutations is associated with the therapeutic effect of anti-EGFR antibody drugs has been investigated[22]. In our study, it is possible that differences in therapeutic effects will appear depending on *BRAF* status, and by examining the therapeutic effects of each *BRAF* status, it will be possible to clarify the most useful target group for FOLFOXIRI + cetuximab combination therapy.

Therefore, we planned a randomized phase II trial for the first-line treatment of *RAS* wild-type unresectable advanced/recurrent colorectal cancer, using the recommended dose of FOLFOXIRI therapy in Japan as a platform for chemotherapy, to compare the usefulness of FOLFOXIRI + cetuximab combination therapy and FOLFOXIRI + bevacizumab combination therapy.

1.2 Benefits and Disadvantages Expected from Participation in the Study

Expected benefits: Participation in this study may provide therapeutic effects equal to or better than conventional standard therapy, thereby leading to prolongation of survival time.

Expected disadvantages and risks: All drugs used in this study are approved for health insurance coverage and are provided as medical treatments under health insurance. Except for some expenses paid by subjects, all medical expenses, including drug expenses, for subjects during the study period will be covered by health insurance of the subjects; therefore, no economic benefit will be provided for the subjects. Moreover, adverse drug reactions listed in Section 13 “Anticipated Adverse Drug Reactions” may occur in subjects, and treatment-related deaths may occur in some of the subjects. However, since these cases can also occur in the actual medical practice, participation in the study will not necessarily increase the risk.

2 Study Objective

To demonstrate the superiority of FOLFOXIRI plus cetuximab over FOLFOXIRI plus bevacizumab in patients with *RAS* wild-type, unresectable metastatic/recurrent colorectal cancer

3 Study Design

3.1 Type of Study

A multicenter, randomized, comparative phase II study (open-label) using the central registration method

3.2 Subjects

Patients with *RAS* wild-type (*KRAS* exon 2, 3, 4, *NRAS* exon 2, 3, 4), unresectable metastatic/recurrent colorectal cancer with a measurable lesion according to RECIST criteria (Ver.1.1)

3.3 Study Endpoints

3.3.1 Primary Endpoint

Deepness of response (DpR)*

*Deepness of response (DpR) is defined as the sum of the longest diameters of RECIST target lesions at the nadir in the absence of progression subtracted from the sum of the longest diameters of RECIST target lesions at baseline divided by the sum of the longest diameters of RECIST target lesions at baseline.

3.3.2 Secondary Endpoints

Early tumor shrinkage (ETS) at Week 8

Response rate (RR)

Deepness of response (DpR) by Month 4

Time to treatment failure (TTF)

Time to tumor growth (TTG)

Progression-free survival (PFS)

Overall survival (OS)

Association between tumor shrinkage (ETS, RR, DpR) and prognosis (PFS, OS)

Association between TTG and prognosis (OS)

Resection rate

R0 resection rate

Safety (incidence and severity of adverse events)

3.4 Allocation Adjustment Factors

- Primary tumor site (right or left)
- History of postoperative adjuvant chemotherapy

(None, postoperative adjuvant chemotherapy including oxaliplatin, postoperative adjuvant chemotherapy not including oxaliplatin)

- PS (0, 1)

3.5 Study Scheme

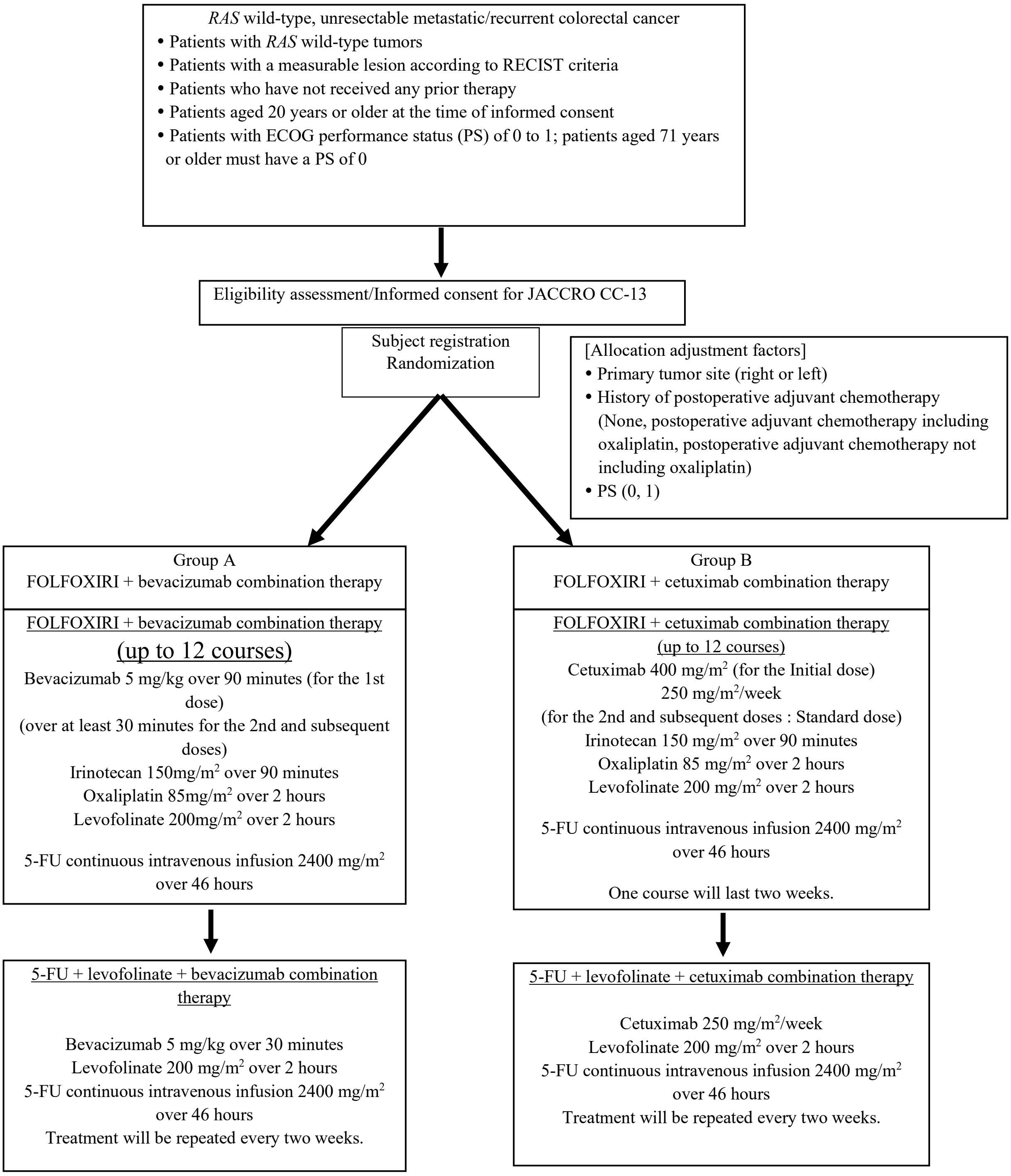

Figure 3.5 Scheme

3.6 Target Sample Size

180 subjects per group, 360 subjects in total

3.7 Rationale for the Target Sample Size

In FIRE-3 study and PEAK study that compared anti-EGFR therapy with bevacizumab therapy, the difference in the median DpR between the two arms ranged from 16.6% to 19%, and the chemotherapy platform used in these studies was 2-drug combination therapy, FOLFIRI or FOLFOX. In a retrospective study conducted by the GONO using 3-drug combination therapy, FOLFOXIRI, as the platform, the difference in the median DpR at Month 4 between anti-EGFR therapy and bevacizumab therapy was 10.8%.

Table 3.7 Median DpR Observed in Previous Clinical Studies

Study name	Definition of DpR/ Patient population	Median DpR % (S.D.) Anti-EGFR antibody group	Median DpR % (S.D.) Control group	Difference in median DpR
1.CRYSTAL[16]	DpR until PD<br>*KRAS* wt	50.9 (44.6) *<br>FOLFIRI + cetuximab	33.3 (37.1)*<br>FOLFIRI	17.6%
2. OPUS[16]	DpR until PD<br>*KRAS* wt	57.9 (51.1)*<br>FOLFOX4 + cetuximab	30.7 (38.5)*<br>FOLFOX4	27.2%
3. GONO[14]	DpR by month 4<br>*RAS/BRAF* wt	48.6 (28.7)<br>FOLFOXIRI + cetuximab or panitumumab	37.8 (19.8)<br>FOLFOXIRI + bevacizumab	10.8%
4. FIRE-3[13]	DpR until PD<br>*RAS* wt	48.9 (54.8)<br>FOLFIRI + cetuximab	32.3 (42.3)<br>FOLFIRI + bevacizumab	16.6%
5. PEAK[18]	DpR until PD<br>*RAS* wt	65 (29)*<br>mFOLFOX6 + panitumumab	46 (25)*<br>mFOLFOX6 + bevacizumab	19%
Average between 4 and 5		57 (42)	39.2 (33.7)	18.5%
	*Calculated from the interquartile range			

Based on these results, assuming a difference in the median DpR between the FOLFOXIRI + cetuximab combination therapy group and the FOLFOXIRI + bevacizumab combination therapy group of 12.5%, with standard deviations of 42% (cetuximab therapy) and 34% (bevacizumab therapy), respectively, the required sample size with a two-sided significance level of 0.05 and a statistical power of 0.85 was calculated to be 338 subjects. The final sample size was calculated to be 360 subjects, taking a 5% dropout rate, including the rate of withdrawals before administration, into consideration.

3.8 Interim Analysis

3.8.1 When the DpR in 20 Subjects per Group is Available

Taking into consideration that the sample size calculation shown in Section 3.7 “Rationale for the Target Sample Size” is based in the results of a retrospective analysis and that there is a concern about the safety of FOLFOXIRI, the Data and Safety Monitoring Committee will examine the results of a futility analysis when the DpR in 20 subjects per group is available. If the analysis results show that the mean DpR in the FOLFOXIRI + cetuximab combination therapy group is inferior to that in the FOLFOXIRI +

bevacizumab combination therapy group by at least 5%, the Data and Safety Monitoring Committee will consider actions, including discontinuation of the study, and make a recommendation to the Principal Investigator.

3.8.2 When the DpR at month 4 in 40 Subjects per Group is Available

When the DpR in 40 subjects per group is available, an interim analysis for recalculation of the required sample size will be performed.

The Data and Safety Monitoring Committee will examine the calculated mean DpR at month 4 in 40 subjects per group and its standard deviation.

(1) If the mean DpR in the FOLFOXIRI + cetuximab combination therapy group is inferior to that in the FOLFOXIRI + bevacizumab combination therapy group, The Data and Safety Monitoring Committee will recommend futility stop of the study to the Principal Investigator.

(2) If the mean DpR in the FOLFOXIRI + cetuximab combination therapy group is not inferior to that in the FOLFOXIRI + bevacizumab combination therapy group and there is no significant safety concern about the treatment in both groups:

1) If the calculated standard deviation is as estimated, the study will be continued as scheduled.

2) If the calculated standard deviation is larger than the estimate, the Principal Investigator will determine whether to continue the study by recalculating the required sample size so as to provide a statistical power of 0.85 based on the standard deviation. If the recalculation produces an infeasible required sample size, the study will be discontinued.

3) If the calculated standard deviation is smaller than the estimate, the Principal Investigator will determine whether to decrease the required sample size based on the standard deviation or whether to continue the study by increasing the statistical power with the predefined required sample size unchanged.

In addition, the Data and Safety Monitoring Committee will disclose the percentage of subjects with unevaluable DpR at the time of the interim analysis to the Principal Investigator for recalculation of the required sample size. The results of examination by the Principal Investigator will be submitted to the Data and Safety Monitoring Committee for deliberation.

4 Study Drugs

Drugs used in this study are described below. Refer to the latest package insert of each drug for detailed information and the handling of 5-FU, levofolinate, irinotecan, oxaliplatin, cetuximab, and bevacizumab.

4.1 Study Drug Name and Information

(1) 5-FU

1) Generic name: Fluorouracil

2) Brand name: 5-FU Injection; Dosage form/Dose: injection 250 mg, etc.

3) Drug information: Refer to the package insert.

(2) Irinotecan

1) Generic name: Irinotecan hydrochloride hydrate

2) Brand name: CAMPTO for I.V. Infusion; Dosage form/Dose: injection 40 mg/100 mg

3) Drug information: Refer to the package insert.

(3) Oxaliplatin

1) Generic name: Oxaliplatin

2) Brand name: ELPLAT I.V. INFUSION SOLUTION; Dosage form/dose: injection 50 mg/100 mg, etc.

3) Drug information: Refer to the package insert.

(4) Levofolinate

1) Generic name: Levofolinate calcium

2) Brand name: LEVOFOLINATE FOR INTRAVENOUS INFUSION; Dosage form/Dose: injection 25 mg/100 mg, etc.

3) Drug information: Refer to the package insert.

(5) Bevacizumab

1) Generic name: Bevacizumab (Genetical Recombination)

2) Brand name: AVASTIN; Dosage form/Dose: injection 100 mg/400 mg

3) Drug information: Refer to the package insert.

(6) Cetuximab

1) Generic name: cetuximab (Genetical Recombination)

2) Brand name: ERBITUX Injection; Dosage form/Dose: injection 100 mg

3) Drug information: Refer to the package insert.

4.2 Handling of Generic Drugs

Use of generic drugs will be allowed if they become available.

5 Subject Selection

5.1 Eligibility Criteria

Patients meeting all of the following criteria are eligible for this study. Gender is not a criterion.

(1) Patients with histologically confirmed colorectal cancer

(2) Patients with *RAS* wild-type, unresectable metastatic/recurrent colorectal cancer

(3) Patients with a measurable lesion according to RECIST criteria (Ver.1.1)

(4) Patients with unresectable primary tumors or unresectable distant or lymph node metastases who have not received any prior chemotherapy (patients undergoing surgery must have not received any treatment other than surgery)

Patients with first recurrence after surgery for primary tumors or metastases who have not received any treatment for the recurrence including surgery (patients undergoing postoperative adjuvant therapy must have experienced relapse more than 12 months after completion of the therapy)

*See Figure 5.1.1 “Eligible patients with unresectable primary tumors or unresectable distant or lymph node metastases” and Figure 5.1.2 “Eligible patients with first recurrence.”

(5) Patients aged 20 years or older at the time of informed consent

(6) Patients with ECOG performance status (PS) of 0 to 1; patients aged 71 years or older must have a PS of 0

(7) Patients with an estimated life expectancy of 6 months or more

(8) Patients who meet all of the following laboratory criteria for major organ functions within 14 days of enrollment

If there are multiple laboratory data during the period, the most recent data before enrollment should be used. If laboratory data are measured newly, no blood transfusion or administration of hematopoietic factor preparations is permitted within 14 days of the measurement.

1) White blood cell count ≥ 3,000/mm^3 and < 12,000/mm^3
2) Neutrophil count ≥ 1,500/mm^3
3) Platelet count ≥ 10.0×10^4/mm^3
4) Hemoglobin ≥ 9.0 g/dL
5) Blood bilirubin ≤ 1.5 times the upper limit of the institutional normal range
6) AST ≤ 2.5 times the upper limit of the institutional normal range (≤ 5 times the upper limit of the institutional normal range if liver metastasis is present)
7) ALT ≤ 2.5 times the upper limit of the institutional normal range (≤ 5 times the upper limit of the institutional normal range if liver metastasis is present)
8) Serum creatinine ≤ 1.5 times the upper limit of the institutional normal range
9) Urine protein ≤ 1+
10) PT-INR ≤ 1.5

(9) Patients who have provided written consent after being fully informed of the contents of the present study

● <Eligible patients with unresectable primary tumors or unresectable distant or lymph node metastases>

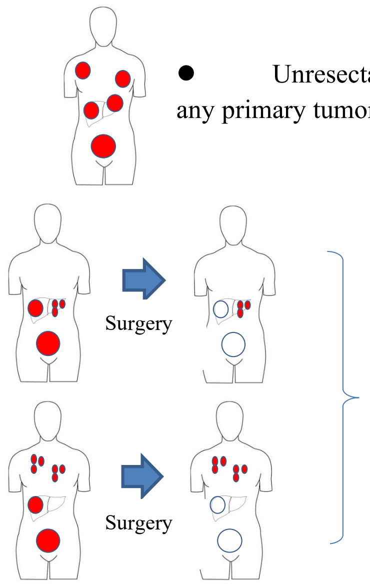

● Unresectable distant or lymph node metastases (with or without any primary tumor

● Residual metastases after resection of primary tumors or distant metastases

○ ● After resection

Figure 5.1.1 Eligible Patients with Unresectable Primary Tumors or Unresectable Distant or Lymph Node

Metastases

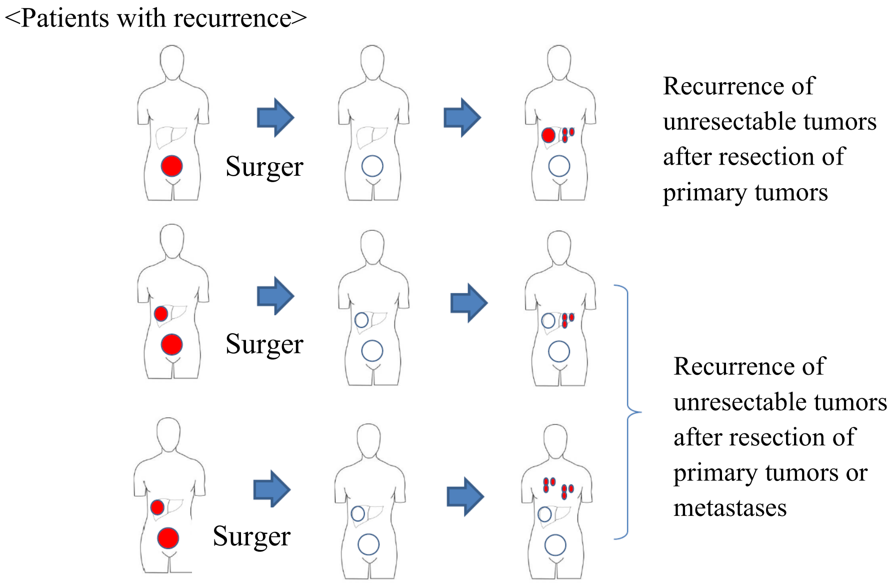

Figure 5.1.2 Eligible Patients with First Recurrence

5.2 Exclusion Criteria

Patients meeting any of the following criteria are excluded from this study.

(1) Patients with synchronous double cancers or metachronous double cancers with a disease-free interval less than 5 years (not excluding any early-stage cancer expected to be cured by topical therapy)

(2) Patients with suspected hereditary colorectal cancer for their family history or other reasons

(3) Patients with brain metastasis

(4) Patients with infectious disease

(5) Patients with interstitial pneumonia or pulmonary fibrosis

(6) Patients with concurrent or previous serious heart disease

(7) Patients with a history of thromboembolism

(8) Patients with cerebrovascular disease

(9) Patients with a history of hematemesis or hemoptysis

(10) Patients with poorly controlled hypertension

(11) Patients with severe paresthesia or dysesthesia accompanied by functional impairment

(12) Patients with a large amount of malignant fluid (pleural, abdominal, or pericardial effusion)

(13) Patients with serious comorbidities (e.g., renal failure, hepatic failure, hypertension)

(14) Patients who have undergone radiation therapy for primary tumors or metastases

(15) Men who are not willing to practice contraception, or pregnant women, lactating women, women with a positive pregnancy test, or women who are not willing to practice contraception

(16) Patients with a history of serious hypersensitivity

(17) Patients with a positive test for HBsAg or with active viral hepatitis

(18) Patients who have received blood transfusion, blood products, or hematopoietic factor preparations such as G-CSF within 14 days of enrollment

(19) Patients who have undergone surgical therapy, biopsy with skin incision, or surgical closure of trauma within 28 days of enrollment, or patients who have undergone fine-needle aspiration biopsy (excluding central venous port implantation) within 7 days of enrollment

(20) Patients who will require or currently receive any drug that suppresses platelet function (aspirin preparations at a daily dose of 325 mg or more or nonsteroidal anti-inflammatory drugs) for chronic inflammatory diseases such as rheumatoid arthritis

(21) Patients with a bleeding diathesis (including hemoptysis, or cavitation and/or necrosis of pulmonary metastases found by imaging studies), coagulopathy, or abnormalities in coagulation factors

(22) Patients with gastrointestinal tract perforation or a history of gastrointestinal tract perforation within 1 year of enrollment

(23) Patients with unhealed traumatic fracture

(24) Patients with watery stool or uncontrollable diarrhea

(25) Patients who have undergone organ transplantation requiring immunosuppressive drugs

(26) Patients who have been treated with cetuximab, bevacizumab, oxaliplatin, or irinotecan (excluding oxaliplatin used as postoperative adjuvant chemotherapy)

(27) Patients currently receiving atazanavir sulfate (REYATAZ)

(28) Patients with jaundice

(29) Patients with intestinal paresis or obstruction

(30) Any others considered to be ineligible for study participation by the investigator or subinvestigator

6 Criteria Used in the Present Study

6.1 Description of Colorectal Cancer

The Japanese Classification of Colorectal Carcinoma 8th Edition (July 2013) edited by the Japanese Society for Cancer of the Colon and Rectum will be used.

6.2 Criteria for Determining the Tumor Reduction Effect

The Response Evaluation Criteria in Solid Tumors (hereinafter referred to as RECIST) Version 1.1 will be used.

6.3 Criteria for Adverse Events

The Common Terminology Criteria for Adverse Events v4.0-JCOG (hereinafter referred to as CTCAE ver.4.0-JCOG) will be used.

6.4 General Condition

ECOG Performance Status Scale (hereinafter referred to as PS) (0 to 4) will be used. Patients with restricted activity due to local symptoms will be evaluated clinically.

7 Registration

7.1 Registration of Institutions and Investigators and Subinvestigators（After the certified review board approval）

(1) After obtaining approval of Manager of the each medical institution, the investigator or subinvestigator will send a copy of the written approval to the JACCRO Study Secretariat via fax or mail.

(2) The JACCRO Study Secretariat will register the institution and then register the investigator, the subinvestigator, the clinical research coordinator (CRC), and other personnel in the Flexible License Assisted Data Server (FLADS®) system (refer to Section 7.2 "FLADS® System").

(3) The investigator or subinvestigator will enter the institutional laboratory normal range into the FLADS® system.

7.2 FLADS® System

The FLADS® system,[19] which supports the procedures at individual steps of a clinical study via the Internet, allows the selection and registration of subjects and the calculation of doses based on body surface area while the personal information of each subject remained confidential. The system can send study discontinuation reports, follow-up reports, and serious adverse event reports through the Internet. Moreover the system can send data generated during the study to the JACCRO Study Secretariat for storage as well as automatically grade adverse events in terms of laboratory values and automatically calculate the degree of shrinkage based on entered tumor diameter at baseline and tumor diameter after treatment. In addition, CTCAE and other criteria are incorporated in the system.

Since its foundation in 2002, the JACCRO has conducted clinical studies using the FLADS® system.

For functions used in the present study and specific operational methods, refer to the separate instruction manual.

7.3 Registration Office

JACCRO Study Secretariat:
Nonprofit Organization Japan Clinical Cancer Research Organization (JACCRO)
6F Jinbocho Kyowa Building, 1-64-3 Kandajinbocho, Chiyoda -ku, Tokyo 101-0051, Japan
Phone: +81-3-6811-0433 Fax: +81-3-6811-0434
E-mail: cc13.dc@jaccro.or.jp

7.4 Subject Registration Procedure

Subjects will be registered by the central registration method according to the procedure described below.

(1) After obtaining written informed consent from each subject and confirming that the subject meets all of the eligibility criteria and none of the exclusion criteria, the investigator or subinvestigator will register the subject the FLADS® system.

(2) The JACCRO Study Secretariat will verify whether the subject is eligible or not in terms of the eligibility/exclusion criteria through the FLADS® system, and confirm any question with the investigator or subinvestigator.

(3) Once the subject is registered in the FLADS® system, a subject number will be issued automatically.

(4) After completion of the registration of the subject, the eligibility of the subject, the subject number, the dose to be administered, and the treatment schedule will be displayed on a registration confirmation sheet on the FLADS® system. The investigator or subinvestigator will download the registration confirmation sheet to confirm its content.

7.5 Important Notes Regarding Registration

(1) Once registered, the subject cannot cancel the registration (deletion from the database). In any case of duplicate registration, the initial registration information (subject number) will be adopted. When any erroneous or duplicate registration is found, the JACCRO Study Secretariat should be contacted immediately.

(2) The investigator or subinvestigator must not start the protocol treatment for each subject before completion of the registration of the subject.

7.6 Handling of Subjects not Eligible for Registration

When knowing that a subject is ineligible for registration throught the FLADS® system, the investigator or subinvestigator will explain the fact to the subject and provide treatment appropriate for the subject's disease condition.

8 Protocol Treatment

The protocol treatment will be started within 14 days of the registration of each subject in the FLADS® system.

8.1 Calculation of Body Surface Area

Body surface area will be calculated using the DuBois formula.

[DuBois formula]

Body surface area (m^2) = body weight (kg)$^{0.425}$ × body height (cm)$^{0.725}$ × 71.84 ÷ 10000

If a ≥5% change in body weight is observed, the dose of each drug should be corrected by recalculating the

body surface area. The timing of dose correction depends on each institution.

8.2 Group A: FOLFOXIRI + Bevacizumab Combination Therapy (up to 12 courses)

A 2-week course consists of the following treatments and each subject will receive up to 12 courses of therapy.

(1) Bevacizumab: Bevacizumab 5 mg/kg will be administered as an intravenous infusion over 90 minutes on Day 1 for the 1st dose. If the 1st dose is well tolerated, the 2nd dose may be administered over 60 minutes. If the 2nd dose is also well tolerated, subsequent doses may be administered over 30 minutes.

(2) Irinotecan: Irinotecan hydrochloride hydrate 150 mg/m^2 will be administered as an intravenous infusion over 90 minutes on Day 1.

(3) Oxaliplatin and levofolinate: Oxaliplatin 85 mg/m^2 and levofolinate 200 mg/m^2 will be administered as intravenous infusions at the same time over 2 hours on Day 1.

(4) 5-FU: After completion of the administration of oxaliplatin and levofolinate, 5-FU 2400 mg/m^2 will be administered as a continuous intravenous infusion over 46 hours.

►For patients homozygous for *UGT1A1*28* or *UGT1A1*6* or patients heterozygous for both *UGT1A1*28* and *UGT1A1*6*, irinotecan dose may be reduced to 125 mg/m^2 or 100 mg/m^2 at the discretion of the primary physician.

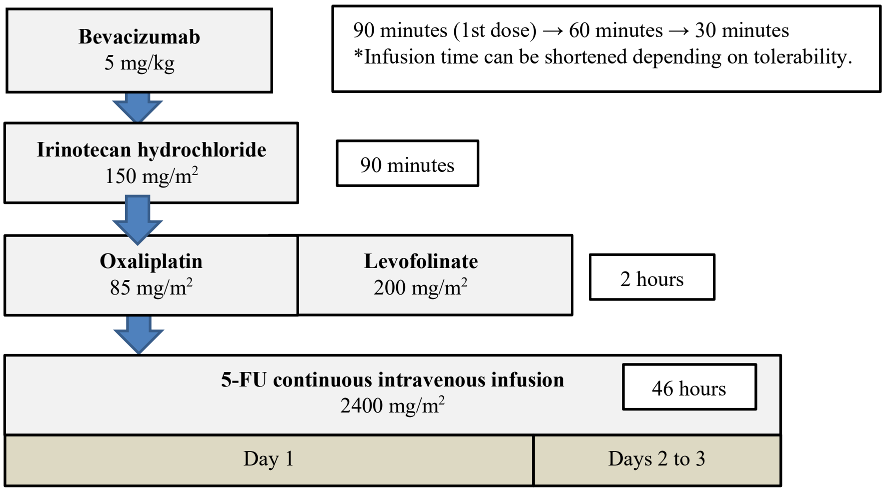

(Treatment will be repeated every two weeks.)

Figure 8.2 FOLFOXIRI + Bevacizumab Combination Therapy

FOLFOXIRI + bevacizumab combination therapy will be switched to 5-FU + levofolinate + bevacizumab combination therapy if either of the following is met:

- Up to 12 courses of FOLFOXIRI + bevacizumab combination therapy have been completed.
- A further dose reduction is needed after 2 dose-level reductions of irinotecan and oxaliplatin.

[5-FU + Levofolinate + Bevacizumab Combination Therapy]

(1) Bevacizumab: Bevacizumab 5 mg/kg will be administered as an intravenous infusion over 30 minutes.

(2) Levofolinate: Levofolinate 200 mg/m^2 will be administered as an intravenous infusion over 2 hours.

(3) 5-FU: After completion of the administration of levofolinate, 5-FU 2400 mg/m^2 will be administered as a continuous intravenous infusion over 46 hours. If any dose reduction of 5-FU has been made during FOLFOXIRI + bevacizumab combination therapy, the same dose will be continued.

8.3 Group B: FOLFOXIRI + Cetuximab Combination Therapy (up to 12 courses)

Cetuximab will be administered once every week and FOLFOXIRI will be administered once every two weeks.

A 2-week course consists of the following treatments and each subject will receive up to 12 courses of therapy.

(1) Cetuximab:

Before administration of cetuximab, premedication specified at each center will be given for the prevention of infusion reactions to cetuximab and nausea/vomiting induced by irinotecan.

(e.g.)

- H1 antagonists (e.g., oral administration of diphenhydramine hydrochloride preparation 50 mg)
- 5-HT3 receptor antagonists (e.g., intravenous infusion of granisetron hydrochloride 3 mg)
- Corticosteroids (e.g., intravenous infusion of dexamethasone sodium phosphate 8 mg)

Cetuximab will be administered once weekly. For the 1st dose on Day 1, 400 mg/m^2 of cetuximab dissolved in 500 mL of isotonic sodium chloride solution will be administered as an intravenous infusion at an infusion rate of ≤5 mg/min over at least 120 minutes. For the 2nd and subsequent doses (Standard dose) , 250 mg/m^2 of cetuximab dissolved in 250 mL of isotonic sodium chloride solution will be administered as an intravenous infusion at an infusion rate of ≤10 mg/min over at least 60 minutes. Doses should be separated by at least 1 week.

If any grade 1 infusion reaction occurs during the infusion, the infusion rate should be further reduced (e.g., the infusion rate should be reduced by 50%: from ≤10 mg/min to ≤5 mg/min).

If any grade 2 infusion reaction occurs during the infusion, the subject should be observed while infusion is being interrupted. Then, infusion should be continued with care at a further reduced infusion rate (e.g., the infusion rate should be reduced by 50%: from ≤10 mg/min to ≤5 mg/min).

If any infusion reaction occurs again after infusion is resumed at a further reduced infusion rate, administration of cetuximab should be discontinued.

If any grade 3 infusion reaction occurs, administration should be discontinued immediately and

should not be repeated.

(2) Irinotecan: Irinotecan hydrochloride hydrate 150 mg/m^2 will be administered as an intravenous infusion over 90 minutes on Day 1.

(3) Oxaliplatin 85 mg/m^2 and levofolinate 200 mg/m^2 will be administered as intravenous infusions at the same time over 2 hours on Day 1.

(4) 5-FU: After completion of the administration of oxaliplatin and levofolinate, 5-FU 2400 mg/m^2 will be administered as a continuous intravenous infusion over 46 hours.

►For patients homozygous for *UGT1A1*28* or *UGT1A1*6* or patients heterozygous for both *UGT1A1*28* and *UGT1A1*6*, irinotecan dose may be reduced to 125 mg/m^2 or 100 mg/m^2 at the discretion of the primary physician.

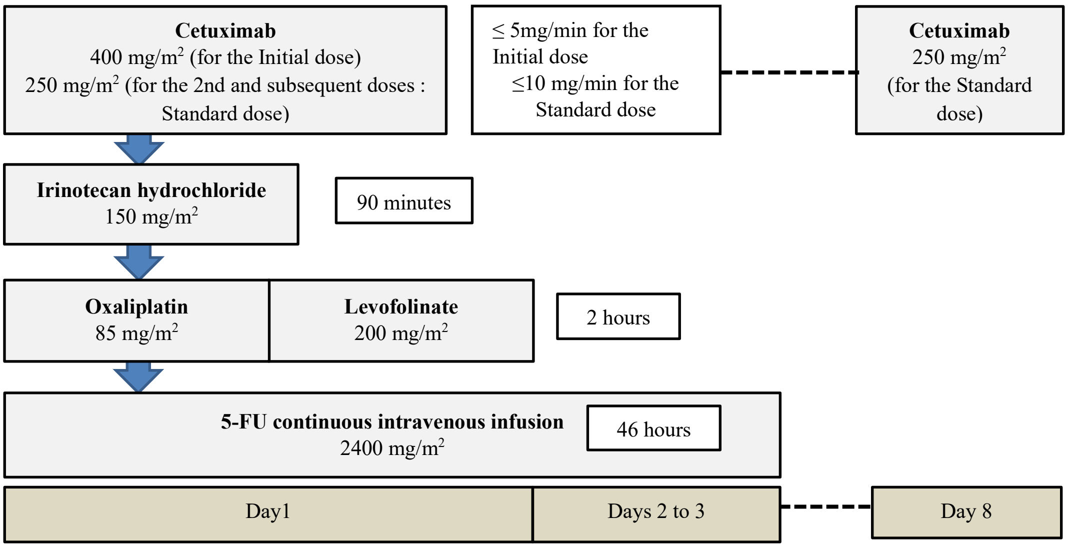

(Treatment will be repeated every two weeks.)

Figure 8.3 FOLFOXIRI + Cetuximab Combination Therapy

FOLFOXIRI + cetuximab combination therapy will be switched to 5-FU + levofolinate + cetuximab combination therapy if either of the following is met:

- Up to 12 courses of FOLFOXIRI + cetuximab combination therapy have been completed.
- A further dose reduction is needed after 2 dose-level reductions of irinotecan and oxaliplatin.

[5-FU + Levofolinate + Cetuximab Combination Therapy]

Cetuximab will be administered once every week and levofolinate + 5-FU will be administered once every two weeks.

(1) Cetuximab: Cetuximab 250 mg/m^2, dissolved in 250 mL isotonic sodium chloride solution, will be administered as an intravenous infusion at an infusion rate of ≤10 mg/min over at least 60 minutes. Doses should be separated by at least 1 week.

If any grade 1 infusion reaction occurs during the infusion, the infusion rate should be further reduced (e.g., the infusion rate should be reduced by 50%: from ≤10 mg/min to ≤5 mg/min).

If any grade 2 infusion reaction occurs during the infusion, the subject should be observed while infusion is being interrupted. Then, infusion should be continued with care at a further reduced infusion rate (e.g., the infusion rate should be reduced by 50%: from ≤10 mg/min to ≤5 mg/min).

(2) Levofolinate: Levofolinate 200 mg/m^2 will be administered as an intravenous infusion over 2 hours.

(3) 5-FU: After completion of the administration of levofolinate, 5-FU 2400 mg/m^2 will be administered as a continuous intravenous infusion over 46 hours. If any dose reduction of 5-FU has been made during FOLFOXIRI + cetuximab combination therapy, the same dose will be continued.

8.4 Duration of Treatment

Treatment will be continued unless each subject meets any of the "Criteria for Discontinuing the Protocol Treatment for Individual Subjects" (Section 10).

8.5 Criteria for Starting Protocol Treatment

The protocol treatment should be started after confirming that the laboratory values and clinical symptoms on the day before or on the starting day of treatment meet the respective criteria shown in Table 8.5 "Criteria for Starting Protocol Treatment."

Any subject who fails to meet any of the criteria shown in Table 8.5 "Criteria for Starting Protocol Treatment" within 14 days of registration and cannot start the protocol treatment will be regarded as a withdrawal before treatment.

Table 8.5 Criteria for Starting Protocol Treatment

Item	Criteria
White blood cell count	≥3,000/mm^3, <12,000/mm^3
Neutrophil count	≥1,500/mm^3
Platelet count	≥10.0 × 10^4/mm^3
Hemoglobin	≥9.0 g/dL
Blood bilirubin	≤1.5 times the upper limit of the institutional normal range
AST	≤2.5 times the upper limit of the institutional normal range (≤5 times the upper limit of the institutional normal range for subjects with liver metastasis)
ALT	
Serum creatinine	≤1.5 times the upper limit of the institutional normal range
Infection	Absence of fever ≥38.0°C suggestive of infection
Blood pressure	Absence of poorly controlled hypertension
PT-INR	≤1.5
Diarrhea, oral mucositis, skin eruption	≤ Grade 1
Proteinuria	≤ Grade 1
Thromboembolism	Absence of this condition
Bleeding	Absence, or mild bleeding requiring no treatment
Other non-hematological findings (excluding constipation, anorexia, nausea,	≤ Grade 1

vomiting, alopecia, skin hyperpigmentation, dysgeusia, fatigue, malaise, laboratory abnormalities, and other adverse events considered by the investigator to be non-problematic the initiation of treatment)	

8.6 Criteria for Continuing Treatment (in the 2nd and subsequent courses)

8.6.1 Group A: Criteria for Continuing FOLFOXIRI + Bevacizumab Combination Therapy (up to the 12th course)

In Group A, the protocol treatment in the 2nd and subsequent courses should be continued after confirming that the laboratory values and clinical symptoms on the day before or on the starting day of treatment meet the respective criteria shown in both Table 8.6.1.1 “Criteria for Continuing FOLFOXIRI Therapy (in the2nd and subsequent courses)” and Table 8.6.1.2 “Criteria for Continuing Bevacizumab Therapy.”

(1) If the criteria shown in Table 8.6.1.1 “Criteria for Continuing FOLFOXIRI Therapy (in the 2nd and subsequent courses)” are not meet, but the criteria shown in Table 8.6.1.2 “Criteria for Continuing Bevacizumab Therapy” are met:
FOLFOXIRI therapy should be delayed.
In this case, it is recommended to give the next FOLFOXIRI therapy around 7 days later (around Day 22 of the previous course).
While FOLFOXIRI therapy is omitted, bevacizumab should not be administered alone.

(2) If the criteria shown in Table 8.6.1.1 “Criteria for Continuing FOLFOXIRI Therapy (in the 2nd and subsequent courses)” are met, but the criteria shown in Table 8.6.1.2 “Criteria for Continuing Bevacizumab Therapy” are not met:
FOLFOXIRI therapy alone should be given and bevacizumab therapy should be skipped.

(3) Bevacizumab therapy may be skipped if the investigator or subinvestigator considers it necessary to do so due to the occurrence of any adverse event that does not fall under the criteria shown in Table 8.6.1.2 “Criteria for Continuing Bevacizumab Therapy.”

(4) The protocol treatment should be discontinued if the criteria shown in Table 8.6.1.1 “Criteria for Continuing FOLFOXIRI Therapy (in the 2nd and subsequent courses)” are not met even after 28 days of the scheduled date of administration.

Table 8.6.1.1 Criteria for Continuing FOLFOXIRI Therapy (in the 2nd and subsequent courses)

Item	Criteria
Neutrophil count	$\geq$1,500/mm^3
Platelet count	$\geq$10.0 × 10^4/mm^3
Hemoglobin	$\geq$9.0 g/dL
Blood bilirubin	$\leq$1.5 times the upper limit of the institutional normal range
AST	$\leq$2.5 times the upper limit of the institutional normal range ($\leq$5 times the upper limit of the institutional normal range for subjects with liver metastasis)
ALT	
Serum creatinine	$\leq$1.5 times the upper limit of the institutional normal range
Infection	Absence of fever $\geq$38.0°C suggestive of infection

Peripheral sensory neuropathy<br>Peripheral motor neuropathy	≤ Grade 1<br>Grade2: FOLFOXIRI + bevacizumab with a 1 dose-level reduction of oxaliplatin<br>Grade3: FOLFIRI + bevacizumab
Other non-hematological findings (excluding constipation, anorexia, nausea, vomiting, alopecia, skin hyperpigmentation, dysgeusia, fatigue, malaise, laboratory abnormalities, and other adverse events considered by the investigator to be non-problematic the initiation of treatment)	≤ Grade 1

*If the start of a course is delayed due to the occurrence of any adverse event that does not fall under the criteria shown above, the course may be started if the adverse event has improved or resolved and the investigator or subinvestigator considers it possible to start the course.

*For a subject receiving a total oxaliplatin dose exceeding 600 mg/m^2, the course may be started with oxaliplatin therapy skipped to ensure the safety of the subject at the discretion of the investigator or subinvestigator.

Table 8.6.1.2 Criteria for Continuing Bevacizumab Therapy

Item	Criteria
Hypertension	Absence of poorly controlled hypertension
Proteinuria	≤ Grade 1
Thromboembolism	Absence of this condition
Bleeding	Absence, or mild bleeding requiring no treatment

FOLFOXIRI + bevacizumab combination therapy will be switched to 5-FU + levofolinate + bevacizumab combination therapy if either of the following is met:

- Up to 12 courses of FOLFOXIRI + bevacizumab combination therapy have been completed.
- A further dose reduction is needed after 2 dose-level reductions of irinotecan and oxaliplatin.

8.6.2 Group B: Criteria for Continuing FOLFOXIRI + Cetuximab Combination Therapy (up to the 12th course)

In Group B, the protocol treatment in the 2nd and subsequent courses should be continued after confirming that the laboratory values and clinical symptoms on the day before or on the starting day of treatment meet the respective criteria shown in both Table 8.6.2.1 “Criteria for Continuing FOLFOXIRI Therapy (in the 2nd and subsequent courses)” and Table 8.6.2.2 “Criteria for Continuing Cetuximab Therapy.”

(1) If the criteria shown in “Table 8.6.2.1 Criteria for Continuing FOLFOXIRI Therapy (in the 2nd and subsequent courses) ” are not meet, but the criteria shown in Table 8.6.2.2 Criteria for Continuing Cetuximab Therapy” are met:

FOLFOXIRI therapy should be delayed and cetuximab therapy alone should be given.
In this case, it is recommended to give the next FOLFOXIRI therapy around 7 days later (around Day 22 of the previous course).

(2) If the criteria shown in Table 8.6.2.1 “Criteria for Continuing FOLFOXIRI Therapy (in the 2nd and

subsequent courses)" are met, but the criteria shown in Table 8.6.2.2 "Criteria for Continuing Cetuximab Therapy" are not met:

FOLFOXIRI therapy alone should be given and cetuximab therapy should be skipped.

(3) Cetuximab therapy may be skipped if the investigator or subinvestigator considers it necessary to do so due to the occurrence of any adverse event that does not fall under the criteria shown in Table 8.6.2.2 "Criteria for Continuing Cetuximab Therapy."

(4) The protocol treatment should be discontinued if the criteria shown in Table 8.6.2.1 "Criteria for Continuing FOLFOXIRI Therapy (in the 2nd and subsequent courses)" are not met even after 28 days of the scheduled date of administration.

Table 8.6.2.1 Criteria for Continuing FOLFOXIRI Therapy (in the 2nd and subsequent courses)

Item	Criteria
Neutrophil count	≥1,500/mm^3
Platelet count	≥10.0 × $10^4/mm^3$
Hemoglobin	≥9.0 g/dL
Blood bilirubin	≤1.5 times the upper limit of the institutional normal range
AST	≤2.5 times the upper limit of the institutional normal range (≤5 times the upper limit of the institutional normal range for subjects with liver metastasis)
ALT	
Serum creatinine	≤1.5 times the upper limit of the institutional normal range
Infection	Absence of fever ≥38.0°C suggestive of infection
Peripheral sensory neuropathy<br>Peripheral motor neuropathy	≤ Grade 1<br>Grade2: FOLFOXIRI + cetuximab with a 1 dose-level reduction of oxaliplatin<br>Grade3: FOLFIRI + cetuximab
Other non-hematological findings (excluding constipation, anorexia, nausea, vomiting, alopecia, skin hyperpigmentation, dysgeusia, fatigue, malaise, laboratory abnormalities, and other adverse events considered by the investigator to be non-problematic the initiation of treatment)	≤ Grade 1

* If the start of a course is delayed due to the occurrence of any adverse event that does not fall under the criteria shown above, the course may be started if the adverse event has improved or resolved and the investigator or subinvestigator considers it possible to start the course.

* For a subject receiving a total oxaliplatin dose exceeding 600 mg/m^2, the course may be started with oxaliplatin therapy skipped to ensure the safety of the subject at the discretion of the investigator or subinvestigator.

Table 8.6.2.2 Criteria for Continuing Cetuximab Therapy

Item	Criteria
Skin symptoms:<br>Acne, rash/desquamation, rash, dry skin, paronychia, pruritus, skin	≤Grade 2

reaction, nail disorder, alopecia, cheilitis, skin disorder, urticaria, hand and foot syndrome, dermatitis exfoliative, dermatitis acneiform, skin fissures, skin toxicity, hair disorder, hirsutism	

*Cetuximab therapy may be delayed if the investigator or subinvestigator considers it necessary to do so due to the occurrence of any adverse event that does not fall under the criteria shown above.

FOLFOXIRI + cetuximab combination therapy will be switched to 5-FU + levofolinate + cetuximab combination therapy if either of the following is met:

- Up to 12 courses of FOLFOXIRI + cetuximab combination therapy have been completed.
- A further dose reduction is needed after 2 dose-level reductions of irinotecan and oxaliplatin.

8.6.3 Criteria for Continuing 5-FU + Levofolinate + Bevacizumab Combination Therapy and 5-FU + Levofolinate + Cetuximab Combination Therapy up to the 12th Course

In Group A, the protocol treatment should be continued after confirming that the laboratory values and clinical symptoms on the day before or on the starting day of treatment meet the respective criteria shown in both Table 8.6.3 “Criteria for Continuing 5-FU + Levofolinate Combination Therapy” and Table 8.6.1.2 “Criteria for Continuing Bevacizumab Therapy.” In Group B, the protocol treatment should be continued after confirming that the laboratory values and clinical symptoms on the day before or on the starting day of treatment meet the respective criteria shown in both Table 8.6.3 “Criteria for Continuing 5-FU + Levofolinate Combination Therapy” and Table 8.6.2.2 “Criteria for Continuing Cetuximab Therapy.”

(1) If the criteria shown in Table 8.6.3 “Criteria for Continuing 5-FU + Levofolinate Combination Therapy” are not met, but the criteria shown in Table 8.6.1.2 “Criteria for Continuing Bevacizumab Therapy” are met in Group A and the criteria shown in Table 8.6.2.2 “Criteria for Continuing Cetuximab Therapy” are met in Group B:

5-FU + levofolinate combination therapy should be delayed, and:

bevacizumab therapy alone should not be given in Group A.

cetuximab therapy alone should be given in Group B.

(2) If the criteria shown in Table 8.6.3 “Criteria for Continuing 5-FU + Levofolinate Combination Therapy” are met, but the criteria shown in Table 8.6.1.2 “Criteria for Continuing Bevacizumab Therapy” are not met in Group A and the criteria shown in Table 8.6.2.2 “Criteria for Continuing Cetuximab Therapy” are not met in Group B:

5-FU + levofolinate combination therapy alone should be given, and bevacizumab therapy in Group A and cetuximab therapy in Group B should be skipped.

(3) In Group A, bevacizumab therapy may be skipped if the investigator or subinvestigator considers it necessary to do so due to the occurrence of any adverse event that does not fall under the criteria shown in Table 8.6.1.2 “Criteria for Continuing Bevacizumab Therapy.”

(4) In Group B, cetuximab therapy may be skipped if the investigator or subinvestigator considers it necessary to do so due to the occurrence of any adverse event that does not fall under the criteria shown in Table 8.6.2.2 “Criteria for Continuing Cetuximab Therapy.”

(5) The protocol treatment should be discontinued if the criteria shown in Table 8.6.3 “Criteria for

Continuing 5-FU + Levofolinate Combination Therapy” are not met even after 28 days of the scheduled date of administration.

Table 8.6.3 Criteria for Continuing 5-FU + Levofolinate Combination Therapy

Item	Criteria
Neutrophil count	≥1,500/mm^3
Platelet count	≥10.0 × 10^4/mm^3
Hemoglobin	≥9.0 g/dL
Blood bilirubin	≤1.5 times the upper limit of the institutional normal range
AST	≤2.5 times the upper limit of the institutional normal range (≤5 times the upper limit of the institutional normal range for subjects with liver metastasis)
ALT	
Serum creatinine	≤1.5 times the upper limit of the institutional normal range
Infection	Absence of fever ≥38.0°C suggestive of infection
Other non-hematological findings (excluding peripheral sensory neuropathy, peripheral motor neuropathy, constipation, anorexia, nausea, vomiting, alopecia, skin hyperpigmentation, dysgeusia, fatigue, malaise, laboratory abnormalities, and other adverse events considered by the investigator to be non-problematic the initiation of treatment)	≤ Grade 1

*If the start of a course is delayed due to the occurrence of any adverse event that does not fall under the criteria shown above, the course may be started if the adverse event has improved or resolved and the investigator or subinvestigator considers it possible to start the course.

8.6.4 Criteria for Continuing 5-FU + Levofolinate + Bevacizumab Combination Therapy and 5-FU + Levofolinate + Cetuximab Combination Therapy in the 13th and Subsequent Courses

In Group A, the protocol treatment should be continued after confirming that the laboratory values and clinical symptoms on the day before or on the starting day of treatment meet the respective criteria shown in both Table 8.6.4 "Criteria for Continuing 5-FU + Levofolinate Combination Therapy" and Table 8.6.1.2 "Criteria for Continuing Bevacizumab Therapy." In Group B, the protocol treatment should be continued after confirming that the laboratory values and clinical symptoms on the day before or on the starting day of treatment meet the respective criteria shown in both Table 8.6.4 "Criteria for Continuing 5-FU + Levofolinate Combination Therapy" and Table 8.6.2.2 "Criteria for Continuing Cetuximab Therapy."

(1) If the criteria shown in Table 8.6.4 "Criteria for Continuing 5-FU + Levofolinate Combination Therapy" are not met, but the criteria shown in Table 8.6.1.2 "Criteria for Continuing Bevacizumab Therapy" are met in Group A and the criteria shown in Table 8.6.2.2 "Criteria for Continuing Cetuximab Therapy" are met in Group B:

5-FU + levofolinate combination therapy should be delayed, and:

bevacizumab therapy alone may be considered if the investigator or subinvestigator considers it appropriate in Group A.

cetuximab therapy alone should be given in Group B.

(2) If the criteria shown in Table 8.6.4 "Criteria for Continuing 5-FU + Levofolinate Combination

Therapy" are met, but the criteria shown in Table 8.6.1.2 "Criteria for Continuing Bevacizumab Therapy" are not met in Group A and the criteria shown in Table 8.6.2.2 Criteria for Continuing Cetuximab Therapy" are not met in Group B:

5-FU + levofolinate combination therapy alone should be given, and bevacizumab therapy in Group A and cetuximab therapy in Group B should be skipped.

(3) In Group A, bevacizumab therapy may be skipped if the investigator or subinvestigator considers it necessary to do so due to the occurrence of any adverse event that does not fall under the criteria shown in Table 8.6.1.2 "Criteria for Continuing Bevacizumab Therapy."

(4) In Group B, cetuximab therapy may be skipped if the investigator or subinvestigator considers it necessary to do so due to the occurrence of any adverse event that does not fall under the criteria shown in Table 8.6.2.2 "Criteria for Continuing Cetuximab Therapy."

(5) The protocol treatment should be discontinued if the criteria shown in neither Table 8.6.4 "Criteria for Continuing 5-FU + Levofolinate Combination Therapy" nor Table 8.6.1.2 "Criteria for Continuing Bevacizumab Therapy" are met in Group A and the criteria shown in neither Table 8.6.4 "Criteria for Continuing 5-FU + Levofolinate Combination Therapy" nor Table 8.6.2.2 "Criteria for Continuing Cetuximab Therapy" are met in Group B even after 28 days of the scheduled date of administration.

Table 8.6.4 Criteria for Continuing 5-FU + Levofolinate Combination Therapy

Item	Criteria
Neutrophil count	≥1,500/mm^3
Platelet count	≥10.0 × 10^4/mm^3
Hemoglobin	≥9.0 g/dL
Blood bilirubin	≤1.5 times the upper limit of the institutional normal range
AST	≤2.5 times the upper limit of the institutional normal range (≤5 times the upper limit of the institutional normal range for subjects with liver metastasis)
ALT	
Serum creatinine	≤1.5 times the upper limit of the institutional normal range
Infection	Absence of fever ≥38.0°C suggestive of infection
Other non-hematological findings (excluding peripheral sensory neuropathy, peripheral motor neuropathy, constipation, anorexia, nausea, vomiting, alopecia, skin hyperpigmentation, dysgeusia, fatigue, malaise, laboratory abnormalities, and other adverse events considered by the investigator to be non-problematic the initiation of treatment)	≤ Grade 1

*If the start of a course is delayed due to the occurrence of any adverse event that does not fall under the criteria shown above, the course may be started if the adverse event has improved or resolved and the investigator or subinvestigator considers it possible to start the course.

8.7 Criteria for Dose Reduction (in the 2nd and subsequent courses)

8.7.1 Criteria for Dose Reduction of FOLFOXIRI

(1) If any of the criteria shown in Table 8.7.1.1 “Criteria for Dose Reduction of FOLFOXIRI Combination Therapy” is met during FOLFOXIRI combination therapy, after confirming that the criteria shown in Table 8.6.1.1 “Criteria for Continuing FOLFOXIRI Therapy (in the 2nd and subsequent courses)” (same as Table 8.6.2.1) are met, the protocol treatment should be given with a 1 dose-level reduction of 5-FU, irinotecan, or oxaliplatin according to the criteria shown in Table 8.7.1.1 “Criteria for Dose Reduction of FOLFOXIRI Combination Therapy.”

(2) The dose may be also reduced if the investigator or subinvestigator considers it necessary to do so due to the occurrence of any adverse event that does not fall under the criteria shown in Table 8.7.1.1 “Criteria for Dose Reduction of FOLFOXIRI Combination Therapy.”

(3) The reduced doses of individual drugs are shown in Table 8.7.1.2 “Initial Recommended Doses and Reduced Doses of FOLFOXIRI Combination Therapy.”

(4) Any reduced dose of each drug must not be increased again.

Table 8.7.1.1 Criteria for Dose Reduction of FOLFOXIRI Combination Therapy

<table>
<tr><th>Item</th><th>5-FU</th><th>Irinotecan</th><th>Oxaliplatin</th></tr>
<tr><td>Grade 4 neutropenia
(neutrophil count $<500/mm^3$)</td><td rowspan="5">No reduction</td><td rowspan="5">1 dose-level reduction</td><td rowspan="5">1 dose-level reduction</td></tr>
<tr><td>≥Grade 3 febrile neutropenia</td></tr>
<tr><td>≥Grade 3 thrombocytopenia
(platelet count $<5.0 \times 10^4/mm^3$)</td></tr>
<tr><td>≥Grade 3 non-hematological toxicity (excluding diarrhea, nausea, vomiting, oral mucositis, fatigue, peripheral sensory neuropathy, and peripheral motor neuropathy)</td></tr>
<tr><td>Dose delay for ≥14 days due to adverse events</td></tr>
<tr><td>≥Grade 3 oral mucositis</td><td>1 dose-level reduction</td><td>No reduction</td><td>No reduction</td></tr>
<tr><td>2nd dose interruption due to grade 2 diarrhea or ≥Grade 3 diarrhea</td><td>No reduction</td><td>1 dose-level reduction</td><td>No reduction</td></tr>
<tr><td>≥Grade 2 peripheral sensory neuropathy or peripheral motor neuropathy</td><td>No reduction</td><td>No reduction</td><td>1 dose-level reduction</td></tr>
</table>

Table 8.7.1.2 Initial Recommended Doses and Reduced Doses of FOLFOXIRI Combination Therapy

Dose	5-FU	Irinotecan	Oxaliplatin
Initial recommended dose	2400 mg/m^2	150 mg/m^2	85 mg/m^2
After 1 dose-level reduction	2000 mg/m^2	125 mg/m^2	65 mg /m^2
After 2 dose-level reduction	1600 mg/m^2	100 mg/m^2	50 mg/m^2

A chart of the criteria for dose reduction of FOLFOXIRI combination therapy is shown in the next page.

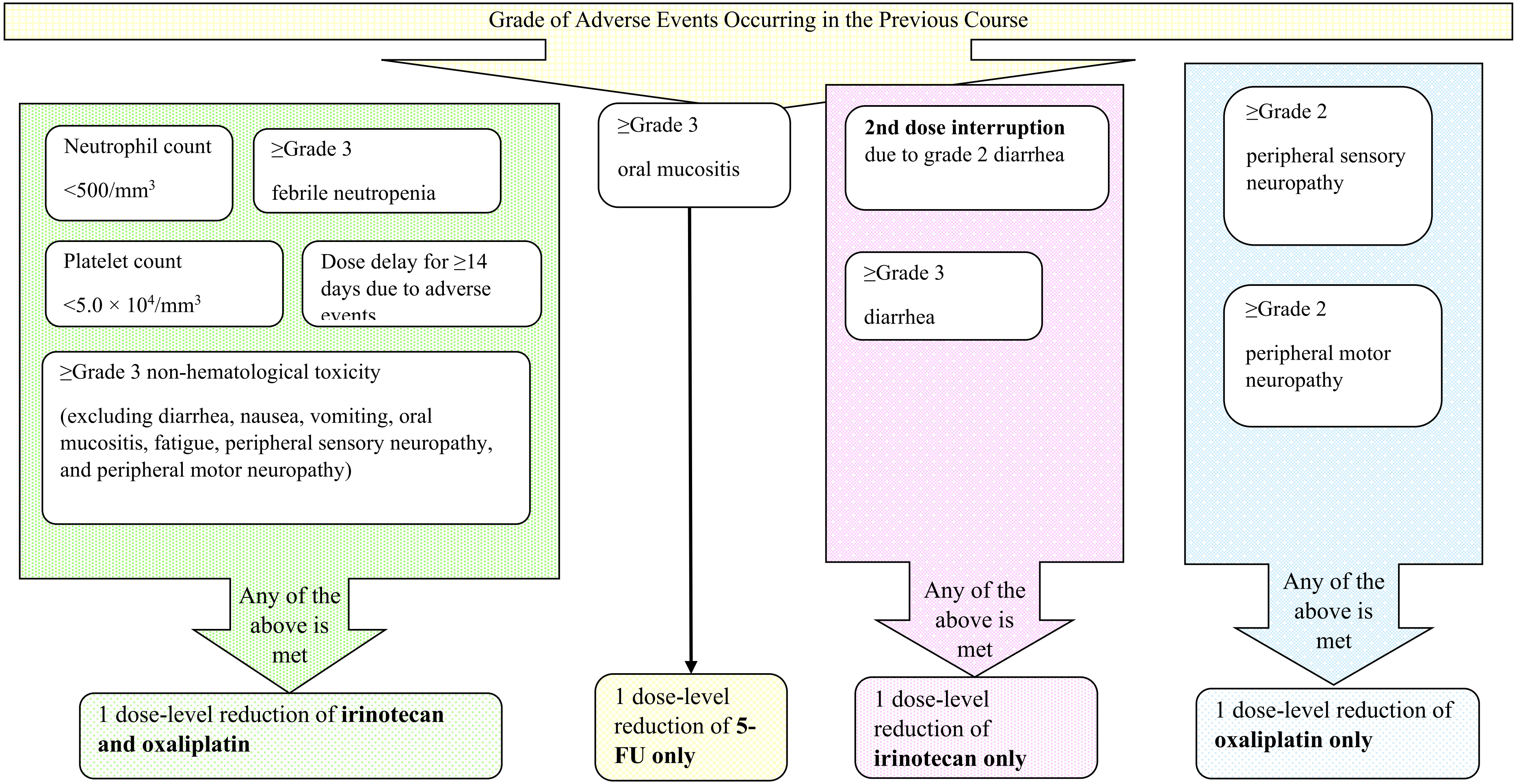

Figure 8.7.1.3 Criteria for Dose Reduction of FOLFOXIRI Combination Therapy (Chart based on Table 8.7.1.1)

8.7.2 Criteria for Dose Reduction of Bevacizumab

The dose of bevacizumab will not be reduced.

8.7.3 Criteria for Dose Reduction of Cetuximab

(1) If any dose is delayed due to the occurrence of an adverse event during the protocol treatment, after the criteria shown in Table 8.6.2.2 "Criteria for Continuing Cetuximab Therapy" are met, the dose of cetuximab should be reduced by one dose level according to the criteria shown in Table 8.7.3.1 "Criteria for Dose Reduction of Cetuximab."

(2) The reduced doses of cetuximab are shown in Table 8.7.3.2 "Initial Recommended Doses and Reduced Doses of Cetuximab."

(3) If further dose reduction is needed after a 1 dose-level reduction, the dose of cetuximab should be reduced by another dose level. However, if any clinically significant adverse event occurs, the next dose of cetuximab may be reduced by two dose levels at a time at the discretion of the investigator or subinvestigator.

(4) If any adverse event that requires further dose reduction occurs in a subject whose cetuximab dose has been reduced by two dose levels, administration of cetuximab should be discontinued with no further dose reduction.

(5) The dose may be also reduced if the investigator or subinvestigator considers it necessary to do so due to the occurrence of any adverse event that does not fall under the criteria shown in Table 8.7.3.1 "Criteria for Dose Reduction of Cetuximab."

(6) Any reduced dose of cetuximab must not be increased again.

Table 8.7.3.1 Criteria for Dose Reduction of Cetuximab

Item	Cetuximab
Grade 3 skin symptom	1 dose-level reduction

Table 8.7.3.2 Standard Doses and Reduced Doses of Cetuximab

Dose	Cetuximab
Standard dose	250 mg/m^2
1 dose-level reduction	200 mg/m^2
2 dose-levels reduction	150 mg/m^2

8.7.4 Criteria for Dose Reduction of 5-FU + Levofolinate Combination Therapy

(1) If the severity of an adverse event meets any of the criteria shown in Table 8.7.4.1 "Criteria for Dose Reduction of 5-FU + Levofolinate Combination Therapy" during 5-FU + levofolinate combination therapy, after confirming that the criteria shown in Table 8.6.3 "Criteria for Continuing 5-FU + Levofolinate Combination Therapy" (same as Table 8.6.4) are met, the dose of 5-FU should be reduced by one dose level according to the criteria shown in

Table 8.7.4.1 “Criteria for Dose Reduction of 5-FU + Levofolinate Combination Therapy.”

(2) The reduced doses of 5-FU are shown in Table 8.7.4.2 “Initial Recommended Doses and Reduced Doses of 5-FU.”

(3) Any reduced dose of 5-FU must not be increased again.

Table 8.7.4.1 Criteria for Dose Reduction of 5-FU + Levofolinate Combination Therapy

Item	5-FU
Grade 4 neutropenia (neutrophil count <500/mm^3)	1 dose-level reduction
≥Grade 3 febrile neutropenia	
≥Grade 3 thrombocytopenia (platelet count <5.0 × 10^4/mm^3)	
≥Grade 3 non-hematological toxicity (excluding diarrhea, nausea, and vomiting)	
Dose delay for ≥14 days due to toxicities	
2nd dose interruption due to grade 2 diarrhea or ≥Grade 3 diarrhea	

Table 8.7.4.2 Initial Recommended Doses and Reduced Doses of 5-FU

Dose	5-FU
Initial recommended dose	2400 mg/m^2
1 dose-level reduction	2000 mg/m^2
2 dose-levels reduction	1600 mg/m^2

8.8 Criteria for Discontinuation of 5-FU, Irinotecan, Oxaliplatin, Bevacizumab, and Cetuximab

(1) Criteria for discontinuation of 5-FU

If further dose reduction is needed after 2 dose-level reductions of 5-FU during the protocol treatment:

Up to the 12th course: The protocol treatment should be discontinued.

In the 13 and subsequent courses:

bevacizumab therapy alone may be considered if the investigator or subinvestigator considers it appropriate in Group A.

cetuximab therapy alone should be given in Group B.

(2) Criteria for discontinuation of irinotecan

If further dose reduction is needed after 2 dose-level reductions of irinotecan, irinotecan should be discontinued. After discontinuation, irinotecan must not be resumed even if the adverse event has improved or resolved. If irinotecan alone is discontinued, administration of 5-FU, oxaliplatin, bevacizumab, and cetuximab should be continued.

(3) Criteria for discontinuation of oxaliplatin

If further dose reduction is needed after 2 dose-level reductions of oxaliplatin, oxaliplatin should be discontinued. After discontinuation, oxaliplatin must not be resumed even if the adverse event has improved or

resolved. If oxaliplatin alone is discontinued, administration of 5-FU, irinotecan, bevacizumab, and cetuximab should be continued.

*A further dose reduction is needed after 2 dose-level reductions of both irinotecan and oxaliplatin, irinotecan and oxaliplatin should be discontinued, and the therapy should be switched to 5-FU + levofolinate + bevacizumab combination therapy in Group A and to 5-FU + levofolinate + cetuximab combination therapy in Group B.

(4) Criteria for discontinuation of bevacizumab

If any of the criteria in Table 8.8 "Criteria for Discontinuation of Bevacizumab" is met in Group A, bevacizumab should be discontinued. After discontinuation, bevacizumab must not be resumed even if the adverse event has improved or resolved. If only the criteria for discontinuation of bevacizumab are met, FOLFOXIRI therapy should be continued (in the case of 5-FU + levofolinate + bevacizumab combination therapy, 5-FU + levofolinate should be continued).

Table 8.8 Criteria for Discontinuation of Bevacizumab

Item	Criteria
Proteinuria	Grade 3
Thromboembolism	≥Grade 3
Gastrointestinal tract perforation or paracentesis	Presence of this condition
Hemoptysis (pulmonary hemorrhage)	Presence of this condition
Bleeding	Severe bleeding
Hypertension	Hypertension not controlled with drugs
Hypersensitivity	≥Grade 3
Reversible posterior leukoencephalopathy syndrome	Presence of this condition

(5) Criteria for discontinuation of cetuximab

In Group B, if any adverse event that requires further dose reduction occurs in a subject whose cetuximab dose has been reduced by two dose levels, administration of cetuximab should be discontinued with no further dose reduction. After discontinuation, cetuximab must not be resumed even if the adverse event has improved or resolved. If only the criteria for discontinuation of cetuximab are met, FOLFOXIRI therapy should be continued (in the case of 5-FU + levofolinate + cetuximab combination therapy, 5-FU + levofolinate should be continued).

8.9 Dose Increase

Any reduced dose of 5-FU, irinotecan, oxaliplatin, bevacizumab, or cetuximab must not be increased again.

9 Concomitant Therapies

9.1 Prohibited Concomitant Therapies

Any cancer therapy, other than the study drugs used in this study, will be prohibited throughout the study period. More specifically, prohibited concomitant therapies include chemotherapy drugs excluding 5-FU, irinotecan, oxaliplatin, bevacizumab, and cetuximab; immunotherapy (BRM); antibody therapy; thermotherapy; radiotherapy; surgical therapy; and other therapies possibly affect the evaluation in this study. Concomitant use of any investigational product (including those other than anticancer drugs) will be also prohibited.

9.2 Permitted Concomitant Drugs

Concomitant use of the following drugs or therapies that would not affect the efficacy evaluation will be permitted.

(1) Prophylaxis with 5-HT_3 receptor antagonists (e.g., Kytril, Palonosetron, Serotone, Zofran, Sinseron), NK1 receptor antagonists (e.g., Aprepitant), and steroids for the relief of nausea and vomiting

(2) Prophylaxis with Hangeshashinto, Saireito, intestinal alkalization, or other measures against diarrhea

(3) G-CSF preparations (e.g., Neutrogin, Neu-up, Gran) that will be used for the indications below; except for the use to assure subject safety in the opinion of the investigator or subinvestigator:

1) Grade 4 neutropenia (<500/mm^3)

2) Grade 3 neutropenia (<1,000/mm^3) with fever (≥38.0°C)

3) Grade 3 neutropenia (<1,000/mm^3) after the use of any G-CSF preparation for the reasons 1) and/or 2)

(4) Steroids to treat allergic reaction or prophylaxis with steroids against allergic reaction

(5) Opioid preparations such as morphine, oxycodone, and fentanyl patches

(6) Use of any other drugs for the symptomatic treatment of complications or adverse events

9.3 Contraindications for Coadministration and Precautions for Coadministration

(1) 5-FU

Contraindications for coadministration: Combination drug of tegafur, gimeracil, and oteracil potassium (TS-1)

Precautions for coadministration: phenytoin, warfarin potassium, other chemotherapy drugs, radiation therapy

(2) Irinotecan

Contraindications for coadministration: atazanavir sulfate (REYATAZ)

Precautions for coadministration: Other antineoplastic drugs, radiation irradiation, peripheral muscle relaxants, CYP3A4 inhibitors, CYP3A4 inducers, sorafenib tosilate, lapatinib tosilate hydrate, regorafenib hydrate

(3) Oxaliplatin

Contraindications for coadministration: None

Precautions for coadministration: Other antineoplastic drugs, radiation irradiation

(4) Bevacizumab

Contraindications for coadministration: None

Precautions for coadministration: None

(5) Cetuximab

Contraindications for coadministration: None

Precautions for coadministration: None

9.4 Recommended Supportive Therapy

Premedication including prophylactic antiemetics

At 30 minutes before administration of irinotecan (on Day 1):

5-HT_3 receptor antagonists + dexamethasone at 6.6 to 13.2 mg ± NK1 receptor antagonists

On Days 2 to 4:

Dexamethasone tablets 0.5 mg at 4 to 8 mg divided into two doses (in the morning and at noon)

10 Criteria for Discontinuation of the Protocol Treatment for Individual Subjects

The protocol treatment will be discontinued in any of the following cases. The date of discontinuation when the protocol treatment is discontinued is defined as not the day of an event that causes the discontinuation but the day of discontinuation determined by the investigator.

(1) The subject cannot start the protocol treatment (FOLFOXIRI + bevacizumab combination therapy or FOLFOXIRI + cetuximab combination therapy) within 14 days of registration.

(2) The subject has progressive disease (PD) after the start of the protocol treatment.

(3) The subject cannot start the protocol treatment even after 28 days of the scheduled date of administration in the next course (except for administration on the same day of the week after 4 weeks).

(4) The subject needs further dose reduction after 2 dose-level reductions of 5-FU during FOLFOXIRI combination therapy or 5-FU + levofolinate combination therapy up to the 12th course.

(5) The subject requests discontinuation of the protocol treatment.

(6) The subject dies (the date of discontinuation is the day of death in this case).

(7) The subject withdraws consent.

(8) The subject is found to have any reason for exclusion from the study after registration.

(9) The subject is found to be pregnant after registration.

(10) The subject cannot continue the protocol treatment due to adverse events in the opinion of the investigator or subinvestigator.

(11) Other cases where it is inappropriate for the subject to continue the protocol treatment in the opinion of the investigator or subinvestigator.

11 Follow-up of Discontinued Subjects

Discontinued subjects will be followed up as described below according to the reason for discontinuation. Discontinued subjects will be also included in the evaluation as with subjects continuing the study.

(1) PD [Discontinuation criterion (2)]:

Observation of the outcome of the subject will be continued for 3 years after registration of the last subject.

(2) Request of the subject [Discontinuation criterion (5)]:

Evaluation of the antitumor effect as well as observation of progression and the outcome of the subject will be continued for 3 years after registration of the last subject in the cases of the subject's refusal to continue the protocol treatment and discontinuation of the protocol treatment because of the subject's request for an alternative to the protocol treatment.

(3) Failure to start the protocol treatment within 14 days of registration; consent withdrawal of the subject; death of the subject; being ineligible after registration [Discontinuation criteria (1), (6), (7), (8), and (9)]:

No follow-up (e.g., observation of progression and the outcome of the subject) will be performed.

(4) Other reasons [Discontinuation criteria (3), (4), (10), and (11)]:

Evaluation of the antitumor effect as well as observation of progression and the outcome of the subject will be continued for 3 years after registration of the last subject. Even if it is impossible to observe progression or the outcome of the subject as specified in the protocol for hospital transfer or other reasons, information on the outcome of the subject will be collected from the hospital where the subject has been transfered whenever possible, and the method and date of collection will be recorded on the medical chart.

12 Subsequent Treatment (Secondary Treatment)

Although no particular subsequent treatment after discontinuation of the protocol treatment is prespecified in the protocol, a follow-up report on any subsequent treatment given should be submitted.

Surgical resection may be indicated if the disease has become resectable after the protocol treatment.

In such cases, resection of the disease should be performed after a sufficient period of time (at least 28 days) following the last dose of bevacizumab in Group A. No subsequent treatment after recection is also prespecified in the protocol.

13 Anticipated Adverse Drug Reactions

13.1 Adverse Drug Reactions Expected with Individual Drugs

For adverse drug reactions expected with individual drugs, refer to the latest package inserts of the following drugs:

- 5-FU (See Attachment (1))
- Irinotecan (See Attachment (2))
- Oxaliplatin (See Attachment (3))
- Levofolinate (See Attachment (4))
- Bevacizumab (See Attachment (5))
- Cetuximab (See Attachment (6))

13.2 Adverse Reactions Expected with Combination Chemotherapy, Chemoradiotherapy, and Combined Modality Therapy

Table 13.2.1 Adverse Events with FOLFOXIRI + Bevacizumab Combination Therapy

≥Grade 3	Phase II study of FOLFOXIRI + bevacizumab[20] (n=57)	TRIBE study of FOLFOXIRI + bevacizumab[6] (n=250)
Nausea	4%	3%
Vomiting	0%	4%
Diarrhea	14%	19%
Stomatitis	4%	9%
Neutropenia	50%	50%
Febrile neutropenia	2%	9%
Peripheral neuropathy	2%	5%
Hypertension	11%	5%
Venous thrombosis	7%	7%
Arterial thrombosis	0%	1%
Bleeding	0%	1%

Table 13.2.2 Adverse Events with mFOLFOXIRI + Cetuximab Combination Therapy

≥Grade 3	MACBETH study of FOLFOXIRI + cetuximab[21] (n=72)
Diarrhea	21.3%
Stomatitis	6.7%
Nausea	1.3%
Vomiting	2.7%
Neutropenia	34.7%
Febrile neutropenia	2.7%
Peripheral neuropathy	2.7%
Skin eruption	14.7%

14 Expected Duration of Subject's Participation in the Study

For at least 3 years, up to 5 years, after registration

15 Tests, Observations, and Examinations and Timing

15.1 Subject Characteristics

The following subject characteristics will be examined or confirmed by the time of registration:

(1) Sex

(2) Age at informed consent

(3) Date of informed consent

(4) Body height

(5) Body weight (within 14 days before registration)

(6) Clinical diagnosis and date of initial/recurrent cancer

(7) History of prior treatment for the primary disease and its contents

(8) History of postoperative adjuvant chemotherapy and its contents

(9) Presence/absence of primary tumors

(10) Primary tumor site

(11) Histopathological classification

(12) Genetic testing

Tumor: *RAS* mutations, *BRAF* mutations

Blood: *UGT1A1* polymorphisms *28 and *6 (to be recorded if any; testing is recommended)

(13) Metastatic sites

(14) ECOG PS (within 14 days before registration)

15.2 Tests, Observations, and Examinations

(1) Hematology: White blood cell count, neutrophil count, lymphocyte count, hemoglobin, platelet count

(2) Biochemistry: Albumin, AST, ALT, ALP, LDH, blood bilirubin, serum creatinine, Ca, Na, K, Mg

(3) Coagulation and fibrinolytic systems: PT-INR

(4) Urinalysis: Proteinuria

(5) Blood pressure

(6) Clinical symptoms (subjective/objective symptoms): to be evaluated using CTCAE v4.0-JCOG adverse event items

(7) Tumor markers: CEA, CA19-9

(8) Diagnostic imaging: CT/MRI will be used to measure/evaluate target lesions and evaluate non-target lesions.

(9) Biomarkers (only in subjects participating in the JACCRO CC-13AR study)

15.3 Follow-up

Each subject will be followed up every 6 months. The follow-up study will be continued for 3 years after registration of the last subject.

15.4 Observations and Examinations and Schedule

Table 15.4 Observations and Examinations and Schedule

	At registration	In each course		At discontinuation
	Within 14 days before registration	On Day 1 or the previous day	On Day 8 or the previous day	
Subject characteristics	⊙			
Hematology	⊙	⊙	○	⊙
Biochemistry	⊙	⊙	○	⊙
Coagulation and fibrinolytic systems	⊙	⊙		⊙
Urinalysis	⊙	⊙		⊙
Blood pressure	⊙	⊙	○	⊙
Clinical symptoms	⊙	⊙	○	⊙
General findings (PS)	⊙	⊙	○	⊙
Tumor markers	⊙	To be tested at least once every 8 weeks		⊙
Diagnostic imaging	⊙	To be performed at least once every 8 weeks<br>Any subject with PR-in should be confirmed 4 weeks later.		⊙

⊙: Required, ○: Only in the 1st course whenever possible

16 Endpoints and Evaluation Criteria

16.1 Endpoints

16.1.1 Primary Endpoint

Deepness of response (DpR)*

*Deepness of response (DpR) is defined as the sum of the longest diameters of RECIST target lesions at the nadir in the absence of progression subtracted from the sum of the longest diameters of RECIST target lesions at baseline divided by the sum of the longest diameters of RECIST target lesions at baseline.

16.1.2 Secondary Endpoints

Early tumor shrinkage (ETS) at Week 8

Response rate (RR)

Deepness of response (DpR) by Month 4

Time to treatment failure (TTF)
Time to tumor growth (TTG)
Progression-free survival (PFS)
Overall survival (OS)
Association between tumor shrinkage (ETS, RR, DpR) and prognosis (PFS, OS)
Association between TTG and prognosis (OS)
Resection rate
R0 resection rate
Safety (incidence and severity of adverse events)

16.2 Definitions/Criteria of the Endpoints

16.2.1 Deepness of response (DpR)

The deepness of response in each subject is defined as the sum of the longest diameters of RECIST target lesions at the nadir in the absence of progression subtracted from the sum of the longest diameters of RECIST target lesions at baseline divided by the sum of the longest diameters of RECIST target lesions at baseline.

16.2.2 Early Tumor Shrinkage (ETS)

Early tumor shrinkage is defined as the percentage of subjects achieving tumor shrinkage by ≥20% in each group analyzed at the time of the initial response evaluation.

16.2.3 Response Rate

Response rate is defined as the percentage of subjects with an overall response of either CR or PR in each group analyzed. Response will be also evaluated externally by the Response Review Committee.

16.2.4 Time to Treatment Failure (TTF)

Time to treatment failure is defined as the time interval from the date of registration to the date of diagnosis of progressive disease (PD), the date of death due to any cause, or the date of discontinuation of the protocol treatment, whichever comes first. Progression (clinical progression) of the primary disease that cannot be confirmed by diagnostic imaging will be regarded as PD.

(1) For a subject on the protocol treatment, the date of censoring will be the date of the final confirmation that the subject is on treatment (the date of the final outpatient visit during the treatment period).

(2) For a subject responding to the protocol treatment and undergoing surgery based on the indication for surgical resection of the primary tumor or metastasis, the date of censoring will be the date of surgery performed in the subject.

16.2.5 Time to Tumor Growth (TTG)

Time to tumor growth is defined as the time interval from the date of registration to the date of confirmation of the smallest sum of the diameters of target lesions in the absence of new lesions or progressive disease (PD) of non-target lesions.

Progression (clinical progression) of the primary disease that cannot be confirmed by diagnostic imaging will be regarded as PD. Any subject responding to the protocol treatment and undergoing surgery based on the indication for surgical resection of the primary tumor or metastasis will be regarded as a censored case on the date of surgery.

16.2.6 Progression-free Survival (PFS)

Progression-free survival is defined as the time interval from the date of registration to the date of diagnosis of progressive disease (PD) or the date of death due to any cause, whichever comes first. Progression (clinical progression) of the primary disease that cannot be confirmed by diagnostic imaging will be regarded as PD. Any subject responding to the protocol treatment and undergoing surgery based on the indication for surgical resection of the primary tumor or metastasis will be regarded as a censored case on the date of surgery.

16.2.7 Overall Survival (OS)

Overall survival is defined as the time interval from the date of registration to the date of death due to any cause. For a survivor, the date of censoring will be the date of the final confirmation of survival of the subject. For a loss to follow-up, the date of censoring will be the date of the final confirmation of survival of the subject before being lost to follow-up.

16.2.8 Safety

The frequency of individual adverse events (toxicities) of the highest grade according to the CTCAE version 4.0-JCOG across the courses in the safety analysis set will be tabulated.

17 Submission of Reports

The investigator or subinvestigator will enter information required for each type of report into the FLADS® system.

(1) Registration report: Registration of a new subject will be entered into the FLADS® system.

(2) Subject characteristics report: Information on “subject characteristics” will be entered into the FLADS® system before the start of the protocol treatment.

(3) Drug administration report: The implementation status of the protocol treatment will be entered into the FLADS® system.

(4) Laboratory test results: Copies of laboratory test results will be sent the JACCRO Study Secretariat by mail.

(5) Adverse event report: The occurrence of adverse events will be entered into the FLADS® system.

(6) Response evaluation report: Information on the target lesions, non-target lesions, new lesions, overall evaluation, etc. will be entered into the FLADS® system.

(7) Protocol treatment discontinuation report: Information on discontinuation of the protocol treatment will be entered into the FLADS® system.

(8) Follow-up report: Confirmation results of outcome and use of subsequent treatment and its contents will be entered into the FLADS® system (every 6 months).

(9) Serious adverse event report: At the occurrence of a serious adverse event, necessary information will be entered into the serious adverse event form on the FLADS® system. Any site-specific form may be used for reporting.

Serious adverse event reports may be also sent to the JACCRO Study Secretariat via fax or e-mail.
An initial report, which includes information collected within the reporting timeline for expedited reporting or ordinary reporting (see 18.4 "Procedures for Reporting and Handling of Serious Adverse Events"), will be sent to the JACCRO Study Secretariat via fax or e-mail.
If detailed information is obtained later, a follow-up report, which includes the additional information, will be sent to the JACCRO Study Secretariat via fax or e-mail.

18 Reporting of Adverse Events

18.1 Definitions of Terms

(1) Adverse event

An adverse event is any unfavorable and unintended sign (including an abnormal laboratory finding), symptom, or disease observed with the use of a medical treatment or procedure during a clinical study, whether or not related to the medical treatment or procedure. Adverse events therefore include both those "considered related" and those "not considered related."

(2) Serious adverse event

A serious adverse event is defined as any adverse event that:

1) results in death (death)
2) is life-threatening*1 (is at risk of death)
3) requires inpatient hospitalization or prolongation of existing hospitalization for treatment*2
4) results in persistent or significant disability/incapacity
5) is a congenital anomaly/birth defect, or
6) is a medically important event or reaction.

*1: The term "life-threatening" in the definition of "serious" refers to an event in which the subject was at risk of death at the time of the event; it does not refer to an event which hypothetically might have caused death if it were more severe.

*2: Hospitalization of which purpose is other than treatment for an adverse event, such as hospitalization for examination to confirm recurrence, is not defined as a serious adverse event.

(3) Causal relationship

To determine whether an adverse event is subject to expedited reporting or not, it is necessary to determine whether a causal relationship between the adverse event and the protocol treatment is a reasonable possibility. In this study, according to the United States National Cancer Institute (NCI) guidelines (NCI GUIDELINES FOR INVESTIGATORS: ADVERSE EVENT REPORTING REQUIREMENTS FOR DCTD (CTEP AND CIP) AND DCP INDs AND IDEs), the causal relationship of individual adverse events will be classified into the following categories:

1) Definite:

It is evident that the adverse event has been caused or worsened by the protocol treatment, and it is considered less likely that it has been caused or worsened by the progression of the primary disease or other factors

(comorbidities, other drugs/treatments, accidental symptoms).

2) Probable:

It is less likely that the adverse event has been caused or worsened by the progression of the primary disease or other factors (comorbidities, other drugs/treatments, accidental symptoms), and it is considered more plausible that it has been caused or worsened by the protocol treatment.

3) Possible:

It is difficult to determine whether the adverse event has been caused or worsened by the protocol treatment or by the progression of the primary disease or other factors (comorbidities, other drugs/treatments, accidental symptoms).

4) Unlikely:

It is less likely that the adverse event has been caused or worsened by the protocol treatment, and it is considered more plausible that it has been caused or worsened by the progression of the primary disease or other factors (comorbidities, other drugs/treatments, accidental symptoms).

5) Not related:

It is evident that the adverse event has been caused or worsened by the progression of the primary disease or other factors (comorbidities, other drugs/treatments, accidental symptoms), and it is considered less likely that it has been caused or worsened by the protocol treatment.

In this study, adverse events categorized as definite, probable, or possible will be considered as "related" and those categorized as unlikely or not related will be considered as "unrelated."

(4) Expectedness (expected/unexpected)

An expected adverse event refers to that listed as an adverse event that may occur in this study in a package insert, a protocol, or other documents. However, although listed in such documents, any adverse event of which nature, severity, specificity, or outcome is not consistent with information in the relevant documents is considered to be an unexpected adverse event.

18.2 Types of Adverse Events Requiring Reporting（After the certified review board approval）

(1) Adverse events requiring expedited reporting

1) Deaths or life-threatening adverse events during the protocol treatment or within 30 days of discontinuation of the protocol treatment

2)

Other possibly related unexpected serious adverse events

(2) Adverse events requiring ordinary reporting

Alladverse events other than those requiring expedited reporting(however other than those occurring after 30 days of the last dose of a drug as the protocol treatment)

*: The number of days after discontinuation of the protocol treatment will be counted from the "date of the last dose of a drug included in the protocol treatment."

18.3 Time Frames for Reporting by the Investigator or Subinvestigator（After the certified review board approval）

(1) Expedited reporting

Initial report: within 24 hours of the investigator or subinvestigator becoming aware of the event

Follow-up (detailed) report: within 7 days of the investigator or subinvestigator becoming aware of the event

(2) Ordinary reporting

Initial report: within 7 days of the investigator or subinvestigator becoming aware of the event

Follow-up (detailed) report: within 14 days of the investigator or subinvestigator becoming aware of the event

18.4 Procedures for Reporting and Responding to Serious Adverse Events（After the certified review board approval）

18.4.1 Expedited Reporting（After the certified review board approval）

(1) Reporting by the investigator or subinvestigator

1) If becoming aware of any serious adverse event requiring expedited reporting, the investigator or subinvestigator should enter necessary information into the serious adverse event form on the FLADS® system and report it to the Principal Investigator and the manager of the medical institution of the investigator or subinvestigator as an initial report within 24 hours of becoming aware of the event. However, reporting to the managerof the medical institution should be in accordance with the procedure at each medical institution (the same hereinafter).

2) The investigator or subinvestigator should also report the serious adverse event to the Principal Investigator and the manager of the medical institution as a follow-up report via the serious adverse event form on the FLADS® system within 7 days of becoming aware of the event. If requested by the Principal Investigator to report detailed information that is not included in the follow-up report, the investigator or subinvestigator should conduct a necessary and thorough investigation as directed, and report the results of the investigation to the Principal Investigator.

(2) Response of the Principal Investigator

The Principal Investigator should thoroughly investigate the reported serious adverse event, including its causal relationship with the protocol treatment and expectedness, and respond regarding the following.

1) Deaths for which a causal relationship with a study drug cannot be ruled out , Possibly related and unexpected serious adverse events(other than death)

i) Report to the medical institution , the certified review board ,Minister of Health, Labour and Welfare

• The Principal Investigator should also report the investigator or subinvestigator reports the serious adverse event as a follow-up report to the managers of all the medical institutions.

• Within 15 days of the initial report,the Principal Investigator should also report the investigator or subinvestigator reports the serious adverse event as a follow-up report to the certified review board.

• When received reccomendation other than the study continuation from the certified review board, the Principal Investigator should decide of details of the need and measures,and report it to all Investigators.

• The Principal Investigator should also report the investigator or subinvestigator reports the serious adverse event as a follow-up report to Minister of Health, Labour and Welfare.

ii) Request evaluations to the certified review board

• The Principal Investigator should send and request the evalution the investigator or subinvestigator reports the serious adverse event as a follow-up report to the Data and Safety Monitoring Committee.However, if considering that emergency action is required and there is no time to wait for evaluation of the event by the Data and Safety Monitoring Committee, the Principal Investigator him/herself should determine what emergency action should be taken, inform the investigator at each participating medical institution of the action and the reason for it, and immediately report the response of the Principal Investigator to the Data and Safety Monitoring Committee for approval.

<<Evaluations and recommendations by the Data and Safety Monitoring Committee>>

• The Data and Safety Monitoring Committee should examine the follow-up report, and request the Principal Investigator for a further investigation if requiring additional detailed information.

• The Principal Investigator should request the investigator or subinvestigator for an additional further investigation based on the instructions.

• After being confirmed by the Principal Investigator, results of additional further investigation sent by the investigator or subinvestigator should be reported to the Data and Safety Monitoring Committee.

• The Data and Safety Monitoring Committee should evaluate the serious adverse event based on all of the submitted information, and submit a written recommendation on the need to take action for the serious adverse event and details of the action to the Principal Investigator.

• When received reccomendation other than the study continuation from the Data and Safety Monitoring Committee, the Principal Investigator should decide of details of the need and measures,and report it to all Investigators.

2）For life-threatening adverse events for which a causal relationship with a study drug cannot be ruled out,but expected

i) Report to the medical institution , the certified review board ,Minister of Health, Labour and Welfare. The Principal Investigator should also report the investigator or subinvestigator reports the serious adverse event as a follow-up report to the managers of all the medical institutions.

• Within 30 days of the initial report,the Principal Investigator should also report the investigator or subinvestigator reports the serious adverse event as a follow-up report to the certified review board.

• When received reccomendation other than the study continuation from the certified review board, the Principal Investigator should decide of details of the need and measures,and report it to all Investigators.

• The Principal Investigator should also report the investigator or subinvestigator reports the serious adverse event as a follow-up report to Minister of Health, Labour and Welfare.

ii) Request evaluations to the Data and Safety Monitoring Committee

Same as "18.4.1 Expedited Reporting（After the certified review board approval）(2) Response of the Principal Investigator 1)ii) "

3）For death or life-threatening adverse event for which no causal relationship with a study drug

i) Request evaluations to the Data and Safety Monitoring Committee

Same as "18.4.1 Expedited Reporting（After the certified review board approval）(2) Response of the Principal Investigator 1) ⅱ) "

18.4.2 Ordinary Reporting (excluding Expedited Reporting) (After the certified review board approval)

(1) Reporting by the investigator or subinvestigator

1) If becoming aware of any serious adverse event requiring ordinary reporting excluding expedited reporting, the investigator or subinvestigator should enter necessary information into the serious adverse event form on the FLADS® system and report it to the Principal Investigator and the manager of the medical institution of the investigator or subinvestigator as an initial report within 7 days of becoming aware of the event. However, reporting to the manager of the medical institution should be in accordance with the procedure at each medical institution (the same hereinafter).2) The investigator or subinvestigator should also report the serious adverse event to the Principal Investigator and the manager of the medical institution as a follow-up report via the serious adverse event form on the FLADS® system within 14 days of becoming aware of the event. If requested by the Principal Investigator to report detailed information that is not included in the follow-up report, the investigator or subinvestigator should conduct a necessary and thorough investigation as directed, and report the results of the investigation to the Principal Investigator.

(2) Response of the Principal Investigator

The Principal Investigator should thoroughly investigate the evalution of its causal relationship with the protocol treatment, and respond regarding the following.For expected serious adverse events for which a causal relationship with a study drug(other than death or life-threatening adverse event)

i) Report to the medical institution , the certified review board ,Minister of Health, Labour and Welfare

• The Principal Investigator should also report the investigator or subinvestigator reports the serious adverse event as a follow-up report to the managers of all the medical institutions.

• Within 30 days of the initial report,the Principal Investigator should also report the investigator or subinvestigator reports the serious adverse event as a follow-up report to the certified review board.

• When received reccomendation other than the study continuation from the certified review board, the Principal Investigator should decide of details of the need and measures,and report it to all Investigators.

• The Principal Investigator should also report the investigator or subinvestigator reports the serious adverse event as a follow-up report to Minister of Health, Labour and Welfare.

19 Data Accumulation and Statistical Analysis

19.1 Accumulation and Handling of Data

(1) Data obtained from registered subjects will be accumulated in the JACCRO Study Secretariat through the FLADS® system.

(2) The Principal Investigator will determine how to handle registered subjects and accumulated data after duscussing with the Planning and Promotion Committee as specified in 19.3 "Analysis Sets."

19.2 Statistical Analysis of Data

Data analysis will be performed by the JACCRO statistical analysis section.

Details of statistical analyses and analytical methods are provided in a separate statistical analysis plan. Although this statistical analysis plan may be revised even after the start of data accumulation, the plan will be finalized before data lock following the completion of accumulation and verification of all data.

19.3 Analysis Sets

This study defines the following three analysis sets: full analysis set (FAS), per protocol set (PPS), and safety population (SP). The definition of each analysis set is shown in the table below.

Table 19.3 Analysis Sets

Analysis item	Analysis set	Definition of each analysis set
Efficacy	Full Analysis Set (FAS)	A group consisting of all subjects who have met the major registration criteria, have received at least one dose of the protocol treatment, and have had subsequent evaluable efficacy data
	Per Protocol Set (PPS)	A group consisting of all subjects who have completed the prespecified minimum protocol treatment, have had evaluable primary endpoint data, and have had no major protocol violation
Safety	Safety Population (SP)	A group consisting of all subjects who have received at least one dose of the protocol treatment

19.4 Analysis of the Primary Endpoint

Deepness of Response (DpR)

The t-test with Welch's adjusted degree of freedom will be performed because of expected different distributions between the two groups.

19.5 Secondary Endpoints and the Analysis

(1) Early tumor shrinkage (ETS) at Week 8

The percentage of subjects in each group and a 95% confidence interval for the percentage will be calculated. The difference in and the ratio of the percentages of subjects in the two groups and 95% confidence interval for them will be calculated to evaluate the differences between the groups and compare them with the chi-square test.

(2) Response rate (RR)

The percentage of subjects in each group and a 95% confidence interval for the percentage will be calculated. The difference in and the ratio of the percentages of subjects in the two groups and 95% confidence interval for them will be calculated to evaluate the differences between the groups and compare them with the chi-square test.

(3) Deepness of response (DpR) by Month 4

The t-test with Welch's adjusted degree of freedom will be performed because of expected different distributions between the two groups.

(4) Time to treatment failure (TTF)

A survival function will be estimated by the Kaplan-Meier method. Differences between two groups will be compared using the log rank test.

(5) Time to tumor growth (TTG)

A survival function will be estimated by the Kaplan-Meier method. Differences between two groups will be compared using the log rank test.

(6) Progression-free survival (PFS)

A survival function will be estimated by the Kaplan-Meier method. Differences between two groups will be compared using the log rank test.

(7) Overall survival (OS)

A survival function will be estimated by the Kaplan-Meier method. Differences between two groups will be compared using the log rank test.

(8) Association between tumor shrinkage (ETS, RR, DpR) and prognosis (PFS, OS)

The strength of the correlation will be assessed using the Spearman's correlation coefficient.
A Cox regression model will be used to assess whether DpR is associated with PFS and OS, respectively.

(9) Resection rate

The percentage of subjects in each group and a 95% confidence interval for the percentage will be calculated. The difference in and the ratio of the percentages of subjects in the two groups and 95% confidence interval for them will be calculated to evaluate the differences between the groups and compare them with the chi-square test.

(10) R0 resection rate

The percentage of subjects in each group and a 95% confidence interval for the percentage will be calculated. The difference in and the ratio of the percentages of subjects in the two groups and 95% confidence interval for them will be calculated to evaluate the differences between the groups and compare them with the chi-square test.

(11) Safety (incidence and severity of adverse events)

The frequency of adverse events reported in the safety population will be tabulated by grade, and the incidence of individual adverse events and a 95% confidence interval for the incidence will be calculated.

19.6 Significance Level and Confidence Coefficient

Significance level, α = 5% (two-sided); Confidence coefficient, 95% (two-sided)

19.7 Final Analysis

After completion of the follow-up period, the JACCRO statistical analysis section will finalize the data through the final investigation and then perform analyses of all the endpoints as specified in the statistical analysis plan to prepare a statistical analysis report.

20 Discontinuation or Suspension of the Entire Study

The entire study will be discontinued in either of the following cases. If study discontinuation has been determined, the Principal Investigator will notify the investigator or subinvestigator at each medical institution of study discontinuation. The investigator or subinvestigator at each medical institution will report the study discontinuation at each medical institution as appropriate.

(1) If the Data and Safety Monitoring Committee recommends discontinuation of the study and the Principal Investigator considers it impossible to continue the study

(2) Other cases where it is inevitable to discontinue the entire study

21 Study Periods

Enrollment period: July 2015 to June 2019 (for 4 years)

Follow-up period: For 3 years after registration of the last subject

Study period: July 2015 to June 2022 (for 7 years)

22 Ethical Considerations

22.1 Regulations to be Complied with (After the certified review board approval）

This study will be conducted in compliance with Clinical Trials Act (Act No. 16 of 2017).

22.2 Information Provided for Subjects and Informed Consent

The investigator or subinvestigator will provide each subject with a written information sheet that includes necessary information and explain the contents listed in 22.3 “Items to be Explained” in details orally and in wrting to ensure that the subject has sufficiently understood the necessary information. If any modification has been made to the written information sheet in each medical institution in accordance with the criteria established by the institutional review board (IRB), etc., the revised written information sheet will be sent to the Principal Investigator.

After providing the subject with an opportunity to ask questions and sufficient time to determine whether or not to take part in the study, the subject’s consent to study participation will be obtained. If the subject voluntarily has consented to participation in the study, the investigator who has provided explanations and the subject who has been given the explanations and provided consent will sign their names with the date of consent on the designated consent form or the consent form for this study approved at each medical institution. The investigator or subinvestigator will give a copy of the signed consent form to the subject. The original copy of the signed consent form will be kept at the medical institution.

22.3 Items to be Explained

The written information sheet for this study should include the following information:

(1) Introduction

(2) Your disease and treatment

(3) Standard treatment

(4) Clinical trials

(5) Purpose of this clinical study

(6) Drugs to be used in this study

(7) Number of patients participating in this study and duration of the study

(8) Method for this clinical study

(9) Expected benefits

(10) Anticipated adverse reactions

(11) Cost burden

(12) Treatment and compensation for health damage

(13) Freedom of refusing to provide consent and withdrawing consent to study participation

(14) Treatment in case you do not participate in this study

(15) Cases of discontinuation of this study

(16) Cases where new important information has been obtained

(17) Access to data of this study

(18) Disclosure of information obtained from this study

(19) Methods for storing and disposing of specimens and information

(20) Publication of study results

(21) Secondary use of data

(22) Protection of your human rights

(23) Conflict of interest and intellectual property

(24) Instructions to follow after consenting to the study participation

(25) Freedom of asking question and inquiry services

(26) Implementation system for this study

22.4 Approval by the certified review board (After the certified review board approval）

The study has been approved by the following certified review board.

Kagawa University Hospital Certified Review Board

Certification Numbe：CRB6200005

Location: Kagawa Prefecture

Contact for complaints and inquiries：

Kagawa University Hospital Certified Review Board Secretariat

1750-1 Ikenobe, Miki-cho, Kita-gun, Kagawa Prefecture, 761-0793 Japan

E-mail：kenkyu@med.kagawa-u.ac.jp
Phone：+81-87-891-2011　Fax：+81-87-891-2016

22.5 Protection of Privacy

In the case where the investigator or subinvestigator provides outside of the medical institution with case report forms, adverse event data, or other related data, subject data will be transferred using anonymized subject numbers, etc. The name of each subject will not be disclosed to the study office or the JACCRO Study Secretariat by the medical institution, and all identifications and referrals of registered subjects will be performed using registration numbers issued at the time of registration. In this study, subject registration and reporting will be performed via the Internet using a cryptographic communications protocol called SSL to ensure that the privacy of each subject will be protected when data are handled. In addition, utmost efforts will be made to protect personal information when case report forms are submitted outside each medical institution. Any result of this study made public will not include information that would identify each subject to protect the confidentiality of the identity of each subject. No subject data obtained from this study will be used for purposes other than the purpose of this study.

22.6 Protocol Compliance

Investigators participating in this study will comply with the study protocol as far as the safety and human rights of subjects are not affected.

22.7 Compensation for Health Damage

Participation in this study may lead to the development of adverse events. If any adverse event occurs and results in health damage in a subject, the investigator or subinvestigator and the medical institution will take the best possible measures, including appropriate treatment and other necessary procedures. In such cases, the treatment will be provided by health insurance and the subject will pay some of the medical expenses that are not covered by health insurance. The subject will not use any insurance for compensation or receive any monetary compensation, such as ex gratia payments or various benefits.

23 Deviations from or Modifications of the Protocol

23.1 Modification of the Protocol (After the certified review board approval）

(1) Revision

Revision refers to a partial modification of the protocol that may increase the risk to subjects or is related to the primary endpoint of the study. When a revision is made to the protocol, after consulting the Data and Safety Monitoring Committee and obtaining approval of the ethics review committee of the JACCRO, the Principal Investigator will submit the revised protocol to the certified review board. After the certified review board approval, the Principal Investigator will inform the investigator at each medical institution

(2) Amendment

Amendment refers to a modification of the protocol that may not increase the risk to subjects and is not related to the

primary endpoint of the study (including extension of the registration period). After obtaining approval of the ethics review committee of the JACCRO, the Principal Investigator will submit the amended protocol to the certified review board. After the certified review board approval, the Principal Investigator will inform the investigator at each medical institution..

(3) Memorandum

Memorandum refers to not a modification of the protocol but a supplementary explanation about the protocol that is provided to persons involved in the study to reduce variation in the interpretation of the text or raise awareness.

23.2 Modification of the Written Information for Subjects (After the certified review board approval)

If any revision or amendment of the written information and informed consent form is needed associated with a modification of the protocol, and obtaining approval of the ethics review committee of the JACCRO, the Principal Investigator will submit the revised or amended protocol to the certified review board. After the certified review board approval, the Principal Investigator will inform the investigator at each medical institution.

23.3 Deviations from the Protocol

Any non-compliance with the protocol is defined as a deviation from the protocol.

24 Quality Control and Quality Assurance

24.1 Central Monitoring

To confirm that the study is being conducted safely and in compliance with the protocol and that data are being collected accurately, central monitoring will be performed as specified in a separate procedure based on case report form data accumulated at the data center. If any case in question is found through central monitoring, an inquiry will be made at the relevant medical institution.

Monitoring items

(1) Compliance with the inclusion criteria (eligibility, etc.)

(2) Compliance with the protocol (deviations from the protocol, etc.)

(3) Response to serious adverse events

(4) Occurrence of adverse events

(5) Other safety issues

(6) Omissions and inconsistencies in the entries

Specific monitoring procedures will follow the separate data management plan.

24.2 Monitoring at Individual Medical Institutions(After the certified review board approval)

A representative designated by the JACCRO or the Principal Investigator will perform monitoring at the medical institutions in the study as specified in a separate procedure to confirm that the study is being conducted safely and in compliance with the protocol.

Monitoring at the medical institutions in the study is designed to confirm that the study is being conducted safely and in

compliance with the protocol and that data are being collected accurately by checking the entries into the FLADS® system against source documents. Source documents refer to any original documents, data, and records. The investigator at each medical institution and the manager of the medical institution should ensure that the JACCRO Study Secretariat or its designated representative etc. will have direct access to the source documents.

In monitoring at the medical institutions in the study, approximately 10% of all the registered subjects will be subject to source data verification (SDV) and themedical institutions will be selected randomly.

24.3 Auditing

A representative designated by the JACCRO or the Principal Investigator will perform auditing at participating medical institutions when necessary as specified in a separate procedure. In such cases, the designated person in charge of auditing will contact a participating medical institution in advance to arrange a visit for auditing. The person in charge of auditing may request a visit to any other participating medical institutions used during the study period. The investigator at each participating medical institution and the head of the medical institution should ensure that the person in charge of auditing will have direct access to all the study-related source documents.

25 Retention of Specimens and Records

25.1 Retention of Specimens

Collected specimens will be disposed of appropriately as per the procedure at each medical institution after completion of the analyses as specified in the protocol.

25.2 Retention of Records(After the certified review board approval）

The JACCRO Study Secretariat will retain study-related documents for 5 years after since the completion or discontinuation of this study. However, if the Principal Investigator determines that secondary use of the data is useful within the retention period, the retention period will be extended.When the retention period is extended, notification and publication of the extension will be made on the JACCRO website.

26 Periodic Report(After the certified review board approval）

26.1 Response of the Principal Investigator(After the certified review board approval）

(1) The Principal Investigator shall report to the certified review board the matters stipulated in Article 59, paragraph 1 of the Enforcement Regulations of the Clinical Research Act every year from the date of submission of the implementation plan to the Minister of Health, Labour and Welfare, within two months after the expiration of said period.

(2) The Principal Investigator should report to the Minister of Health, Labour and Welfare on the status of this study within one month of the date on which the certified review board expressed its opinion on the name of the certified review board stated in the protocol, whether or not this study is to be continued of by said the certified review board, and matters stipulated in Article 59, paragraph 1 of the Enforcement Regulations of the Clinical Research Act.

(3) (3) The Principal Investigator should immediately provide the information to the investigator at each medical

institution after making the report described in (1) above.

26.2 Response of the investigator(After the certified review board approval）

The investigators should report the periodic reports provided by the Principal Investigator to the managers of the medical institutions.

27 Conflict of Interest and Cost Burden of the Study

27.1 Source of Funds and Financial Relationship

The present study is an investigator-initiated study conducted by using a research fund for the study sponsored by Merck Serono Co., Ltd. based on the “Investigator-Sponsored Trial (IST) Contract” concluded between Merck Serono Co., Ltd. and the Nonprofit Organization Japan Clinical Cancer Research Organization (JACCRO). Any decision on the planning, conduct, and publication of the study will be made by the research organization for this study. The research will be conducted while the transparency and reliability of the research are being assured so that the opinions of Merck Serono Co., Ltd. will not influence the results of the research. Merck Serono Co., Ltd. will not demand any commercially advantageous position in exchange for the sponsorship.

27.2 Conflict of Interest(After the certified review board approval）

27.2.1 Conflict of Interest Management System

Conflicts of interest shall be managed in accordance with the "Standard Operating Procedures for the Management of Conflicts of Interest under the Clinical Research Act, Ver. 1.0.”

27.2.2 Conflict of Interest Matters for the Principal Investigator, **the investigators**, and **the subinvestigators**

See Appendix

27.3 Costs Related to Study Treatment

All the medical costs related to this study will be covered within the standard health insurance, and the amount of self-payment for medication, observation, tests, radiation, and other drugs used during the clinical study period will be paid by individual subjects.

28 Publication and Attribution of Study Results

Results achieved from this study shall belong to the JACCRO. Merck Serono Co., Ltd. has the right to exclusively use the results of the study. Results obtained from this study will be made public irrespective of the results.

Publication of the study results will be a joint publication with the study participating institutions in joint names of the principal investigator, statistical analysis manager (contributor), planning and promotion committee members, researchers of the participating institutions where patients were registered. The principal investigator will select the authors through discussion with planning and promotion committee members and statistical analysis manager. The lead author will be selected taking into consideration of the physician (investigator or subinvestigator) and the participating institution with the largest number of patients registered. Selection of authors for conference presentation will follow the order mentioned above.

29 Registration of Study Protocol(After the certified review board approval）

Information on this study is published in the database "jRCT: Japan Registry of Clinical Trials (https://jrct.niph.go.jp/)" maintained by the Ministry of Health, Labour and Welfare of Japan.

jRCT number: jRCTs061180022

30 Study Implementation System

[redacted]

30.1.1 Role and Responsibility of the Principal Investigator

The Principal Investigator will be responsible for the preparation of a protocol, the registration of a synopsis of the study, the publication of research results, the supervision and management of persons involved in the study, and the control and supervision of the progress of the study.

30.2 Planning and Promotion Committee Members

[redacted]

30.2.1 Role and Responsibility of the Planning and Promotion Committee

The Planning and Promotion Committee will cooperate with and assist the Principal Investigator in the preparation of a protocol, the registration of a synopsis of the study, the publication of research results, the supervision and management of persons involved in the study, and the control and supervision of the progress of the study.

30.3 External Response Review Committee Members

[redacted]

30.3.1 Role and Responsibility of the External Response Review Committee

The External Response Review Committee will perform external response reviews to determine deepness of response, early tumor shrinkage, and response rate.

30.4 Statistical Analysis Manager

[redacted]

30.4.1 Role and Responsibility of the Statistical Analysis Manager

The Statistical Analysis Manager will be responsible for the preparation of a statistical analysis plan and a statistical analysis report.

30.5 Data and Safety Monitoring Committee Members

Chairperson:

Kenji Omura — Department of Surgery/Medical Oncology, Ageo Central General Hospital

Committee members:

Michio Itabashi — Department of Surgery II, Tokyo Women's Medical University

Kouhei Akazawa — Department of Medical Informatics, Niigata University Medical and Dental Hospital

30.5.1 Role and Responsibility of the Data and Safety Monitoring Committee

(1) The Data and Safety Monitoring Committee will periodically assess the progress of the clinical study, safety data, and clinical efficacy endpoints and suggest the continuation, modification, and discontinuation of the clinical study to the Principal Investigator.

(2) The Data and Safety Monitoring Committee will assess the results of efficacy analyses, and the results of safety analyses as reference information, to prepare a written recommendation on the continuation or discontinuation of the clinical study or the modification of the clinical study protocol, and submit it to the Principal Investigator. Based on this recommendation, the Principal Investigator will determine the continuation, modification, or discontinuation of the clinical study.

30.6 Participating Institutions and Investigators

See Attachment.

30.7 JACCRO Study Secretariat

Nonprofit Organization Japan Clinical Cancer Research Organization (JACCRO)

6F Jinbocho Kyowa Building, 1-64-3 Kandajinbocho,, Chiyoda -ku, Tokyo 101-0051, Japan

Phone: +81-3-6811-0433 Fax: +81-3-6811-0434

E-mail: cc13.dc@jaccro.or.jp

30.8 Contact Information

[redacted]

JACCRO Study Secretariat

Phone: +81-3-5579-9882 E-mail: cc13.dc@jaccro.or.jp

30.9 JACCRO CC-13 Data Center

AC Medical Co., Ltd.

4F Harumi Center Building, 2-5-24 Harumi, Chuo-ku, Tokyo 104-0053, Japan

Phone: +81-3-6811-0433 Fax: +81-3-3520-9815

E-mail: jaccro_cc-13_datacenter@md-stat.co.jp

31 References

(1) Van Cutsem E, Kohne CH, Hitre E, Zaluski J, Chien CC, Makhson A, et al. Cetuximab and chemotherapy as initial treatment for metastatic colorectal cancer. N Engl J Med 2009; 360: 1408-17.

(2) Bokemeyer C, Bondarenko I, Hartmann JT, De Braud F, Schuch G, Zubel A, et al. Efficacy according to biomarker status of cetuximab plus FOLFOX-4 as first-line treatment for metastatic colorectal cancer: the OPUS study. Ann Oncol 2011; 22: 1535-46.

(3) Yamaguchi T, Tsuji A, Sunakawa Y, Nakamura M, Kochi M, Denda T, et al. A phase II study of cetuximab and mFOLFOX6 in metastatic colorectal cancer. J Clin Oncol 32, 2014 (suppl 3; abstr 611)

(4) Tsuji A, Sunakawa Y, Denda T, Takinishi Y, Kotaka M, Tanioka H, et al. A phase II study of cetuximab in combination with S-1 and oxaliplatin in first-line treatment for metastatic colorectal cancer. J Clin Oncol 32, 2014 (suppl 3; abstr 571)

(5) NCCN Clinical Practice Guidelines in Oncology (NCCN Guidelines®) version 1.2015 [Internet]. U.S.: National Comprehensive Cancer Network; 2014 [updated 2014 August 20]. Availablefrom: http://www.nccn.org/professionals/physician_gls/pdf/colon.pdf

(6) Loupakis F, Cremolini C, Masi G, Lonardi S, Zagonel V, Salvatore L, et al. Initial therapy with FOLFOXIRI and bevacizumab for metastatic colorectal cancer. N Engl J Med 2014; 371: 1609-18.

(7) Japanese Society for Cancer of the Colon and Rectum. JSCCR Guidelines 2014 for the Treatment of Colorectal Cancer. Int J Clin Oncol 2015; 20; 207-39.

(8) Falcone A, Masi G, Allegrini G, Danesi R, Pfanner E, Brunetti IM, et al. Biweekly chemotherapy with oxaliplatin, irinotecan, infusional Fluorouracil, and leucovorin: a pilot study in patients with metastatic colorectal cancer. J Clin Oncol. 2002; 20: 4006-14.

(9) Masi G, Allegrini G, Cupini S, Marcucci L, Cerri E, Brunetti I, et al. First-line treatment of metastatic colorectal cancer with irinotecan, oxaliplatin and 5-fluorouracil/leucovorin (FOLFOXIRI): results of a phase II study with a simplified biweekly schedule. Ann Oncol. 2004; 15: 1766-72.

(10) Falcone A, Ricci S, Brunetti I, Pfanner E, Allegrini G, Barbara C, et al. Phase III trial of infusional fluorouracil, leucovorin, oxaliplatin, and irinotecan (FOLFOXIRI) compared with infusional fluorouracil, leucovorin, and irinotecan (FOLFIRI) as first-line treatment for metastatic colorectal cancer: the Gruppo Oncologico Nord Ovest. J Clin Oncol. 2007; 25:1670-6.

(11) Sunakawa Y, Fujita K, Ichikawa W, Ishida H, Yamashita K, Araki K. et al. A phase I study of infusional 5-fluorouracil, leucovorin, oxaliplatin and irinotecan in Japanese patients with advanced colorectal cancer who harbor UGT1A1*1/*1,*1/*6 or *1/*28. Oncology. 2012; 82:242-8.

(12) Lenz H, Niedzwiecki D, Innocenti F, Blanke C, Mahony M.R, O'Neil B.H, et al. CALGB/SWOG 80405: Phase III trial of FOLFIRI or mFOLFOX6 with bevacizumab or cetuximab for patients with expanded RAS analyses in untreated metastatic adenocarcinoma of the colon or rectum (mCRC). Ann Oncol 2014; 25(Supplement 5) : v1–v41.

(13) Stintzing S, Modest D.P, Fischer von Weikersthal L, Decker T, Kiani A, Vehling-Kaiser U, et al. Independent radiological evaluation of objective response rate, early tumour shrinkage, and depth of response in the FIRE-3

study: Analysis in the final RAS evaluable population. Ann Oncol 2014; 25(Supplement 5): v1–v41.

(14) Salvatore L, Cremolini C, Loupakis F, Masi G, Schirripa M, Marmorino F, et al. FOLFOXIRI plus bevacizumab (BV) or plus anti-EGFR antibodies in RAS and BRAF wild-type (wt) metastatic colorectal cancer (mCRC) patients (pts): analysis of tumor response. Ann Oncol 2014; 25(Supplement 4): iv167–iv209.

(15) Cremolini C, Loupakis F, Antoniotti C, Lonardi S, Masi G, Salvatore L, et al. Early Tumor Shrinkage and Depth of Response predict long-term outcome in metastatic colorectal cancer patients treated with first-line chemotherapy plus bevacizumab: results from phase III TRIBE trial by the Gruppo Oncologico del Nord Ovest. Ann Oncol 2015; 26: 1188-94.

(16) Mansmann U, Sartorius U, Laubender R, Giessen C, Esser R, Heinemann V, et al. Quantitative analysis of the impact of deepness of response on post-progression survival time following first-line treatment in patients with mCRC. Ann Oncol 2013; 24(Supplement 4): iv11–iv24.

(17) Venook AP, Tabernero J. Progression-free survival: helpful biomarker or clinically meaningless end point?. J Clin Oncol 2015; 33: 4-6.

(18) Rivera F, Karthaus M, Hecht JR, Fasola G, Canon JL, Koukakis R, et al. First-line treatment with modified FOLFOX6 (mFOLFOX6) + panitumumab (pmab) or bevacizumab (bev) in wild-type (WT) RAS metastatic colorectal carcinoma (mCRC): Tumor response outcomes beyond RECIST. J Clin Oncol 2015; 33(suppl 3; abstr 660).

(19) Nakajima T, Yamaguchi T, Fujii T, Shimada Y, Fukui I, Sone S, et al. Efforts of the JACCRO in the Management of Clinical Trials to Develop Anticancer Agents - Support using the Flexible License Assisted Data Server (FLADS) - Surgical Therapy

(20) Masi G, Loupakis F, Salvatore L, Fornaro L, Cremolini C, Cupini S, et al. Bevacizumab with FOLFOXIRI (irinotecan, oxaliplatin, fluorouracil, and folinate) as first-line treatment for metastatic colorectal cancer: a phase 2 trial. Lancet Oncol. 2010; 11:845-52.

(21) Cremolini C, Loupakis F, Salvatore L, Lonardi S, Battaglin F, Gumucci T, et al. Modified FOLFOXIRI plus cetuximab (cet) as induction treatment in unresectable metastatic colorectal cancer (mCRC) patients (pts): Preliminary results of the phase II randomized Macbeth trial by GONO group. J Clin Oncol 2014; 32(suppl; abstr 3596).

(22) Grothey A, Fakih M and Tabernero J, Management of BRAF-mutant metastatic colorectal cancer: a review of treatment options and evidence-based guidelines. Ann Oncol 2021; 32: 959-67.
