# Supplementary material for: Modified FOLFOXIRI plus cetuximab versus bevacizumab in RAS wild-type metastatic colorectal cancer: a randomized phase II DEEPER trial
Source: Nat Commun. 2024 Nov 25;15:10217. doi: 10.1038/s41467-024-54460-2 (PMC11589592; doi:10.1038/s41467-024-54460-2)
Supplement: Supplementary file 2 — Reporting Summary [file 41467_2024_54460_MOESM2_ESM.pdf]

nature portfolio

Corresponding author(s): Yu Sunakawa

Last updated by author(s): Sep 28, 2024

Reporting Summary

Nature Portfolio wishes to improve the reproducibility of the work that we publish. This form provides structure for consistency and transparency in reporting. For further information on Nature Portfolio policies, see our Editorial Policies and the Editorial Policy Checklist.

Statistics

For all statistical analyses, confirm that the following items are present in the figure legend, table legend, main text, or Methods section.

n/a	Confirmed	
[ ]	[x]	The exact sample size (*n*) for each experimental group/condition, given as a discrete number and unit of measurement
[x]	[ ]	A statement on whether measurements were taken from distinct samples or whether the same sample was measured repeatedly
[ ]	[x]	The statistical test(s) used AND whether they are one- or two-sided<br>*Only common tests should be described solely by name; describe more complex techniques in the Methods section.*
[ ]	[x]	A description of all covariates tested
[ ]	[x]	A description of any assumptions or corrections, such as tests of normality and adjustment for multiple comparisons
[ ]	[x]	A full description of the statistical parameters including central tendency (e.g. means) or other basic estimates (e.g. regression coefficient) AND variation (e.g. standard deviation) or associated estimates of uncertainty (e.g. confidence intervals)
[ ]	[x]	For null hypothesis testing, the test statistic (e.g. *F*, *t*, *r*) with confidence intervals, effect sizes, degrees of freedom and *P* value noted<br>*Give P values as exact values whenever suitable.*
[x]	[ ]	For Bayesian analysis, information on the choice of priors and Markov chain Monte Carlo settings
[x]	[ ]	For hierarchical and complex designs, identification of the appropriate level for tests and full reporting of outcomes
[ ]	[x]	Estimates of effect sizes (e.g. Cohen's *d*, Pearson's *r*), indicating how they were calculated

Our web collection on statistics for biologists contains articles on many of the points above.

Software and code

Policy information about availability of computer code

Data collection	Data were collected using Free license assisted data server (FLADS) system (Japan Clinical Cancer Research Organization, Tokyo, Japan)
Data analysis	All analyses were conducted using SAS version 9.4 (SAS Institute, Cary, NC, USA) .

For manuscripts utilizing custom algorithms or software that are central to the research but not yet described in published literature, software must be made available to editors and reviewers. We strongly encourage code deposition in a community repository (e.g. GitHub). See the Nature Portfolio guidelines for submitting code & software for further information.

Data

Policy information about availability of data

All manuscripts must include a data availability statement. This statement should provide the following information, where applicable:

- Accession codes, unique identifiers, or web links for publicly available datasets
- A description of any restrictions on data availability
- For clinical datasets or third party data, please ensure that the statement adheres to our policy

The authors declare that all relevant data used to conduct the analyses are available within the article. To protect the privacy and confidentiality of patients in this study, clinical data are not made publicly available in a repository or the supplementary material of the article but can be requested at any time from the corresponding author. Any requests will be reviewed within a time frame of 2 to 3 weeks by the JACCRO to verify whether the request is subject to any intellectual property or confidentiality obligations. The data will be made available for one month. All data shared will be de-identified.

Research involving human participants, their data, or biological material

Policy information about studies with human participants or human data. See also policy information about sex, gender (identity/presentation), and sexual orientation and race, ethnicity and racism.

Reporting on sex and gender	This study enrolled male and female patients. We reported the rate of male/female in the Table 1. We did not analyze clinical data according to sex.
Reporting on race, ethnicity, or other socially relevant groupings	This study enrolled only Japanese patients and were performed in Japan; therefore, we did not describe the information about race.
Population characteristics	Population characteristics are detailed in Table 1. Eligible participants were adults aged ≥20 years at the time of enrolment with a histologically confirmed diagnosis of RAS wild-type (KRAS exons 2, 3, and 4, and NRAS exons 2, 3, and 4), unresectable mCRC, and with a measurable lesion according to the Response Evaluation Criteria in Solid Tumours (RECIST) 1.1 and ECOG PS of 0 or 1 (patients aged ≥71 years were required to have a PS of 0).
Recruitment	Patients were recruited by investigators based on prespecified inclusion/exclusion criteria.
Ethics oversight	Study protocol was approved by the JACCRO (study center) and institutional review board at each institution, and all patients provided written informed consent.

Note that full information on the approval of the study protocol must also be provided in the manuscript.

Field-specific reporting

Please select the one below that is the best fit for your research. If you are not sure, read the appropriate sections before making your selection.

[x] Life sciences [ ] Behavioural & social sciences [ ] Ecological, evolutionary & environmental sciences

For a reference copy of the document with all sections, see nature.com/documents/nr-reporting-summary-flat.pdf

Life sciences study design

All studies must disclose on these points even when the disclosure is negative.

Sample size	A sample size of 180 patients per group (360 patients in total) was planned, taking into account a 5% dropout rate, including withdrawals prior to administration. This was calculated to ensure 85% power to detect DpR superiority for m-FOLFOXIRI + cetuximab versus m-FOLFOXIRI + bevacizumab with a two-sided significance level of 0·05, assuming a difference in median DpR of 12·5% (standard deviation 42% and 34% for cetuximab and bevacizumab, respectively), based on findings from previous studies of anti-EGFR mAbs.
Data exclusions	No data was excluded from the analysis.
Replication	This study was a clinical study with human participants. Here, replication of data set is not applicable for our study.
Randomization	Patients were randomized 1:1 to the standard treatment group or the study treatment group. A randomized phase 2 study.
Blinding	Blinding was not applicable because no unapproved drugs were used in this study; all drugs are available in clinical practice. Furthermore, we cannot be blinded because of the different methods of administration in the chemotherapy regimen compared.

Reporting for specific materials, systems and methods

We require information from authors about some types of materials, experimental systems and methods used in many studies. Here, indicate whether each material, system or method listed is relevant to your study. If you are not sure if a list item applies to your research, read the appropriate section before selecting a response.

Materials & experimental systems

n/a	Involved in the study	
x		Antibodies
x		Eukaryotic cell lines
x		Palaeontology and archaeology
x		Animals and other organisms
	x	Clinical data
x		Dual use research of concern
x		Plants

Methods

n/a	Involved in the study	
x		ChIP-seq
x		Flow cytometry
x		MRI-based neuroimaging

Clinical data

Policy information about clinical studies

All manuscripts should comply with the ICMJE guidelines for publication of clinical research and a completed CONSORT checklist must be included with all submissions.

Clinical trial registration	UMIN000018217, jRCTs061180022
Study protocol	The protocol has been submitted along with the manuscript.
Data collection	Data were entered into the EDC by the investigators and clinical research coordinators at each site. Participants were enrolled in the DEEPER trial between xx xx, 20xx and xx xx, 20xx.
Outcomes	The primary endpoint of the study was depth of response (DpR), defined as the sum of the longest diameters of RECIST target lesions at the nadir in the absence of progression, subtracted from the sum of the longest diameters of target lesions at baseline and then divided by the sum of the longest diameters of target lesions at baseline in the per protocol set (PPS) consisting of patients evaluable for DpR, which was evaluated every 8 weeks until disease progression. Responses were evaluated according to the RECIST version 1.1 by the investigators and validated by an external review board. Secondary endpoints included ORR, early tumor shrinkage (ETS) at week 8, progression-free survival (PFS), overall survival (OS), resection rate, and safety (incidence and severity of AEs).

Plants

Seed stocks	*Report on the source of all seed stocks or other plant material used. If applicable, state the seed stock centre and catalogue number. If plant specimens were collected from the field, describe the collection location, date and sampling procedures.*
Novel plant genotypes	*Describe the methods by which all novel plant genotypes were produced. This includes those generated by transgenic approaches, gene editing, chemical/radiation-based mutagenesis and hybridization. For transgenic lines, describe the transformation method, the number of independent lines analyzed and the generation upon which experiments were performed. For gene-edited lines, describe the editor used, the endogenous sequence targeted for editing, the targeting guide RNA sequence (if applicable) and how the editor was applied.*
Authentication	*Describe any authentication procedures for each seed stock used or novel genotype generated. Describe any experiments used to assess the effect of a mutation and, where applicable, how potential secondary effects (e.g. second site T-DNA insertions, mosiacism, off-target gene editing) were examined.*
