# Supplementary material for: Modified FOLFOXIRI plus cetuximab versus bevacizumab in RAS wild-type metastatic colorectal cancer: a randomized phase II DEEPER trial
Source: Nat Commun. 2024 Nov 25;15:10217. doi: 10.1038/s41467-024-54460-2 (PMC11589592; doi:10.1038/s41467-024-54460-2)
Supplement: Supplementary file 3 — Peer Review File [file 41467_2024_54460_MOESM3_ESM.pdf]

nature portfolio

Peer Review File

Modified FOLFOXIRI plus cetuximab versus bevacizumab in RAS wild-type metastatic colorectal cancer: a randomized phase II DEEPER trial

Open Access This file is licensed under a Creative Commons Attribution 4.0 International License, which permits use, sharing, adaptation, distribution and reproduction in any medium or format, as long as you give appropriate credit to the original author(s) and the source, provide a link to the Creative Commons license, and indicate if changes were made. In the cases where the authors are anonymous, such as is the case for the reports of anonymous peer reviewers, author attribution should be to 'Anonymous Referee' followed by a clear attribution to the source work. The images or other third party material in this file are included in the article's Creative Commons license, unless indicated otherwise in a credit line to the material. If material is not included in the article's Creative Commons license and your intended use is not permitted by statutory regulation or exceeds the permitted use, you will need to obtain permission directly from the copyright holder. To view a copy of this license, visit http://creativecommons.org/licenses/by/4.0/.

Editorial Note: This manuscript has been previously reviewed at another journal that is not operating a transparent peer review scheme. This document only contains reviewer comments and rebuttal letters for versions considered at *Nature Communications*.

REVIEWER COMMENTS

Reviewer #1
Shiozawa et al submit the original research report of the phase 2 randomized trial comparing FOLFOXIRI + cetuximab versus FOLFOXIRI + bevacizumab in the first line treatment of metastatic RAS wildtype colorectal cancer. The authors enrolled 359 patients and demonstrate superiority for the primary endpoint which was depth of response (57.3% vs 46.0%), although there was no difference in PFS or OS between therapies. In a pre-planned sub-group analysis, left sided tumors did show benefit for both PFS and OS. The selected primary end point is odd given the long term follow up before this report in which PFS could have been used, or at least ORR, however the trial provides information in a large population about an important question. I think the authors for the opportunity to review the results of this interesting clinical trial.
Major issues:
Inclusion criteria
1) BRAF: The authors include in their background information the importance of RAS and BRAF testing in selecting a first line regimen, however the trial did not include BRAF V600E sequencing as a requirement and among those patients with known BRAF V600E mutations, they were allowed enrolment. They do describe that ongoing biomarker studies are pending and that the study was started in 2015, however they also pre-planned a sidedness analysis which had similar levels of evidence in 2015 to BRAF being refractory to anti-EGFR antibodies. If any sequencing results from the biomarker analysis are done for BRAF, I think these should be included in the current study. I think this is particularly important when submitting this manuscript to such a high impact journal.
2) Please describe whether liver metastases were assessed for resectability by a multidisciplinary team before enrolment.
Methods:
1) Why is the primary analysis being performed in the per protocol population instead of the intention to treat population? They provide an explanation in the limitations which is not appropriate. It should still be analyzed even with missing data (and those cases are usually considered a worst case scenario if only one CT is available). If they only excluded patients who lacked access to imaging, I think this is actually an intention to treat analysis with patients who have missing data excluded. Patients who did not start therapy are not clearly described about inclusion in the current narrative.
2) There are a large number of subgroup analyses performed, particularly where they start looking at RAS/BRAF wildtype and left sided cancers. It does not appear that there

has been correction for multiple comparisons. Given this, it is important to highlight the large number of comparisons as a weakness in the discussion and that a correction for multiple comparisons was not performed.
3) Why was depth of response chosen instead of PFS or ORR as a surrogate for OS? They outline the utility of depth of response as a surrogate but there is a much larger body of evidence for PFS as an endpoint.
4) Why is age over 20 the eligibility criteria age?
Minor issues:
1) The introduction would benefit from revision or copy editing as the sentence structure and flow of background information is currently poor.
2) Please frame the first sentence of the discussion relative to the primary endpoint. It is reasonably to include the subgroup analysis that is currently outlined in that same paragraph but it is currently misleading and looks like this is a positive trial when it is not.

Reviewer #2
The authors report the outcome data of the ranomized phase-2 DEEPER trial investgiating firs-line treatment with either FOLFOXIRI plus cetuximab or FOLFOXIRI plus bevacizumab. The trial met its primary endpoint and was able to show a significant better tumorresponse to FOLFOXIRI plus cetuximab when compared to FOLFOXIRI plus bevacizumab. Within the population of left sided primaries, this also translated into a significant longer PFS and OS.
This is the first prospective investigation of the both regimen. Therefore it has high priority for clinicians.
The data and methodology is apporpriate. I have read and reviewed the articel before and it has significantly improved since my last review.
The conclusions are well balanced, limitations are clearly stated. At this point no further experiments or data are necessary.

From the view of the clinician, the study is of high interest as the combination of FOLFOXIRI plus Bevacizumab vs. FOLFOXIRI plus Cetuximab has not been compared in the subgroup of RAS wild-type mCRC patents so far. The data is sound and therefore had been selected as oral presentation at ASCO annual meeting two years ago. For medically fit patients with either symptoms or borderline resectable metastatic disease, the DEEPER study provide guidance on how these patient should be treated within the first-line setting. Also the unprecedented median survival time for mCRC will spark further scientific interest to further explore FOLFOXIRI plus cetuximab in first-line mCRC.

The safety profile is as expected and in the range of the FIRE-4.5 Study (using FOLFOXIRI plus Cetuximab in BRAFV600E mutant mCRC) and VOLFI (FOLFOXIRI plus Panitumumab) studies.

Since the oral presentation of the JACRO group, we are urgently awaiting the publication.

Reviewer #3
My expertise are statistical and hence I mainly provide comments on statistical aspects of the manuscript. The manuscript lacks detail to determine whether the statistical approach was appropriate, but it mostly looks appropriate) My comments are:

- Adjustment of multiple comparisons. There seems to be a well defined single primary hypothesis test, but for the secondary analysis no adjustment for multiple comparisons were made. This needs to be mentioned in the analysis section.

- The statement "Among patients with left-sided, RAS/BRAF wild-type tumors...." in the abstract presents an analysis that is not prespecified in the protocol and hence has to be considered very exploratory especially given the number of hypothesis tests that are conducted throughout the manuscript without multiplicity adjustments. Hence, I suggest softening the conclusions.

The protocol states "Details of statistical analyses and analytical methods are provided in a separate statistical analysis plan." I don't see that plan being submitted and the protocol has relatively limited information on how things were done. For example, there is no discussion on how missing data was handled.

"Although this statistical analysis plan may be revised even after the start of data accumulation, the plan will be finalized before data lock following the completion of accumulation and verification of all data." How was the plan changed and did investigators with access to data make the changes? Also, I could not find any way of seeing how the protocol evolved from trial activation to its closure.

Why was missing endpoint data not treated as a missing data problem rather than doing a complete case analysis?

It would be useful to see histograms of the DpR values for both treatment groups. That would help with evaluating the impact of outliers and be informative about the

distribution of the DpR values.

- Failure to justify why DpR is a surrogate for overall survival diminishes the significance.

Response to Reviewers

Reviewer #1:

Major issues:

Inclusion criteria

1) BRAF: The authors include in their background information the importance of RAS and BRAF testing in selecting a first line regimen, however the trial did not include BRAF V600E sequencing as a requirement and among those patients with known BRAF V600E mutations, they were allowed enrolment. They do describe that ongoing biomarker studies are pending and that the study was started in 2015, however they also pre-planned a sidedness analysis which had similar levels of evidence in 2015 to BRAF being refractory to anti-EGFR antibodies. If any sequencing results from the biomarker analysis are done for BRAF, I think these should be included in the current study. I think this is particularly important when submitting this manuscript to such a high impact journal.

Thank you so much for your important remarks. In 2015, when we started this study, *BRAF* testing was not approved in Japan and we could not recognize *BRAF* mutated mCRC. However, we used the primary site as a stratification factor in this study because the evidence on the correlation between the primary site and the efficacy of anti-EGFR antibody drugs was well established. This situation is described and explained in the discussion. The biomarker study is still under analysis and we hope to present it in future conferences and papers, along with OS updates.

2) Please describe whether liver metastases were assessed for resectability by a multidisciplinary team before enrolment.

Our study did not stipulate that resectability of liver metastases be considered prior to patient enrollment. No multidisciplinary team was involved in the study. The study did not prospectively evaluate for liver metastases because the study was not designed to evaluate liver metastasis resection. Therefore, we believe that the prognostic analysis based on R0 resection rate and resection of liver metastases is only exploratory.

Methods:

1) Why is the primary analysis being performed in the per protocol population instead of the intention to treat population? They provide an explanation in the limitations which is not appropriate. It should still be analyzed even with missing data (and those cases are usually considered a worst case scenario if only one CT is available). If they only excluded patients who lacked access to imaging, I

think this is actually an intention to treat analysis with patients who have missing data excluded. Patients who did not start therapy are not clearly described about inclusion in the current narrative.

As we described in Figure 1, the PPS for this study analysis excluded patients who had not received treatment (N = 8), patients with no target lesions (N = 3), and patients for whom the primary endpoint could not be assessed, such as patients who had not undergone imaging evaluation after treatment (N = 19). In addition, the PPS excluded patients in which new lesions appeared at the initial evaluation and patients in which only non-target lesions were progressive (N=8). Actually, previous studies have excluded such patients from the analysis of DpR in CRC trials [*Eur J Cancer* 2015 Sep;51(14):1927-36, *Ann Oncol* 2015 Jun;26(6):1188-1194, *Eur J Cancer* 2021 Feb:144:31-40], and we defined the method of evaluation and analysis of DpR in the same way for the present study.

In our DEEPER trial, although the primary endpoint of DpR was met in *RAS* wild-type patients, there was no difference between the two groups in the secondary endpoint of OS, and some patients were excluded from the analysis, so we believe that we should be cautious about using DpR as the primary endpoint in future clinical trials for mCRC.

We have revised the discussion to explain these points in more detail in the limitation. It is undeniable that the survival data in this trial are exploratory. Therefore, we have modified the discussion to de-emphasize the OS results in *RAS*/*BRAF* wild-type and left-sided patients with respect to the conclusion of the paper.

2) There are a large number of subgroup analyses performed, particularly where they start looking at RAS/BRAF wildtype and left sided cancers. It does not appear that there has been correction for multiple comparisons. Given this, it is important to highlight the large number of comparisons as a weakness in the discussion and that a correction for multiple comparisons was not performed.

Thank you for your valuable comments. In this study, the primary tumor site was incorporated as a stratification factor in *RAS* wild-type patients, while the clinical outcomes were analyzed multiple times for *RAS*/*BRAF* wild-type patients. Although the study was conducted in *RAS* wild-type patients with primary tumor site as a stratification factor, the data for *RAS*/*BRAF* wild-type patients is not conclusive because it was a post hoc analysis and PFS and OS were analyzed without adjusting for multiplicity. However, we believe that the PFS and OS results are clinically meaningful. We have added a note to the discussion regarding this point.

3) Why was depth of response chosen instead of PFS or ORR as a surrogate for OS? They outline the utility of depth of response as a surrogate but there is a much larger body of evidence for PFS as

an endpoint.

Previous CRC trials comparing anti-EGFR antibody drugs with bevacizumab have found significant differences in OS, but almost all trials have found no difference in PFS. When comparing anti-EGFR antibody drugs with bevacizumab for *RAS* wild-type mCRC, PFS may not be appropriate as an endpoint. If OS is used as an endpoint, the prognosis of mCRC is so long that it takes much time to get results and the effect of a new treatment cannot be evaluated quickly. Therefore, we set the DpR, which has been shown to correlate with OS in clinical trials of mCRC, as the endpoint for this study.

4) Why is age over 20 the eligibility criteria age?

In Japan, patients 20 years of age or older were defined as adults and those under 20 years of age as minors. Since patients under 20 years of age are required to obtain consent from a proxy, etc., to participate in clinical trials, we decided to incorporate patients 20 years of age or older in this study.

This is a common eligibility criterion for clinical trials and investigations conducted in Japan.

Minor issues:

1) The introduction would benefit from revision or copy editing as the sentence structure and flow of background information is currently poor.

Thank you for your valuable suggestions. We had included the necessary information within the word limit, but have corrected it as per the reviewer's suggestion.

2) Please frame the first sentence of the discussion relative to the primary endpoint. It is reasonably to include the subgroup analysis that is currently outlined in that same paragraph but it is currently misleading and looks like this is a positive trial when it is not.

We have changed the overall tone of the paper in accordance with the reviewer's remarks. Our study met its primary endpoint, but failed to demonstrate a benefit in the cetuximab arm for the secondary endpoint of survival. Sub-group analysis allowed us to explore the population in which m-FOLFOXIRI plus cetuximab treatment was effective.

Reviewer #3:

- Adjustment of multiple comparisons. There seems to be a well defined single primary hypothesis test, but for the secondary analysis no adjustment for multiple comparisons were made. This needs

to be mentioned in the analysis section.

Thank you for your valuable comments. In this study, the primary tumor site was incorporated as a stratification factor in *RAS* wild-type patients, while the clinical outcomes were analyzed multiple times for *RAS*/*BRAF* wild-type patients. Although the study was conducted in *RAS* wild-type patients with primary tumor site as a stratification factor, the data for *RAS*/*BRAF* wild-type patients is not conclusive because it was a post hoc analysis and PFS and OS were analyzed without adjusting for multiplicity. However, we believe that the PFS and OS results are clinically meaningful. We have added a note to the discussion regarding this point.

- The statement "Among patients with left-sided, RAS/BRAF wild-type tumors...." in the abstract presents an analysis that is not prespecified in the protocol and hence has to be considered very exploratory especially given the number of hypothesis tests that are conducted throughout the manuscript without multiplicity adjustments. Hence, I suggest softening the conclusions.

We have changed the overall tone of the paper in accordance with the reviewer's remarks. Our study met its primary endpoint, but failed to demonstrate a benefit in the cetuximab arm for the secondary endpoint of survival. Sub-group analysis allowed us to explore the population in which m-FOLFOXIRI plus cetuximab treatment was effective.

The protocol states "Details of statistical analyses and analytical methods are provided in a separate statistical analysis plan." I don't see that plan being submitted and the protocol has relatively limited information on how things were done. For example, there is no discussion on how missing data was handled.

We have attached a plan of statistical analysis for this study.

"Although this statistical analysis plan may be revised even after the start of data accumulation, the plan will be finalized before data lock following the completion of accumulation and verification of all data." How was the plan changed and did investigators with access to data make the changes? Also, I could not find any way of seeing how the protocol evolved from trial activation to its closure.

The submitted protocol is Ver 1.6. Revision, Amendment, and Memorandum are defined in "23.1 Modification of the Protocol" of the protocol.

The revisions of the protocol are as follows:

Ver. 1.1 (May 2017): Change in the case registration period

Ver. 1.2 (February 2018): Minor revisions to interim analysis period

Ver. 1.3 (May 2018): Change in the case registration period

Ver. 1.4 (November 2018): Revised procedures for handling SAEs and protocol changes in accordance with the revised Clinical Research Act
Ver. 1.5 (March 2021): Change in committee structure
Ver. 1.6 (July 2021): Addition of the method for collecting BRAF data for sub-analysis

Why was missing endpoint data not treated as a missing data problem rather than doing a complete case analysis?
We think that ITT should be used for the analysis of the primary endpoint. In our study, the DpR was set as the primary endpoint, so in the study design we did not set up an analysis using ITT, which included patients for whom the DpR could not be assessed. However, according to the reviewer's great suggestion, we performed an analysis using the ITT. The results showed that the DpR as the primary endpoint was significantly better in the cetuximab group. (the following Table)

	m-FOLFOXIRI + bevacizumab	m-FOLFOXIRI + cetuximab
n	180	179
Median DpR (%)<br>【Range】	46.0<br>【-50.1~100】	55.9<br>【-120.2～100】
Welch’s t-test	p=0.0437	

We have added this important point to the discussion since the lack of ITT analysis in this study is a major limitation.

It would be useful to see histograms of the DpR values for both treatment groups. That would help with evaluating the impact of outliers and be informative about the distribution of the DpR values.
Thank you for your valuable suggestions. Here is the histogram regarding DpR.
As the reviewer pointed out, we were able to confirm the distribution of the DpR values. In many studies on mCRC, DpR is shown in water-fall plots. Therefore, our paper also showed a waterfall plot and does not include a histogram.

m-FOLFOXIRI+Bev arm

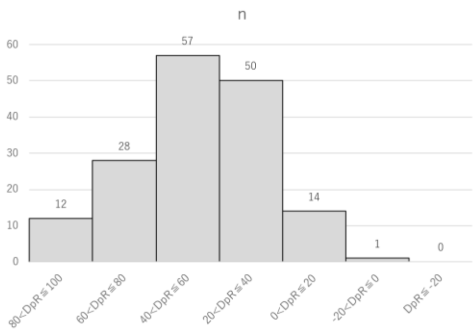

m-FOLFOXIRI+Cet arm

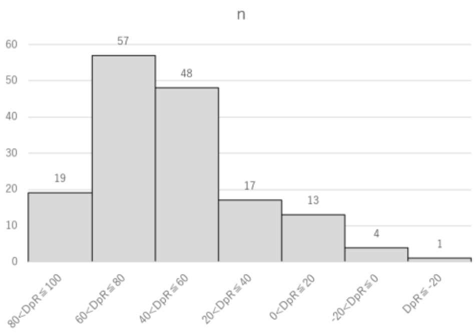

- Failure to justify why DpR is a surrogate for overall survival diminishes the significance.

Previous CRC trials comparing anti-EGFR antibody drugs with bevacizumab have found significant differences in OS, but almost all trials have found no difference in PFS. When comparing anti-EGFR antibody drugs with bevacizumab for *RAS* wild-type mCRC, PFS may not be appropriate as an endpoint. If OS is used as an endpoint, the prognosis of mCRC is so long that it takes much time to get results and the effect of a new treatment cannot be evaluated quickly. Therefore, we set the DpR, which has been shown to correlate with OS in clinical trials of mCRC, as the endpoint for this study.

Reviewers' Comments:

Reviewer #1:

Remarks to the Author:

I thank the reviewers for submission of their revised manuscript for review. This trial evaluates regimens that are of interest and could help the field move forward when thinking about how to maximally intensify therapy for patients with mCRC. Many of the comments I have outlined in my original review were also highlighted by other reviewers and continue to be substantial barriers to interpretation of this clinical trial.

Major issues:

1) The authors have planned sub-group analysis based on BRAF mutation status in their response to comments letter. Knowledge about BRAF mutations is essential to interpret this trial and I would suggest they resubmit their manuscript once this data is available. This is a critical flaw given the current standard of care for BRAF mutant CRC globally. They do have a subgroup with known BRAF status which is helpful. No response to this comment is needed but it is a major deficiency given current standards of care.

2) The authors state that they chose depth of response as a surrogate end point for OS because PFS is misleading. This is contrary to multiple studies evaluating surrogate endpoints for colorectal cancer and what the field has accepted as a surrogate endpoint for two decades. This accompanied by the analysis being per protocol are major methodologies flaws.

3) Please confirm if the PFS and OS analyses were performed in the PPS or ITT population. These should not be affected by many of the issues that caused patients to be excluded in the PPS end point so could be more prominently featured in the results.

4) The abstract conclusion is too strong given that the triplet plus cetuximab did not improve survival, only depth of response. Please make this more gentle.

5) The high rate of R0 resection suggests many patients entered the trial with resectable disease and is a limitation already noted. I am highlighting it here as the authors have submitted to a new journal. No further response to this comment is required but it is a methodology flaw.

6) I really like the new waterfall plots, they are very useful, however there are a mix of colours for each bar with some purple and some orange, however each color is supposed to represent a different therapy. Perhaps this was a PDF conversion issue, but I think it needs to be fixed.

7) I do not think Figure 3 should be the subgroup of a non-primary endpoint and would suggest including the PFS and OS curves for the overall population as well in the main set of figures. I would be ok with including this subgroup if the main data was also presented as part of the main figures but it is currently misleading.

Reviewer #2:

Remarks to the Author:

The results of the DEEPER study are in line with previuos comparisions of Bevacizumab vs Cetuximab in RASwt mCRC. It is noteworthy, that this is the first comparison with a FOLFOXIRI backbone in the RASwt setting. The primary endpoint, which is DoR has shown to correlate nicely with OS. Especially better than PFS! For the size of the study, DoR is an early endpoint which helps to provide valuable insights to the efficacy of the respective treatments.

Overall the authors have answered all comments sufficiently and the manuscript has improved by adding the clear statements on the nature of the analyses (exploratory vs confirmatory). Furthermore, the discussion is now well balanced.

Reviewer #3:

Remarks to the Author:

The authors made some attempts to address my comments but issues still remain. Primarily:

How multiplicity was handled should be defined in the statistical analysis section not just briefly mentioned in the discussion. In the revised version, this issue is largely swept under the rug and the response to the comment does not discuss multiplicity concerns.

The analysis plan has no mention of missing data which is strange and in general the handling of missing data is suboptimal. In their response, the authors performed what

they call ITT analysis but it is unclear how the ITT analysis accounts for the missing outcome data and I could not find a reference to that analysis in the manuscript.

Response to Reviewers

Reviewer #1 (Remarks to the Author):

I thank the reviewers for submission of their revised manuscript for review. This trial evaluates regimens that are of interest and could help the field move forward when thinking about how to maximally intensify therapy for patients with mCRC. Many of the comments I have outlined in my original review were also highlighted by other reviewers and continue to be substantial barriers to interpretation of this clinical trial.

Major issues:

1) The authors have planned sub-group analysis based on BRAF mutation status in their response to comments letter. Knowledge about BRAF mutations is essential to interpret this trial and I would suggest they resubmit their manuscript once this data is available. This is a critical flaw given the current standard of care for BRAF mutant CRC globally. They do have a subgroup with known BRAF status which is helpful. No response to this comment is needed but it is a major deficiency given current standards of care.

As the reviewer have indicated, *BRAF* status is an essential consideration in the treatment of mCRC. We believe that *BRAF* status information is very important in studies for *RAS* wild-type mCRC.

This study was initiated at a time when *BRAF* mutation measurement was not standardized, and therefore *BRAF* mutation could not be measured in many cases. The *BRAF* status data in this paper were collected from cases with *BRAF* test results performed in clinical practice after the study began, which was only about 50% of the cases.

We plan to measure *BRAF* mutations in patients who are able to donate tissue in the accompanying biomarker study, but the number of patients who will be found to have *BRAF* mutations cannot be increased significantly.

Subgroup analysis by *BRAF* status was not pre-planned in the design of this study, and analysis by *BRAF* mutation is exploratory. We have described this point as limitation.

Our paper reports the primary endpoint of the DEEPER trial and focuses on the *RAS* wild-type patients, which are the primary group analyzed. Furthermore, we believe that exploratory data on *BRAF* wild-type patients would be clinically important, so we have included the results in this manuscript.

2) The authors state that they chose depth of response as a surrogate end point for OS because PFS is misleading. This is contrary to multiple studies evaluating surrogate endpoints for colorectal cancer and what the field has accepted as a surrogate endpoint for two decades. This accompanied by the analysis being per protocol are major methodologies flaws.

We choosed the DpR as the primary endpoint of this study for the following reasons: historical control data for PFS and OS of triplet plus cetuximab were not available at the time of planning this study; the OS of triplet plus bevacizumab was approximately 36 months long and the sample size was too large to test the OS superiority of triplet plus cetuximab; several clinical trials have shown that in *RAS* wild-type mCRC, there was no difference in PFS between bevacizumab and anti-EGFR antibody combination regimens. However, as the reviewer pointed out, we were unable to show a correlation between DpR and survival in *RAS* wild-type mCRC. It may not have been appropriate to use DpR as an endpoint.

We have added the above to the method section.

3) Please confirm if the PFS and OS analyses were performed in the PPS or ITT population. These should not be affected by many of the issues that caused patients to be excluded in the PPS end point so could be more prominently featured in the results.

PFS and OS analyses were performed in the PPS population. We did not collect *BRAF* status information in the ITT population and are unable to perform the analysis for *RAS*/*BRAF* wild-type patients in the ITT population.

We apologize for the inconvenience.

4) The abstract conclusion is too strong given that the triplet plus cetuximab did not improve survival, only depth of response. Please make this more gentle.

Thank you for the reviewer's valuable suggestion.

We have revised the conclusion of the abstract.

"m-FOLFOXIRI + cetuximab has clinical benefit for tumor shrinkage in *RAS* wt mCRC. The survival benefit for *RAS*/*BRAF* wt and left-sided mCRC needs further investigation."

5) The high rate of R0 resection suggests many patients entered the trial with resectable disease and

is a limitation already noted. I am highlighting it here as the authors have submitted to a new journal. No further response to this comment is required but it is a methodology flaw.

We believe that the R0 resection rate in this study is not so high. Actually, similar data have been reported in previous clinical studies [JAMA Oncol. 2017 Jul 13;3(7):e170278, World J Surg Oncol. 2022 Aug 15;20(1):258].

It would be clinically important to analyze the resection rate when comparing anti-EGFR antibody drugs and bevacizumab in mCRC. The analysis of resection rate in *RAS* wild-type patients was pre-planned for this study, but the analysis of resection rate in *RAS/BRAF* wild-type and left-sided patients is exploratory, and we emphasize this point in the description of this study.

6) I really like the new waterfall plots, they are very useful, however there are a mix of colours for each bar with some purple and some orange, however each color is supposed to represent a different therapy. Perhaps this was a PDF conversion issue, but I think it needs to be fixed.

Thank you for the reviewer's kind instruction.

We have modified the design of Figure 2 to make the water-fall plot more readable.

7) I do not think Figure 3 should be the subgroup of a non-primary endpoint and would suggest including the PFS and OS curves for the overall population as well in the main set of figures. I would be ok with including this subgroup if the main data was also presented as part of the main figures but it is currently misleading.

We included the results of the PFS and OS analysis in the PPS population into Figure 3.

Reviewer #3 (Remarks to the Author):

The authors made some attempts to address my comments but issues still remain. Primarily:

How multiplicity was handled should be defined in the statistical analysis section not just briefly mentioned in the discussion. In the revised version, this issue is largely swept under the rug and the response to the comment does not discuss multiplicity concerns.

Thank you very much for the reviewer's valuable comments.

First of all, we apologize that we have not been able to adequately respond to your valuable suggestions.
We have added the following to the statistics section.
"Among patients with *BRAF* wild-type tumors, the cetuximab and bevacizumab groups were compared for patients with left-sided primary lesions. The sidedness was considered as a stratification factor in the primary analysis, however, *BRAF* status was not included. This subgroup analysis for *RAS*/*BRAF* wild-type and left-sided patients was a post-hoc analysis and repeated the same analysis performed on the PPS population for the DpR, PFS, and OS, so it did not take into account multiplicity, which should be done."

The analysis plan has no mention of missing data which is strange and in general the handling of missing data is suboptimal. In their response, the authors performed what they call ITT analysis but it is unclear how the ITT analysis accounts for the missing outcome data and I could not find a reference to that analysis in the manuscript.
Because our study defined the analysis of endpoints to be performed in the PPS, the definition of the ITT group and the handling of missing data were not made sufficiently clear. The statistical analysis plan for this study did not include an ITT analysis, and the missing data including *BRAF* status, makes it difficult to show an exploratory ITT analysis.
We have added a note to the limitation regarding these matters.

Reviewers' Comments:

Reviewer #1:
Remarks to the Author:
Thank you for your revisions. The authors have responded in a similar fashion to my critical flaws in the manuscript and analysis. Many of these are flaws in the design of the study and not necessarily addressable now that the study is complete.

However, other responses try to escape doing reasonable new analyses. The authors have said some things like they are not doing a BRAF analysis because that was not pre-planned, and yet many of the other analyses were not pre-planned as well.

Reviewer #2:
Remarks to the Author:
The authors have answered all comments from the reviewers.

In my view, no additional data nor information is needed for publication of the clinical interesting data.

Reviewer #3:
Remarks to the Author:
I thank the authors for their response. The two comments about multiplicity and missing data still remain unaddressed.

Currently, it says "This subgroup analysis for RAS/BRAF wild-type and left-sided patients was a post-hoc analysis and repeated the same analysis performed on the PPS population for the DpR, PFS, and OS, so it did not take into account multiplicity, which should be done. " If it should be done, why was it not done? If the authors insist on not adjusting for multiplicity they should state that the results should be interpreted as an exploratory analysis.

For missing data, I am requesting that the authors do an analysis that appropriately accounts for missing data. This includes reporting how much missing data there was in variables that were being analyzed and if there was missing data accounting for in the analyzes using appropriate statistical methods. Currently, the manuscript states "The statistical analysis plan for this study did not include an ITT analysis, and the missing data including BRAF status, makes it difficult to show an exploratory ITT analysis." and

from this it is impossible to understand the impact of missing data and how it was handled (and overall this sentence is confusing).

Response to Reviewers

Reviewer #1 (Remarks to the Author):

Thank you for your revisions. The authors have responded in a similar fashion to my critical flaws in the manuscript and analysis. Many of these are flaws in the design of the study and not necessarily addressable now that the study is complete.

However, other responses try to escape doing reasonable new analyses. The authors have said some things like they are not doing a BRAF analysis because that was not pre-planned, and yet many of the other analyses were not pre-planned as well.

Thank you so much for the reviewer's valuable comments.
This manuscript is an exploratory analysis using currently available BRAF mutation data. The DEEPER trial started in 2015, when BRAF testing was not available in clinical practice. Subsequently, the BRAF testing was approved during the trial, allowing BRAF status to be measured in routine clinical practice. Therefore, BRAF V600E data were available from electronic health records only for about 50% of patients. We intend to conduct further analyses in the future when we are able to add BRAF mutation data in further biomarker studies. We will get the updated BRAF mutation data from the biomarker study next year.
To examine the impact of the presence or absence of BRAF data, we analyzed patient background between the population with BRAF data and the overall population or without BRAF data and found no differences in patient background between these patient groups. There were also no significant differences in point estimates and confidence intervals for OS, PFS, and ORR (see table below).
According to the reviewer's valuable indication, we modified the Result section.

Reviewer #3 (Remarks to the Author):

I thank the authors for their response. The two comments about multiplicity and missing data still remain unaddressed.

Currently, it says "This subgroup analysis for RAS/BRAF wild-type and left-sided patients was a post-hoc analysis and repeated the same analysis performed on the PPS population for the DpR, PFS, and OS, so it did not take into account multiplicity, which should be done. " If it should be done, why

was it not done? If the authors insist on not adjusting for multiplicity they should state that the results should be interpreted as an exploratory analysis.

We really thank the reviewer for valuable suggestion.
In order to avoid strong expressions such as "significant," we have revised the Results to include only point estimates and 95% confidence intervals, rather than p-values, in the results.

For missing data, I am requesting that the authors do an analysis that appropriately accounts for missing data. This includes reporting how much missing data there was in variables that were being analyzed and if there was missing data accounting for in the analyzes using appropriate statistical methods. Currently, the manuscript states "The statistical analysis plan for this study did not include an ITT analysis, and the missing data including BRAF status, makes it difficult to show an exploratory ITT analysis." and from this it is impossible to understand the impact of missing data and how it was handled (and overall this sentence is confusing).

The DEEPER trial started in 2015, when BRAF testing was not available in clinical practice. Subsequently, the BRAF testing was approved during the trial, allowing BRAF status to be measured in routine clinical practice. Therefore, BRAF V600E data were available from electronic health records only for about 50% of patients. To examine the impact of the presence or absence of BRAF data, we analyzed patient background between the population with BRAF data and the overall population or the population without BRAF data and found no differences in patient background between these patient groups. There were also no significant differences in point estimates and confidence intervals for OS, PFS, and ORR (see table below).
We asked the biostatisticians (Kyongsun Pak; Division of Biostatistics, Center for Clinical Research and Development , National Center for Child Health and Development) at JACCRO for their opinion, and since this was a post hoc analysis, we considered the group differences rather than adjusting for multiplicity of P values.
According to the reviewer's valuable indication, we modified the Result section.

	PPS		Full analysis set		PPS with BRAF data	
	**m-FOLFOXIRI + cetuximab** (n=159)	**m-FOLFOXIRI + bevacizumab** (n=162)	**m-FOLFOXIRI + cetuximab** (n=175)	**m-FOLFOXIRI + bevacizumab** (n=173)	**m-FOLFOXIRI + cetuximab** (n=80)	**m-FOLFOXIRI + bevacizumab** (n=87)
Sex, n (%)<br>Male/Female	101 (63·5)/58 (36·5)	106 (65·4)/56 (34·6)	109 (62.3)/66 (37.7)	113 (65.3)/60 (34.7)	49 (61.3)/31 (38.8)	55 (63.2)/32 (36.8)
Age (years), median (range)	65 (26–83)	65 (29–85)	65 (26–83)	65 (29–85)	65 (26–83)	65 (29–80)
ECOG performance status, n (%)<br>0/1	146 (91·8)/13 (8·2)	146 (90·1)/16 (9·9)	160 (91.4)/15 (8.6)	157 (90.8)/16 (9.2)	73 (91.3)/7 (8.8)	79 (90.8)/8 (9.2)
Adjuvant chemotherapy, n (%)<br>None/With oxaliplatin/Without oxaliplatin	148 (93·1)/7 (4·4)/4 (2·5)	152 (93·8)/6 (3·7)/4 (2·5)	161 (92.0)/8 (4.6)/6 (3.4)	159 (91.9)/9 (5.2)/5 (2.9)	77 (96.3)/1 (1.3)/2 (2.5)	82 (94.3)/3 (3.4)/2 (2.3)
Primary tumor site, n (%)<br>Colon/Rectum	91 (57·2)/68 (42·8)	93 (57·4)/69 (42·6)	100 (57.1)/75 (42.9)	100 (57.8)/73 (42.2)	46 (57.5)/34 (42.5)	52 (59.8)/35 (40.2)
Site of primary tumor, n (%)<br>Right/Left	27 (17·0)/132 (83·0)	25 (15·4)/137 (84·6)	31 (17.7)/144 (82.3)	30 (17.3)/143 (82.7)	15 (18.8)/65 (81.3)	15 (17.2)/72 (82.8)
Resected primary tumor, n (%)<br>Yes/No	83 (52·2)/76 (47·8)	86 (53·1)/76 (46·9)	90 (51.4)/85 (48.6)	95 (54.9)/78 (45.1)	42 (52.5)/38 (47.5)	54 (62.1)/33 (37.9)
Time to metastases, n (%)<br>Synchronous/Metachronous	136 (85·5)/23 (14·5)	136 (84·0)/26 (16·0)	148 (84.6)/27 (15.4)	142 (82.1)/31 (17.9)	70 (87.5)/10 (12.5)	68 (78.2)/19 (21.8)
Number of metastatic sites, n (%)<br>0-1/>1	55 (34.6)/104 (65.4)	80 (49.4)/82 (50.6)	55 (31.4)/120 (68.6)	85 (49.1)/88 (50.9)	24 (30.0)/56 (70.0)	40 (46.0)/47 (54.0)
Liver-only disease, n (%)<br>Yes/No	39 (24.5)/120 (75.5)	50 (30.9)/112 (69.1)	39 (22.3)/136 (77.7)	53 (30.6)/120 (69.4)	18 (22.5)/62 (77.5)	29 (33.3)/58 (66.7)
Metastatic sites, n (%)<br>Liver<br>Lung<br>Lymph node<br>Peritoneum<br>Bone	 119 (74.8)<br>44 (27.7)<br>98 (61.6)<br>31 (19.5)<br>3 (1.9)	 116 (71.6)<br>37 (22.8)<br>81 (50.0)<br>25 (15.4)<br>4 (2.5)	 128 (73.1)<br>47 (26.9)<br>114 (65.1)<br>39 (22.3)<br>4 (2.3)	 123 (71.1)<br>39 (22.5)<br>87 (50.3)<br>26 (15.0)<br>4 (2.3)	 65 (81.3)<br>27 (33.8)<br>48 (60.0)<br>16 (20.0)<br>2 (2.5)	 69 (79.3)<br>18 (20.7)<br>44 (50.6)<br>12 (13.8)<br>2 (2.3)
BRAF V600E status<br>wild / mutant / unknown	72 / 8 / 79	81 / 6 / 75	79 / 11 / 85	87 / 6 / 80	72 / 8 / 0	81 / 6 / 0

Cohort	Arm	N	DpR Median (SD)	PFS, event	PFS month Median (95%CI)	HR (95%CI)	OS, event	OS month Median (95%CI)	HR (95%CI)	ORR (95%CI)	OddRatio (95%CI)
Full analysis set	Bev	N=173	no data	156/173	12.2 (11.1–13.2)		106/173	41.6 (33.5–46.7)		67.1% (60.0-74.1)	
	Cet	N=175	no data	152/175	12.3 (10.8–13.8)	0.946 (0.756-1.183)	104/175	37.6 (30.1–48.2)	1.038 (0.792-1.361)	65.1% (58.1-72.2)	0.92 (0.59-1.43)
PPS	Bev	N=162	46.0% (20.5309)	147/162	12.3 (11.3–14.0)		97/162	42.1 (34.5–47.7)		69.1% (62.0-76.2)	
	Cet	N=159	57.3% (26.1702)	136/159	13.0 (11.3–14.5)	0.887 (0.702-1.121)	88/159	42.9 (35.1–53.1)	0.941 (0.705-1.256)	71.1% (64.0-78.1)	1.10 (0.68-1.77)
PPS with BRAF data	Bev	N=87	47.7% (20.5651)	81/87	11.7 (10.8-14.0)		50/87	40.2 (33.5-46.7)		71.3% (61.8-80.8)	
	Cet	N=80	59.2% (24.8273)	70/80	13.4 (11.3-15.1)	0.857 (0.622-1.181)	37/80	52.4 (39.6-56.0)	0.780 (0.510-1.194)	76.3% (66.9-85.6)	1.29 (0.65-2.59)
PPS without BRAF data	Bev	N=75	42.2% (20.3848)	66/75	12.8 (11.1-14.6)		47/75	44.3 (30.7-55.5)		66.7% (56.0-77.3)	
	Cet	N=79	53.9% (27.3719)	66/79	12.6 (9.7-15.2)	0.928 (0.659-1.307)	51/79	37.0 (26.7-50.2)	1.090 (0.733-1.621)	65.8% (55.4-76.3)	0.96 (0.49-1.88)

Reviewers' Comments:

Reviewer #1:
Remarks to the Author:
No further comments from me. Thank you for allowing me to review this manuscript.

Reviewer #2:
Remarks to the Author:
Within the DEEPER trial which started in 2015 te primary endpoint was depth of response within the RAS wildtype mCRC population. The RASwt cohort, including BRAFV600E mutant cases, was and still is the mCRC population acording to the label of anti-EGFR antibodies (Cetuximab or Panitumumab).
The primary endpoint was met, FOLFOXIR plus Cetuximab leads to a significan better depth of response. This has not been shonw before in the respective population.Secondary endpoint, such as PFS and OS were clearly marked as exploratory endpoints, which is also clear for th erequested RAS/BRAFwt population. Due to reviewers request, the authos were able to gather data on roughly half of the total population with respect to BRAFV600E mutations. The exploratory analysis of these roughly 160 patients showed, that PFS and OS is invcreased in this more selected poulation. This is quite expectable as newwer data of hyperselected (PARADIGM study Shitara et al Nat Med. 2024 Mar;30(3):730-739.) or ultraselected cohorts (Randon G et al JCO Precis Oncol. 2022 Apr:6:e2200037.) show better outcomes in the respective and selected cohort.

In my view, the manuscript is publishable in the current form.

Reviewer #3:
Remarks to the Author:
The multiplicity issue has been addressed. But the missing data issue still remain and I don't see any reason for why the analysis that is currently performed is a satisfactory way to adjust for missing data. I have brought this issue up several times but the authors seem not to be willing to re-do the analysis.

"We asked the biostatisticians (Kyongsun Pak; Division of Biostatistics, Center for Clinical Research and Development , National Center for Child Health and Development) at JACCRO for their opinion, and since this was a post hoc analysis, we considered the group differences rather than adjusting for multiplicity of P values." I

don't understand how this comment relates to not adjusting for missing data

"According to the reviewer’s valuable indication, we modified the Result section." The bottom line is that the analysis was not changed and any other edits are irrelevant.

Response to Reviewers

Reviewer #3 (Remarks to the Author):

The multiplicity issue has been addressed. But the missing data issue still remain and I don't see any reason for why the analysis that is currently performed is a satisfactory way to adjust for missing data. I have brought this issue up several times but the authors seem not to be willing to re-do the analysis.

"We asked the biostatisticians (Kyongsun Pak; Division of Biostatistics, Center for Clinical Research and Development , National Center for Child Health and Development) at JACCRO for their opinion, and since this was a post hoc analysis, we considered the group differences rather than adjusting for multiplicity of P values." I don't understand how this comment relates to not adjusting for missing data

"According to the reviewer’s valuable indication, we modified the Result section." The bottom line is that the analysis was not changed and any other edits are irrelevant.

Thank you so much for the reviewer’s valuable comments.
In the previous revision, we have revised the Results to include only point estimates and 95% confidence intervals, rather than p-values, in the results, according to the reviewers’ comments.
We checked the detailed missing data in our study. The level of missingness was low (4%); the number of patients with missing data was 15 in the ITT population. Therefore, we conducted a complete case analysis (next page Table). We have rewritten the manuscript (Discussion section) and added a supplement table so that readers can understand the details of missing data.

	m-FOLFOXIRI + cetuximab	m-FOLFOXIRI + bevacizumab
ITT population		
Total number of patients (n)	179	180
Patients excluded from ITT analysis (n)	12	11
Reasons for the exclusion (patients with no data of DpR)		
No administration (no protocol treatment)	4*	4
No target lesions	0	3
Not evaluated during protocol treatment	7	4
Not evaluated in a prescribed manner	1	0
**Patients evaluable for ITT analysis**	**167**	**169**
Median DpR (range)	55·9% (-120.2–100)	46·0% (-0.50.1–100)
Mean (95% CI)	52·3% (47·6-57·1)	46·3% (43·0–49·7)
Standard deviation (95% CI)	31·10 (28·1-34·9)	22·29 (20·1–25·0)
Welch's t-test	p=0·044	
⇩		
Patients who do not meet the definition of DpR analysis	8	7
Reasons for not evaluable for DpR		
Could not be evaluated by external review board due to lack of submission of image data	2	5
Progression of non-target lesions detection of new lesions	6	2
⇩		
PPS population		
**Patients evaluable for PPS analysis**	**159**	**162**
Median DpR (range)	57·3% (-42·6–100)	46·0% (-0.6–100)
Mean (95% CI)	55·2% (51·1–59·3)	47·3% (44·1–50·5)
Standard deviation (95% CI)	26·17 (23·6–29·4)	20·53 (18·5–23·0)
Welch's t-test	p=0·0029	
